# Visible Light–Driven Cascade Carbon–Carbon Bond Scission for Organic Transformations and Plastics Recycling

**DOI:** 10.1002/advs.201902020

**Published:** 2019-10-24

**Authors:** Sarifuddin Gazi, Miloš Đokić, Kek Foo Chin, Pei Rou Ng, Han Sen Soo

**Affiliations:** ^1^ Division of Chemistry and Biological Chemistry School of Physical and Mathematical Sciences Nanyang Technological University 21 Nanyang Link Singapore 637371 Singapore; ^2^ Department of Chemistry School of Applied Sciences University of Science and Technology Techno City, Kling Road, Baridua 9th Mile Ri Bhoi Meghalaya 793101 India; ^3^ Solar Fuels Laboratory Nanyang Technological University 50 Nanyang Avenue Singapore 639798 Singapore

**Keywords:** artificial photosynthesis, cascade carbon—carbon bond cleavage, photoredox catalysis, plastics recycling, visible light

## Abstract

Significant efforts are devoted to developing artificial photosynthetic systems to produce fuels and chemicals in order to cope with the exacerbating energy and environmental crises in the world now. Nonetheless, the large‐scale reactions that are the focus of the artificial photosynthesis community, such as water splitting, are thus far not economically viable, owing to the existing, cheaper alternatives to the gaseous hydrogen and oxygen products. As a potential substitute for water oxidation, here, a unique, visible light–driven oxygenation of carbon—carbon bonds for the selective transformation of 32 unactivated alcohols, mediated by a vanadium photocatalyst under ambient, atmospheric conditions is presented. Furthermore, since the initial alcohol products remain as substrates, an unprecedented photodriven cascade carbon—carbon bond cleavage of macromolecules can be performed. Accordingly, hydroxyl‐terminated polymers such as polyethylene glycol, its block co‐polymer with polycaprolactone, and even the non‐biodegradable polyethylene can be repurposed into fuels and chemical feedstocks, such as formic acid and methyl formate. Thus, a distinctive approach is presented to integrate the benefits of photoredox catalysis into environmental remediation and artificial photosynthesis.

## Graphical abstract

A route to add value to unactivated alcohols, which are ubiquitous feedstocks often available from biomass, by a photocatalytic carbon—carbon bond cleavage and oxygenation, is presented. Notably, an unprecedented cascade carbon—carbon bond cleavage in both commercially available short‐chain alcohols and hydroxyl‐terminated polymers is demonstrated, further highlighting that this approach can potentially be extended to polyethylene‐backbone polymers found in microplastic pollutants.

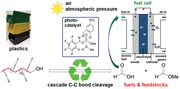

## 1 Introduction

**Figure 1:**
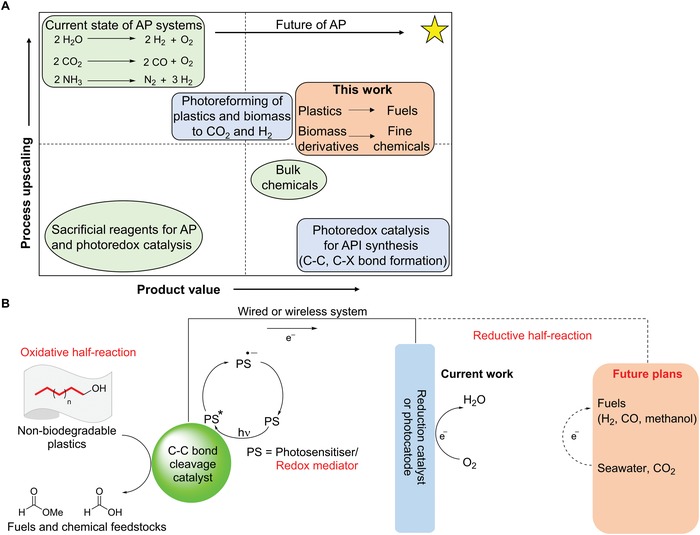
A) Comparisons between AP and photoredox catalysis in the context of scalability versus product value. This work is an advance toward the upper right quadrant, where we hope to achieve both large‐scale and economically attractive processes, as indicated by the yellow star. This figure was adapted from reference 24. B) Our proposed AP system in which water oxidation is replaced by an oxidative C—C bond cleavage to recycle and valorize non‐biodegradable plastics, while the reductive half‐reaction is presently O_2_ reduction, but can potentially be other photocatalytic or photoelectrocatalytic processes to produce other solar fuels.

Our current global energy demand is largely met by using finite resources, namely fossil fuels. However, by 2030, energy consumption is likely to rise by almost 20% to 23 TW.^1^, ^2^ If we continue to rely mainly on fossil fuels for energy production, the large amounts of greenhouse gases that will be produced could lead to catastrophic environmental pollution and global climate change. To avert this looming energy and environmental crisis, there is substantial and increasing support worldwide for the use of more sustainable and renewable energy sources. In this context, significant efforts have been devoted to the creation of unconventional artificial photosynthetic (AP) systems that harness solar energy to produce chemicals and fuels.^3^ AP offers the advantage of directly storing the energy from sunlight in more fungible forms such as liquid fuels that can be directly introduced into existing infrastructure. Many processes that have dominated the attention of the AP community are simple and scalable, such as water splitting, and often comprise radical‐based elementary steps. Although such systems produce valuable H_2_ as a fuel in the reductive half‐reaction, current oxidative half‐reactions of AP systems usually generate products of low economic value (e.g., O_2_ from water oxidation, **Figure** **1**A), which has hampered the commercialization of these technologies.

Recently, radical‐based reactions, in the form of photoredox catalysis,^4^, ^5^, ^6^, ^7^, ^8^, ^9^, ^10^ have emerged as attractive, novel routes for organic synthesis and fine chemicals production, which are at the opposite end of the chemical value chain to AP systems now (Figure 1A). The light harvesters employed often include expensive iridium or ruthenium polypyridyl complexes and their derivatives, and the reactions are usually conducted on comparatively smaller scales. The natural products or active pharmaceutical ingredients (APIs) from these photoredox reactions are often a few orders of magnitude more expensive than the precursors, which has justified the use of expensive platinum group metal photocatalysts. For instance, the respiratory stimulant etamivan costs US$650 g^−1^ and can be synthesized from 3,4‐dimethoxybenzaldehyde (US$8 g^−1^), which we show in this paper in the following sections. Notably, apart from the seminal work by MacMillan and co‐workers^11^ on the decarboxylation followed by tandem C—C^12^ or C—X (X = O, N, and S)^13^ bond formation reactions, photoredox catalysis has rarely been utilized in the field of selective C—C bond cleavage despite its potential in the late‐stage deconstructive functionalization of complex organic molecules or the valorization and recycling of lignin^14^, ^15^ and plastics, for example. Wang and co‐workers^16^, ^17^ have reported the photocatalytic hydrogenolysis of lignin model compounds, whereas Sarpong and co‐workers,^18^ Dong and co‐workers,^19^ Knowles and co‐workers,^20^ and Zuo and co‐workers^21^ have lately presented the C—C activation of cyclic amines, ketones, or alcohols, followed by derivatization under photoredox or thermal conditions. A few reports have described the incidental C—C activation in reaction intermediates and substrates.^19^, ^22^, ^23^ However, the intentional application to a broad range of small, acyclic, aliphatic alcohols, and cascade photoredox catalysis by C—C activation and functionalization of macromolecules remains unknown. Most critically, photoredox catalysis remains in the domain of small‐scale niche processes, such as the production of APIs (Figure 1A).

Lately, Reisner^24^ revived the concept of bringing AP and radical catalysis closer together to increase process scalability while generating more value‐added products (Figure 1A). This concept had been pioneered by Meyer and co‐workers, who demonstrated its potential for chloride^25^ and benzyl alcohol oxidation with simultaneous H_2_ evolution.^26^ Others, including Sun and co‐workers,^27^, ^28^, ^29^, ^30^ Meyer and co‐workers,^31^, ^32^, ^33^, ^34^, ^35^ Berlinguette and co‐workers,^36^, ^37^, ^38^ Reisner and co‐workers,^14^, ^39^, ^40^, ^41^ and Lei and co‐workers,^42^, ^43^, ^44^ have subsequently shown that the gap between AP and radical catalysis can potentially be bridged via the valorization of biomass, functionalization of amines, oxidation of other halides, photo‐electrochemical reduction of CO_2_, and even photoreforming of plastics.

Plastics have become essential in our daily lives owing to their low price, versatility, and ease of production. However, many of them are non‐biodegradable and are causing disastrous environmental problems on land and also in the oceans and waterways.^45^ Studies have shown that microplastic fibers have been found even in deep‐sea organisms,^46^ reaffirming the consequences that non‐biodegradable plastics can have even on some of the most remote ecosystems. While plastics recycling has been gaining tremendous support worldwide in the past few years, a large portion of non‐biodegradable polymers is not recovered and recycled in an eco‐friendly way. Instead, the plastic materials are often degraded by combustion or pyrolyzed to produce H_2_ and small amounts of short‐chain olefins,^47^, ^48^, ^49^ with significant amounts of CO_2_ being generated as well. Alternatively, biodegradable polymers are often more expensive to produce, and are suitable only for specific applications.^50^, ^51^ These issues highlight the importance of developing more sustainable strategies to not only degrade, but also repurpose persistent plastic waste without generating new pollutants.

Although the photoreforming of plastics to produce H_2_ fuel as a clean fuel presents an appealing approach to overcome the environmental problems and concurrently add value to AP (Figure 1A), nonetheless, earlier studies employed toxic, Cd‐based quantum dots as the photocatalysts, and the plastics were converted into intractable organic mixtures,^41^ both of which are undesirable. Moreover, they only achieved conversions of about 40% for specific plastics such as polyurethane and polyethylene terephthalate, and their process, required alkaline pretreatment to partially hydrolyze the carbamate and ester linkages.^41^ Though Reisner and co‐workers' most recent work on the photoreforming of plastics with CN*_x_*/Ni_2_P catalysts presents an innovative advance that overcame the toxicity of their original Cd‐containing photocatalysts,^52^ their process still required alkaline pretreatment, while conversions remained below 50% and multiple products with modest selectivity were observed.

**Figure 2:**
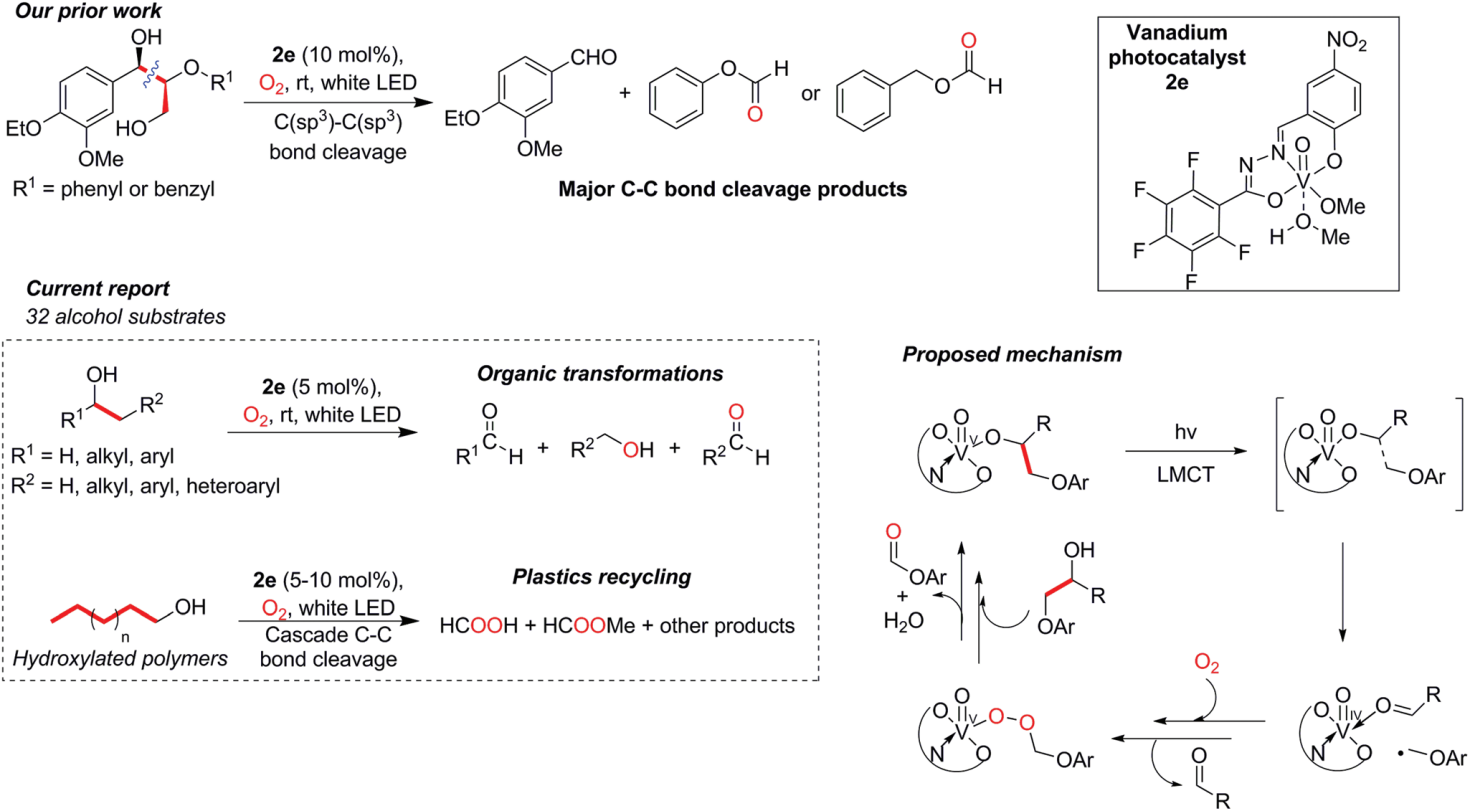
Our group's previously reported studies on C—C bond cleavage in lignin models along with the proposed mechanism, structure of our optimal photocatalyst **2e**, and this paper's applications in multiple fields, including a demonstration in recycling non‐biodegradable plastics, such as polyethylene, via cascade C—C bond cleavage. The bonds that undergo cleavage are highlighted in bold and red. Details for the syntheses of our vanadium photocatalysts are depicted in Figure S1 (Supporting Information).

Against this backdrop, our group has been actively pursuing photoredox catalytic processes that can generate value‐added organic precursors^53^, ^54^ or degrade pollutants,^55^ and can eventually be integrated into AP systems (Figure 1B). Notably, we developed vanadium(V) photocatalysts (**2a–2f**, Figure S1, Supporting Information) that can selectively cleave the activated C—C bond adjacent to the benzylic alcohol in lignin model substrates (**Figure** **2**).^56^, ^57^ We used isotope‐labeling, product analysis, spectroscopy, and density functional theory (DFT) calculations to show that **2a**–**2f** operated as some of the first molecular instances of photocatalysts that absorb light via ligand‐to‐metal charge transfer (LMCT) chemistry owing to the redox noninnocence of the hydrazone–imidate ligands (Figure 2).^56^ The C—C bond of a coordinated alcohol would cleave to produce a carbonyl group as one of the primary products, while the other radical fragment would react with dissolved O_2_ in air to eventually give another oxygenated compound. These initial products could be further oxidized or hydrolyzed under the aerobic reaction conditions to become other oxygenated derivatives, including alcohols, aldehydes, esters, and carboxylic acids, which are all versatile and valuable precursors that can undergo subsequent transformations via established reactions. For example, one of our reaction products is phenyl formate, which costs around US$21 mL^−1^, and can potentially be extracted from nonfood biomass lignin that is typically discarded as waste or combusted as cheap fuel.

Among these oxygenated compounds, alcohols, many of which can be obtained commercially from biomass and natural products as affordable feedstocks, are appealing precursors to other value‐added downstream derivatives. One of the most common methods to functionalize alcohols is via oxidation to aldehydes and carboxylic acids, which mainly involves C—H bond activation reactions. In contrast, selective, catalytic oxidation of alcohols by C—C single bond cleavage is rare and has been a formidable problem in synthetic chemistry, but can potentially be a powerful tool in the late‐stage functionalization or recycling of alcohols in complex molecules and macromolecules. Several challenges include the poor selectivity of C—C bond activation^58^, ^59^, ^60^ over reactions in other functional groups, the lack of energetically compensating bond formation processes, and the kinetic inertness of the nonpolar C—C bond. Consequently, prior examples have often relied on the release of ring strain in small cyclic molecules such as cyclopropane, cyclobutane, and even cyclopentane,^61^ or the aromatization of prearomatic substrates.^62^ Moreover, thermal conditions are typically used on specialized substrates with specifically constructed functional groups to prevent undesired decomposition.^58^

Here, we apply **2e** in the photoredox, controlled C—C bond cleavage, and oxygenation of unactivated aliphatic alcohols, under ambient atmospheric conditions with air as the terminal oxidant. The products include oxygenated hydrocarbons, which are desirable precursors for organic synthesis. We further demonstrate that this strategy can be utilized in multiple fields, not only for organic syntheses, but also for the cascade C—C bond disassembly in alcohol‐terminated biodegradable and non‐biodegradable polymers, representing a significant advance in photoredox catalysis and environmental remediation.

## 2 Results and Discussion

### 2.1 Optimization of Conditions and Evaluation of Functional Group Tolerance

Owing to the large number of impurities and different functional groups in macromolecules and plastics, we sought to assess the functional group tolerance of our photocatalyst to determine the limitations of our system. Our condition optimization started with 1‐phenylpropan‐2‐ol (**3**) (Figure S2, Supporting Information), a commercially available pharmaceutical intermediate and flavor ingredient. Gratifyingly, the substrate readily underwent C—C bond cleavage reaching 89% conversion within 6 h in the presence of 5 mol% of **2e**. The primary products of the reaction should be benzyl alcohol (**4**) from oxygenation by air and acetaldehyde (**5**), which were obtained in moderate yields of 32% and 47%, respectively (Figure S2 (Entry 1), Supporting Information). We observed that the in situ oxidation of **4** led to benzaldehyde (**6**) in significant amounts as well (34%).

To prevent the overoxidation of **4** and preserve the selectivity of the reaction, hydrogen atom transfer (HAT) agents such as triphenylmethane, 9,10‐dihydroanthracene (9,10‐DHA), and 1,4‐cyclohexadiene (1,4‐CHD) were explored as antioxidants to inhibit reactive oxygen species (ROS) that arose from the use of air. Their effects on the selectivity are summarized in Figure S2 (Entries 2–5) (Supporting Information). Antioxidant 1,4‐CHD provided the best results in minimizing the overoxidation of **4**, while maintaining quantitative conversion and higher selectivity of the primary products. Although a moderate amount of 1,4‐CHD was required, additives or stoichiometric sacrificial reagents in photoredox catalysis, such as acids, bases, or terminal redox reagents, are commonly employed to improve chemoselectivity. For fine chemicals and API production by synthetic organic chemistry, the scales are smaller and the additional cost of these additives can be justified by the added value of the products. Additional parameters that we screened included the use of O_2_ instead of air, the catalyst loading, and the catalysts in our existing library (Figure S2 (Entries 6–13), Supporting Information). Moreover, we conducted a larger scale photolysis of **3** at ten times (136 mg of substrate) the standard optimized conditions and still observed full substrate conversion and respectable product yields, albeit with double the reaction time (Figure S2 (Entry 14), Supporting Information). Control experiments conducted in the absence of catalyst, light, and air also confirm that each of these is vital. These and other optimization details are compiled in Tables S1–S4 (Supporting Information).

Furthermore, we investigated whether electron acceptors other than O_2_ could be used for our photocatalytic system, to verify the feasibility of combining this unique oxidative cleavage of alcohols with a different reduction reaction for solar fuels' production in a future, integrated AP system. We chose hydrogen peroxide, *tert*‐butyl hydroperoxide (TBHP), and copper(II) triflate as representative oxidants to examine this possibility (Table S5, Supporting Information). While the peroxides were adequate alternatives to O_2_ for the photocatalytic C—C bond cleavage in **3**, the use of copper(II) triflate led to totally different reactivity with no products from the expected C—C bond cleavage of **3**. Notably, the conversion of **3** in the presence of the peroxides (Table S5 (Entries 1 and 2), Supporting Information) was slower than the reactions with O_2_. Therefore, our optimal reaction conditions include the use of acetonitrile as the solvent (0.50 mL), 0.10 × 10^−3^ m of substrate, ambient temperatures, 5 mol% of catalyst **2e**, 0.40 × 10^−3^ m of 1,4‐CHD, O_2_ as the terminal oxidant, and a 48 W white light‐emitting diode (LED) as the light source. In all the experiments with the other substrates, the photocatalytic reactions were conducted at least twice and usually thrice, with deviations from the optimal conditions indicated, where applicable.

With the optimized reaction conditions in hand, we proceeded to explore the functional group tolerance and the effects of various substituents on the reactivity and product distribution. Since many complex substrates and macromolecules contain multiple functionalities, it is imperative to examine the catalyst's performance in the presence of different functional groups. For this purpose, we employed a mechanism‐based substrate screening method recently reported by Glorius and co‐workers with additives rather than actual substrates.^63^ Instead of synthesizing all the derivatives of **3** that contain different functional groups, the corresponding benzene derivative was added to the reaction mixture in stoichiometric amounts. The photocatalyzed C—C bond cleavage reactions proceeded with essentially quantitative conversions and moderate‐to‐high yields of the aldehyde and alcohol products in the presence of a broad range of both electron‐donating and ‐withdrawing functional groups (Figure S3, Supporting Information). Electron‐donating methyl and methoxy, as well as electron‐withdrawing esters, halogens, nitro, and nitrile were all tolerated. Notably, the feasibility of halogenated aromatic compounds offers the opportunity of subsequent transformation with traditional transition metal‐catalyzed coupling chemistry. Even though anilines were found to inhibit the catalyst, with no improvements to the conversions after prolonged reaction times, the amine could be easily protected by a *tert*‐butyloxycarbonyl (Boc) group and the reaction proceeded smoothly (Figure S3 (Entry 8), Supporting Information). The broad functional group tolerance of our photocatalytic system encouraged us to investigate its applicability to more challenging alcohols.

**Figure 3:**
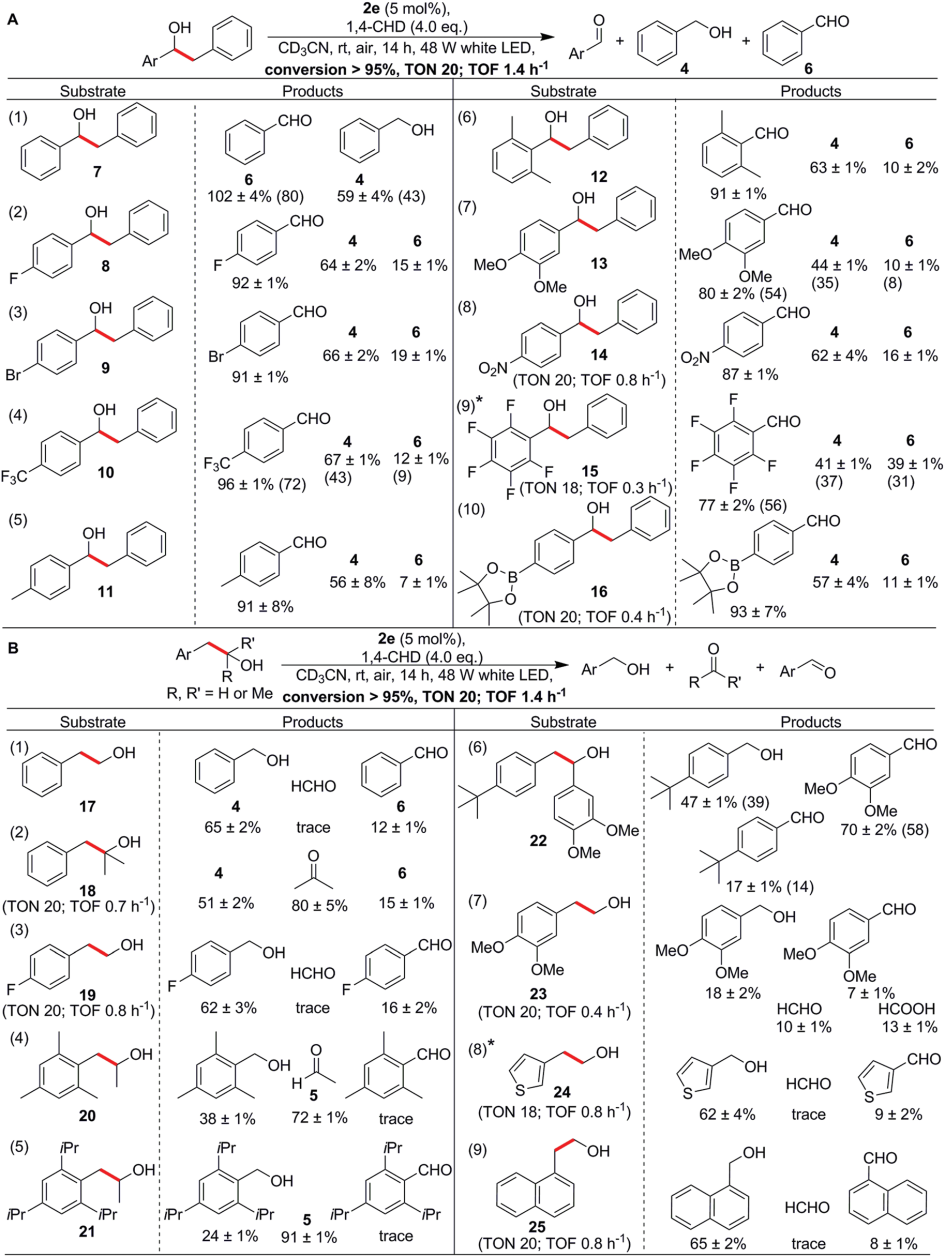
A) Effects of different functional groups on the rate and product distribution of the photocatalytic C—C bond cleavage in **7** and its derivatives. B) Examples of sterically demanding substrates **20** and **21**, as well as the presence of a heteroatom in **24** and fused rings in **25**. Product yields were determined by ^1^H NMR spectroscopy with 1,1,2,2‐tetrachloroethane as the internal standard. Reaction times were 24 h for substrates **14**, **19**, and **25**; 68 h for **15**; 48 h for **16**; 30 h for **18**; and 46 h for **23**. The bonds that undergo cleavage are highlighted in bold and red. The turnover numbers (TON) and turnover frequencies (TOF) are shown below the reaction arrow or specified when different. Some representative isolated yields are given in parentheses. *Conversion 90%.

Guided by the insights from the mechanism‐based screening, we then examined the reactivity of derivatives of **3**. We hypothesized that substrates which also generate stabilized benzyl radicals upon C—C bond cleavage should undergo facile photocatalytic reactions. To test this idea, we examined substrate **7** (**Figure** **3**A, Entry 1) and used its derivatives to rapidly expand the substrate library by introducing various functionalities on one of its aryl rings. Gratifyingly, **7** was fully converted into **4** and **6** within 14 h, and the over 100% yield of **6** likely arose from in situ overoxidation of **4**. In addition, as anticipated from the results of the mechanistic screening, electron‐withdrawing substituents hindered the reactivity and led to longer reaction times, but quantitative conversion was still achievable (Figure 3A, substrates **8–10** and **14–16**). Likewise, substrates with electron‐donating methyl and methoxy groups at the para, meta, and even the sterically demanding ortho positions reacted readily to provide moderate‐to‐high yields of the expected products under the standard conditions (Figure 3A, substrates **11–13**).

Furthermore, we chose substrates **17** and **18** (a primary and a tertiary alcohol, respectively), which would likewise generate benzyl radicals, and the reactions achieved high conversions within 14 and 30 h, respectively (Figure 3B, Entries 1 and 2), despite the steric hindrance expected from the latter. The reaction with substrates containing mesityl, 2,4,6‐triisopropylphenyl, and 4‐*tert*‐butylphenyl substituents (substrates **20–22**, respectively) reached full conversion and moderate product yields within 14 h, indicating that the reaction proceeds even with steric hindrance. Primary alcohol substrates with electron‐deficient fluoro (**19**), electron‐donating methoxy (**23**), sulfur in 2‐(thiophen‐3‐yl)ethan‐1‐ol (**24**), and fused aryl rings in 2‐(naphthalen‐1‐yl)ethan‐1‐ol (**25**) were all suitable as well, with high conversions and reasonable yields, confirming that branching on the alcohol carbon was not critical in this reaction. These results further reinforced the possibility of applying our photocatalyst for C—C bond activation in diverse, electron‐rich, electron‐deficient, and even sterically challenging substrates.

Finally, we explored the possibility of API synthesis by using some of the reaction products as precursors. For instance, **13** was found to selectively provide 3,4‐dimethoxybenzaldehyde in high yields. We then used 3,4‐dimethoxybenzaldehyde in the synthesis of etamivan (**26**), which is an API employed in treating chronic respiratory system diseases and barbiturate overdose. Details regarding the synthesis of **26** are summarized in the Supporting Information.

### 2.2 Cascade C—C Cleavage for Sustainable Energy and Environmental Remediation Applications

**Figure 4:**
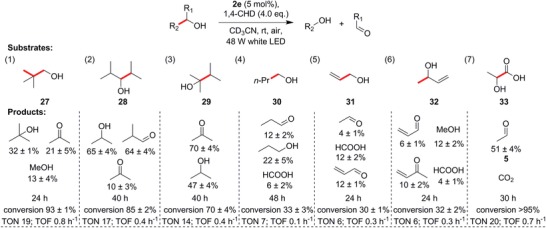
Reactivity of simple, commercially sourced alcohols. The bonds that undergo cleavage are highlighted in bold and red. Substrates **27** and **30** indicate a possibility for cascade C—C bond cleavage, whereas **31** and **32** suggest the absence of ^1^O_2_ related reactions. Substrate **33** highlights the possibility of converting biomass‐derived compounds to feedstock chemicals. The TONs and TOFs are shown below each substrate.

Apart from the substrates that generate stabilized benzyl radicals upon C—C bond cleavage, we were intent on expanding this unique reaction to readily available, unactivated, acyclic, and aliphatic alcohols that would form more reactive tertiary, secondary, or even primary radicals. Gratifyingly, substrates that led to these progressively less stable radicals proceeded (**Figure** **4**, substrates **27–30**), although the reaction times also gradually increased with concomitant reduction in conversion. Some of the products from these shorter‐chain alcohols, such as formaldehyde, methanol, propionaldehyde, and acetone, are volatile, and formaldehye is notoriously unstable. Under prolonged irradiation with our setup, these compounds could undergo side reactions such as oligomerization or would evaporate at room temperature, accounting for the diminished product yields in spite of the relatively high conversions.

Remarkably, for **30**, we observed that in the absence of the 1,4‐CHD antioxidant, one of the primary products, 1‐propanol, appeared to undergo a subsequent C—C bond cleavage in situ to generate ethanol. To exclude the possibility of the intermediacy of singlet oxygen (^1^O_2_) generated by energy transfer from **2e**, we used the allyl alcohols, **31** and **32**, as substrates (Figure 4, Entries 5 and 6). Allylic alcohols are known to react with ^1^O_2_ to produce hydroperoxides or epoxides via ene‐type reactions. However, in the presence of **2e** at low conversions, we detected mainly C—C bond cleavage products, and small amounts of carbonyl products, indicating that ^1^O_2_ was unlikely to be the predominant active species. In all the examples thus far, we observed that wherever the primary carbonyl product is stable, the yields are very high, while the combined yields from the other oxygenated product remain respectable.

In addition to substrates that generate simple alkyl radicals upon C—C bond cleavage, we investigated whether other types of radicals could be formed. We chose the racemic lactic acid (**33**), which is usually obtained by microbial fermentation of sugars, and is the monomer of the biodegradable plastic polylactic acid (PLA). Remarkably, **33** underwent C—C bond activation quantitatively in a clean reaction (Figure 4, Entry 7), yielding moderate amounts of the volatile **5** and presumably CO_2_, although the latter was not quantified. This result identified another viable type of substrates for our reaction, in which acyl radicals are formed. Furthermore, this example highlights the possibility of converting biomass‐derived lactic acid or recycling PLA back to **5** as a chemical feedstock.

**Figure 5:**
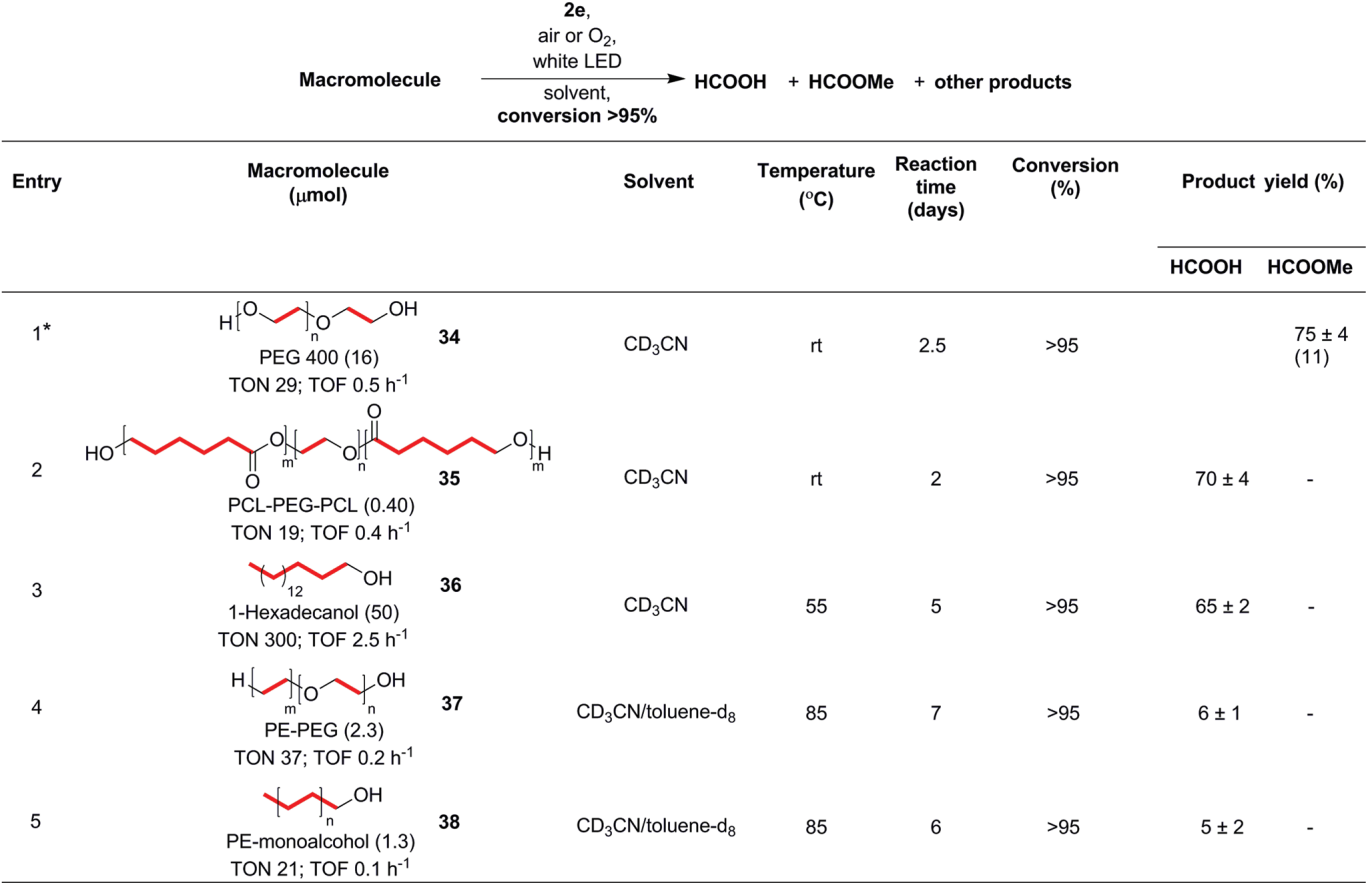
Cascade C—C bond cleavage in macromolecular hydroxyl functionalized biodegradable (**34**–**36**) and non‐biodegradable (**37** and **38**) polymers. In all the polymeric substrates, the sites of C—C bond cleavage are highlighted in red, with multiple red colored C—C bonds indicating the cascade shortening of the polymer chain. The NMR yields of the identified products are shown. The TONs and TOFs shown are calculated based on the number of C—C bonds in the substrate. *Conducted with 2 eq. of methanol relative to the monomer unit. The isolated yield of methyl formate from vacuum transfer is shown in parentheses.

The cascade C—C bond cleavage in 1‐butanol observed in the absence of 1,4‐CHD inspired us to target longer‐chain alcohols and even macromolecular substrates such as polymers (**Figure** **5**). Likewise, we conducted the reactions using the macromolecular alcohols without 1,4‐CHD so that we would not need stoichiometric additives and unnecessary by‐products would not be generated. We started with polyethylene glycol (PEG) since it dissolves readily at room temperature in acetonitrile, our reaction solvent. Accordingly, PEG 400 (**34**) underwent full conversion within 2.5 days under our optimized, ambient conditions in the absence of 1,4‐CHD to yield significant amounts of formic acid, small amounts of methyl formate, other alkyl formates, and oligomeric products. We believe that the initially generated formaldehyde may oligomerize to paraformaldehyde or was further oxidized to formic acid during the reaction, which could then have become esterified.

**Figure 6:**
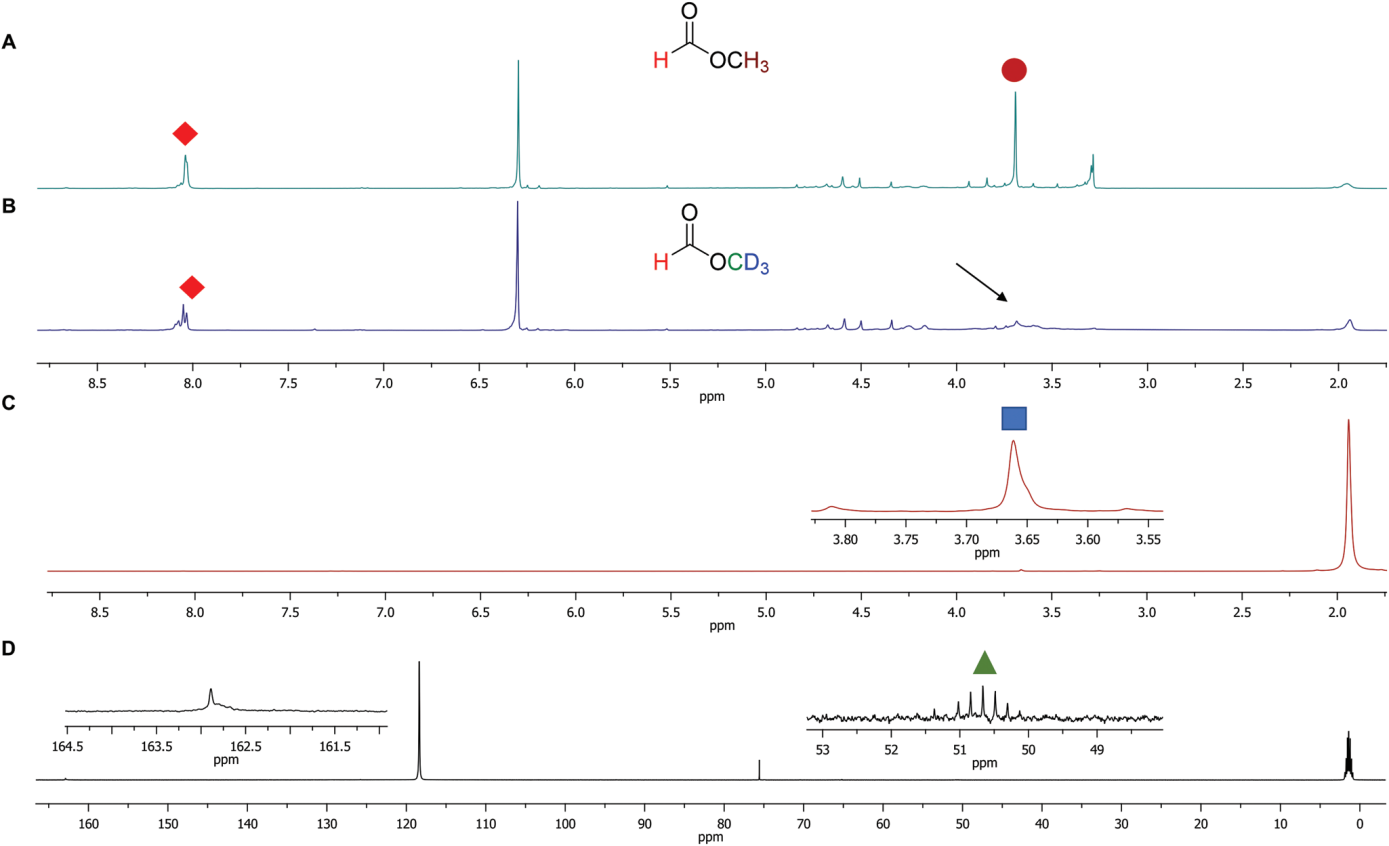
Conversion of PEG to predominantly methyl formate in the presence of MeOH or MeOD‐d_4_ under our photoredox reaction conditions. A) The ^1^H NMR spectrum of the reaction mixture with MeOH after 2.5 days of light irradiation. The B) ^1^H, C) ^2^H{^1^H}, and D) ^13^C{^1^H} NMR spectra of the reaction mixture with MeOD‐d_4_ instead of MeOH after the same irradiation time. The black arrow indicates the absence of the CH_3_ peak when MeOD‐d_4_ is used instead of MeOH. The red tilted square and maroon circle represent the formate H and CH_3_, while the blue square and green triangle correspond to the CD_3_ and a carbon originating from MeOD‐d_4_, respectively. The peaks at 6.30 ppm in the ^1^H NMR spectra originate from the 1,1,2,2‐tetrachloroethylene internal standard, while its carbons appear at 75.6 ppm in the ^13^C{^1^H} NMR spectrum. The NMR experiments were all conducted in CD_3_CN. The insets show vertically expanded sections of the spectra. The observation of methyl formate and other unstable products at lower substrate conversion is shown in Figure S4 (Supporting Information).

In a subsequent experiment, we added two equivalents (eq.) of methanol relative to the monomeric unit to trap the formaldehyde as more stable products. Notably, the ^1^H NMR data showed a much more selective reaction, with significant amounts of methyl formate as the major product (**39**, **Figure** **6**A; Figure S4, Supporting Information). To verify whether **39** originated from the condensation between methanol and formic acid by C—C bond cleavage from PEG 400, or was entirely from partial oxidation of the exogenous methanol, we repeated the experiment but with two eq. of deuterated methanol (MeOD‐d_4_) relative to the monomer unit instead. Accordingly, we observed the absence of the methyl protons of the ester but the retention of the formate proton in the ^1^H NMR spectrum (Figure 6B). Furthermore, the appearance of a peak at 3.69 ppm in the ^2^H{^1^H} NMR (Figure 6C) and a multiplet between 50 and 52 ppm but predominantly a singlet at 162.9 ppm in the ^13^C{^1^H} spectrum (Figure 6D) suggested that **40** was indeed HC(O)OCD_3_ formed from both MeOD‐d_4_ and formic acid derived from PEG 400. This promising result allows us to recycle PEG into valuable precursors such as esters and carboxylic acids.

Following our success with **34**, we then applied this cascade C—C bond cleavage and oxygenation to the poly‐ε‐caprolactone‐*block*‐polyethyleneglycol‐*block*‐poly‐ε‐caprolactone (PCL–PEG–PCL) triblock co‐polymer (**35**). The reaction proceeded smoothly with complete polymer transformation within 2 days under ambient conditions, with formic acid as the major product (Figure 5, Entry 2). Analogously, in a following experiment, the addition of methanol enabled us to predominantly obtain **39** as the major product. With these two polymer examples in hand, we proceeded to screen PLA and polyvinyl alcohol (PVA) for reactivity. However, both polymers showed limited solubility in acetonitrile. Moreover, since they are biodegradable polymers, we chose to focus our attention on the environmentally detrimental, non‐biodegradable polymers commonly found in microplastic pollutants instead.

Encouraged by the respectable product yields from recycling the biodegradable **34** and its block‐copolymer **35**, we then examined whether non‐biodegradable analogs could be substrates too. Plastics based on the polyethylene backbone, such as polypropylene and polystyrene, have unfortunately proliferated and become recalcitrant wastes that pollute the oceans.^64^, ^65^, ^66^ As a model substrate for polyethylene, we selected a long‐chain alcohol, 1‐hexadecanol (**36**). Due to its poor solubility in acetonitrile at room temperature, **36** had to be heated to 55 °C during the reaction to allow full dissolution. Gratifyingly, after 5 days of irradiation with visible light and **2e**, more than 95% of **36** reacted to generate good yields of formic acid as one of the major products. Control experiments conducted in the dark or in the absence of **2e** at 55 °C showed that **36** did not react at all over the same time period.

After establishing the reaction conditions for this polyethylene model, we targeted the polyethylene‐*block*‐polyethylene glycol (PE‐PEG, **37**) co‐polymer next. Although the reaction had to be conducted at 85 °C in a mixture of acetonitrile and toluene to fully dissolve the substrate, remarkably, full conversion of both the PE and PEG units was observed in the ^1^H NMR spectrum of the reaction mixture after ≈7 days. The products included formic acid and shorter‐chain alkyl formates. We expect that much of the formic acid (boiling point of 101 °C) had evaporated at 85 °C under the reaction conditions, although sealed, high‐pressure reactors can be used to retain volatile products. Likewise, the control experiments confirmed that **37** was stable at 85 °C in the dark or in the absence of **2e** under otherwise identical reaction conditions.

Finally, we targeted polyethylene itself, as a representative of non‐biodegradable polymers derived from the polyethylene backbone. We anticipate that the photocatalytic C—C cleavage of other polyethylene derivatives (e.g., polystyrene) should be even more facile since the intermediate radicals would be stabilized at the branch point and **2e** can tolerate a broad range of substituents. We used polyethylene monoalcohol (**38**) under similar conditions as **37**. Remarkably, after 6 days, full substrate conversion was observed, in contrast to the previously reported example for photoreforming of plastics, where a conversion of up to only 50% was reported after 18 days.^41^, ^52^ Furthermore, our system does not require alkaline pretreatment to initiate partial hydrolysis of the polymers to hasten the reaction.^41^, ^52^ We expect the reaction to proceed via cascade C—C bond cleavage similar to the other macromolecular substrates that we tested, and a plausible reaction mechanism based on the observed products and our prior studies^56^, ^57^ is depicted in Figure S5 (Supporting Information).

Similar to the other examined polymers, formic acid and alkyl formates were identified as some of the products. In addition, CO_2_ in the headspace was also detected by gas chromatography (estimated amount of 62% of the theoretical yield), which could account for the diminished product yields despite the high conversions. In contrast, when reactions of **36** were conducted at lower temperatures of 55 °C, moderate yields of formic acid were obtained, indicating that the over‐oxidation of formic acid to CO_2_ could be a result of the elevated reaction temperatures that we needed to solubilize the polymers. On the other hand, the control experiments with **38** conducted in the absence of light or without **2e** at the same elevated temperatures showed no observable loss of **38**. Since formic acid can be directly fed into fuel cells or can be used as a liquid storage for H_2_,^67^, ^68^, ^69^ while alkyl formates are solvents, refrigerants, and platform chemicals,^70^ our photocatalytic approach to repurpose non‐biodegradable plastics into useful, small molecule fuels and feedstocks can be a prospective and scalable oxidative half‐reaction in an integrated AP system (Figure 1B).

## 3 Conclusions

In conclusion, we have demonstrated the diverse applicability of a selective aliphatic C—C single bond cleavage under visible light irradiation and mild reaction conditions for both organic synthesis and the environmental remediation of plastics performed in organic solvents. Our approach is suitable for a wide range of alcohol substrates. The aerobic conditions allowed for oxygenation of the transient alkyl radicals upon C—C bond cleavage, which led to new alcohol products that could undergo controlled, cascade C—C bond activation. We applied this pioneering strategy to several hydroxyl‐terminated polymers, including the non‐biodegradable polyethylene and its block copolymer, and obtained formates and formic acid as products. Although this study has demonstrated the possibility of cascade C—C bond cleavage in small molecule model substrates and commercially sourced polymers, they are nonetheless not actual plastic consumer products. Our current efforts are directed toward applying this approach to a broader range of polymers, either by first preprocessing them to be soluble in acetonitrile or creating a heterogeneous analog of our vanadium catalyst that can operate in other solvents or in (photo)‐electrochemical systems integrated for solar fuels' production. Thus, this work highlights the utility of photocatalytic C—C activation as a synthetic tool for unusual transformations of complex alcohols and the repurposing of persistent plastic pollutants into fuels and chemical feedstocks using renewable energy. Moreover, we offer a conceptual advance of unifying photoredox catalysis with valorizing nominal waste materials and pollutants to create more value‐added, but still potentially scalable alternative reactions for AP, systems.

## Conflict of Interest

A patent application covering part of this work on lignin model compounds and biomass lignin was filed (11201705500Q PCT‐SG) by NTUitive, the innovation and enterprise company of Nanyang Technological University, Singapore, naming S.G., M.Đ., and H.S.S. as the inventors.

## Supporting information

Supporting Information
