# Supplementary material for: Visible Light–Driven Cascade Carbon–Carbon Bond Scission for Organic Transformations and Plastics Recycling
Source: Adv Sci (Weinh). 2019 Oct 24;6(24):1902020. doi: 10.1002/advs.201902020 (PMC6918108; doi:10.1002/advs.201902020)
Supplement: Supplementary file 1 — Supporting Information [file ADVS-6-1902020-s001.pdf]

Copyright WILEY-VCH Verlag GmbH & Co. KGaA, 69469 Weinheim, Germany, 2019.

Supporting Information

for *Adv. Sci.,* DOI: 10.1002/advs.201902020

Visible Light–Driven Cascade Carbon–Carbon Bond Scission for Organic Transformations and Plastics Recycling

*Sarifuddin Gazi, Miloš Đokić, Kek Foo Chin, Pei Rou Ng, and Han Sen Soo**

Copyright WILEY-VCH Verlag GmbH & Co. KGaA, 69469 Weinheim, Germany, 2018.

Supporting Information

Visible Light Driven Cascade Carbon-Carbon Bond Scission for Organic Transformations and Plastics Recycling

*Sarifuddin Gazi, Miloš Đokić, Kek Foo Chin, Pei Rou Ng, and Han Sen Soo**

Table of Contents

Experimental Procedures

General information 3

General procedure for the synthesis of ligands and complexes 3

Figure S1 Synthetic procedure for the preparation of ligands (1a-f) and vanadium complexes (**2a-f**). 3

Procedures for the synthesis of additives and substrates 3

Procedures for the transformation of a reaction product E0 into an API etamivan 9

Procedures for the optimization of the photocatalytic reactions 10

Table S1 Full screening conditions of different HAT additives to maximize the primary C-C bond cleavage products. 11

Table S2 Effects of air or O_2 on the substrate conversion and product distribution. 12

Table S3 Effects of various catalyst loadings on substrate conversion and product distribution 12

Table S4 Effects of different catalysts on substrate conversion and product distribution 13

Table S5 Effects of alternative oxidants on substrate conversion and product distribution 13

Figure S2 Optimization of conditions for photocatalytic C-C bond cleavage in 3 14

Procedures for the mechanistic screening of substrates 14

Figure S3 Mechanistic screening of substrates proposed by Glorius *et al.* 15

Procedures for the photocatalytic reactions with small and macromolecular alcohols 15

Figure S4 The ^{1}H NMR spectra of 34 before (top) and after 22 h (bottom) of visible light irradiation ...16

Figure S5 Proposed reaction mechanism for cascade C-C bond cleavage in polyethylene monoalcohol 17

Calculations for the amount of CO_2 produced during the PE-monoalcohol (**38**) photolysis 17

References 18

Identification of reaction products 19

Spectral data 23

General information

The vanadium complexes and some ligand precursors used in this study were prepared under a N_2 atmosphere employing standard Schlenk techniques in accordance with our previously reported procedure[1]. The remaining synthetic procedures were conducted under aerobic conditions. The chemicals used in the experiments were purchased from Sigma-Aldrich and were used as received. Deuterated solvents were purchased from Cambridge Isotope Laboratories and were also used as received. The ^{1}H, ^{13}C, ^{19}F, and ^{11}B NMR spectroscopic data were obtained using Bruker AVANCE spectrometers operating at 300, 400, and 500 MHz. The resulting ^{1}H and ^{13}C NMR chemical shifts (δ reported in ppm) were referenced according to the residual solvent signal(s) ($CDCl_3$ = 7.26 ppm for ^{1}H and 77.2 ppm for ^{13}C; CD_3CN = 1.94 ppm for ^{1}H and 118.3 ppm for ^{13}C; MeOD-d_4 = 3.31 ppm for ^{1}H and 49.2 ppm for ^{13}C; DMSO-d_6 = 2.50 ppm for ^{1}H and 39.52 ppm for ^{13}C). The ^{11}B NMR chemical shifts were referenced to an external standard BF_3 (δ = 0.0 ppm). High-resolution electrospray ionization mass spectra (HR-ESIMS) were obtained using a Waters Q-TOF Premier mass spectrometer.

General procedure for the synthesis of ligands and complexes

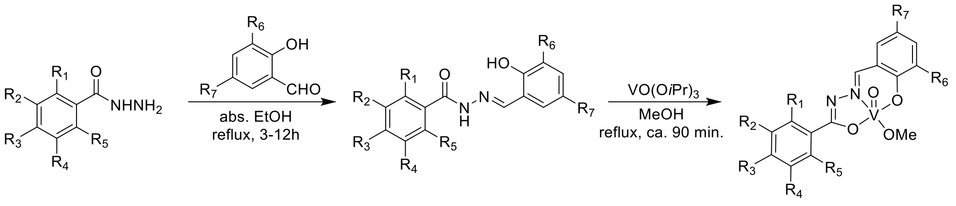

1a-f **2a-f**

1a: R_1-R_7=H
1b: R_1,R_2,R_4,R_5,R_6,R_7=H; R_3=F;
1c: R_1,R_2,R_4,R_5,R_7=H; R_3=F; R_6=*tert*-Bu
1d: R_1-R_5=F; R_6,R_7=H
1e: R_1-R_5=F; R_6=H; $R_7=NO_2$
1f: R_1-R_5=F; $R_6,R_7=NO_2$

2a: R_1-R_7=H
2b: R_1,R_2,R_4,R_5,R_6,R_7=H; R_3=F;
2c: R_1,R_2,R_4,R_5,R_7=H; R_3=F; R_6=*tert*-Bu
2d: R_1-R_5=F; R_6,R_7=H
2e: R_1-R_5=F; R_6=H; $R_7=NO_2$
2f: R_1-R_5=F; $R_6,R_7=NO_2$

Figure S1 Synthetic procedure for the preparation of ligands (**1a**-**f**) and vanadium complexes (**2a**-**f**). The vanadium oxo complexes (**2a**-**2f**) used as photoredox catalysts in this study have been synthesized by following the procedure reported in our earlier work.[1] Details regarding the synthesis and characterization of the catalysts and the corresponding ligands can be found at: http://pubs.acs.org/doi/suppl/10.1021/acscatal.7b01036.

Procedures for the synthesis of additives and substrates

Synthesis of *tert*-butyl phenylcarbamate

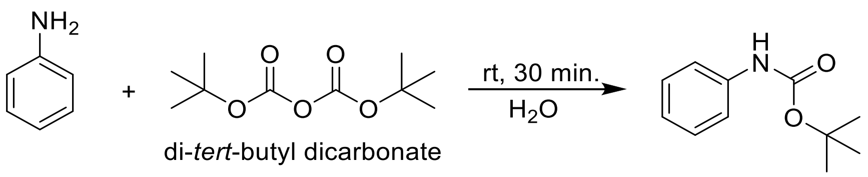

Compound *tert*-butyl phenylcarbamate (*N*-Boc-aniline) was prepared in accordance with a reported procedure.[2] To a stirring solution of aniline (0.24 g, 2.5 mmol) in 2.5 mL of water was added di-*tert*-butyl dicarbonate (0.60 g, 2.8 mmol). After stirring for ca. 30 min. at room temperature, white solids started to form. The solids were collected by decanting off the supernatant water, washed with excess water, and dried under reduced pressure to obtain the desired compound as a white solid (0.44 g, 95%). ^{1}H NMR (CD_3CN, 400 MHz): δ = 1.49 (s, 9 H), 6.99-7.03 (m, 1 H), 7.25-7.30 (m, 2 H), 7.40 (dd, J = 0.9, 8.6 Hz, 2 H), 7.48 (bs, 1 H) ppm. $^{13}C\{^{1}H\}$ NMR (CD_3CN, 100 MHz): δ = 28.0, 79.9, 118.9, 123.0, 129.2, 139.7, 153.5 ppm.

Synthesis of 1-phenylpropan-2-ol (3)

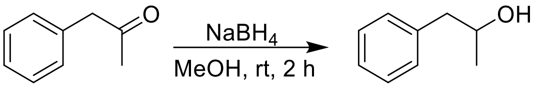

3

To a solution of 1-phenylpropan-2-one (0.68 g, 5.1 mmol) in methanol (6 mL) was added sodium borohydride (0.19 g, 5.1 mmol) in portions. After stirring at room temperature for ca. 2 h, the unreacted $NaBH_4$ was quenched by the addition of water (10 mL) and the mixture was extracted with ethyl acetate (EtOAc, 3 x 20 mL). The organic fractions were collected, dried over anhydrous $NaSO_4$, and concentrated under reduced pressure. Removal of the solvent in vacuum yielded **3** as a yellow liquid (0.57 g, yield 84%). ^{1}H NMR (CD_3CN, 400 MHz): δ = 1.11 (d, J = 6.2 Hz, 3 H), 2.67-2.70 (m, 2 H), 3.91 (dd, J = 3.9, 6.1 Hz, 1 H), 7.19-7.31 (m, 5 H) ppm. ^{13}C{^{1}H} NMR (CD_3CN, 100 MHz): δ = 23.4, 46.3, 69.2, 126.9, 129.1, 130.4, 140.6 ppm.

General procedure for the synthesis of substrates 7-10, **15**, and **16**

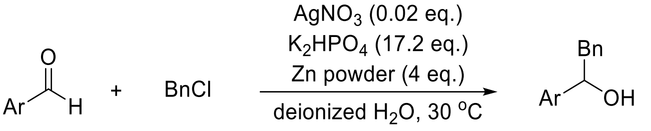

C1 **C2** **7-10, 15, 16**

The substrates **7-10**, **15**, and **16** were synthesized by following a previously published general procedure.[3] To a clear solution of $AgNO_3$ (0.020 eq.) and K_2HPO_4 (17.2 eq.) in deionized water at 30 °C, **C1** (1.0 eq.) and **C2** (1.5 eq.) were added together. Under vigorous stirring, zinc powder (4.0 eq.) was added portion-wise over 10 minutes. After the reaction was completed (progress was monitored by thin layer chromatography, TLC), the mixture was acidified with 2.0 M $HCl_{(aq)}$ and extracted with EtOAc thrice. The combined organic layers were washed with brine, dried over anhydrous Na_2SO_4, and concentrated under reduced pressure to obtain the crude product. Subsequent purification was performed by flash column chromatography on silica gel with hexane and EtOAc as eluents. Yields of the pure products obtained ranged from 45 to 72%.

Compound 1,2-diphenylethan-1-ol (7)

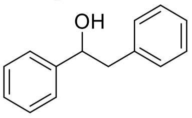

Compound **7** was prepared in accordance with the procedure described above using benzaldehyde as **C1**. The product was isolated as a white solid (0.287 g, yield 72%). ^{1}H NMR (CD_3CN, 300 MHz): δ = 2.97 (d, J = 6.8 Hz, 2 H), 3.24 (d, J = 4.3 Hz, 1 H), 4.82-4.88 (m, 1 H), 7.16-7.35 (m, 10 H) ppm. ^{13}C{^{1}H} NMR ($CDCl_3$, 100 MHz): δ = 46.0, 75.3, 125.9, 126.6, 127.6, 128.4, 128.5, 129.5, 138.0, 143.8 ppm. The NMR spectroscopic data match the reported values.[4]

Compound 1-(4-fluorophenyl)-2-phenylethan-1-ol (8)

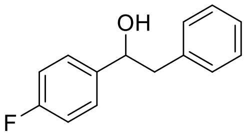

Compound **8** was prepared following the described procedure using 4-fluorobenzaldehyde as **C1**. The product was isolated as a pale yellow solid (0.248 g, yield 57%). ^{1}H NMR (CD_3CN, 300 MHz): δ = 2.94-2.96 (m, 2 H), 3.30 (d, J = 4.1 Hz, 1 H), 4.85 (dd, J = 6.7, 10.5 Hz, 1 H), 7.01-7.34 (m, 9 H) ppm. ^{13}C{^{1}H} NMR ($CDCl_3$, 100 MHz): δ = 46.2, 74.8, 115.3 (d, J = 21.2 Hz), 126.8, 127.7 (d, J = 8.0 Hz), 128.6, 129.6, 137.8, 139.6 (d, J = 3.0 Hz), 162.3 (d, J = 244 Hz) ppm. ^{19}F{^{1}H} NMR ($CDCl_3$, 376.5 MHz): δ = -115.0 ppm. The NMR spectroscopic data match the reported values.[4]

Compound 1-(4-bromophenyl)-2-phenylethan-1-ol (9)

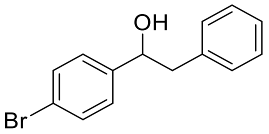

Compound **9** was prepared following the procedure described earlier using 4-bromobenzaldeyhde as **C1**. The product was isolated as a pale yellow solid (0.395 g, yield 71%). ^{1}H NMR ($CDCl_3$, 300 MHz): δ = 2.24 (s, 1 H), 2.92-3.02 (m, 2 H), 4.83 (t, J = 6.4 Hz, 1 H), 7.16-7.21 (m, 4 H), 7.24-7.36 (m, 3 H), 7.46-7.48 (m, 2 H) ppm. ^{13}C{^{1}H} NMR ($CDCl_3$, 100 MHz): δ = 45.8, 74.5, 121.2, 126.6, 127.6, 128.4, 129.4, 131.3, 137.4, 142.6 ppm. The NMR spectroscopic data match the reported values.[4]

Compound 2-phenyl-1-(4-(trifluoromethyl)phenyl)ethan-1-ol (10)

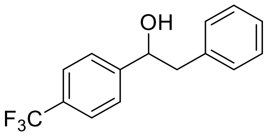

Compound **10** was prepared according to the previously described procedure using 4-(trifluoromethyl)benzaldehyde as **C1**. The final product was isolated as a white solid (0.354 g, yield 67%). ^{1}H NMR ($CDCl_3$, 400 MHz): δ = 2.97 (d, J = 6.7 Hz, 2 H), 3.45 (d, J = 4.2 Hz, 1 H), 4.95 (m, 1 H), 7.16-7.28 (m, 5 H), 7.49 (d, J = 8.4 Hz, 2 H), 7.62 (d, J = 8.2 Hz, 2 H) ppm. ^{13}C{^{1}H} NMR ($CDCl_3$, 100 MHz): δ = 46.1, 74.2, 122.8, 125.3 (q, J = 3.7 Hz), 126.2, 126.9, 128.6, 129.5, 129.9, 137.3, 147.6 ppm. ^{19}F{^{1}H} NMR ($CDCl_3$, 376.5 MHz): δ = -62.4 ppm. The NMR spectroscopic data match the reported values.[5]

Compound 2-phenyl-1-(*p*-tolyl)ethan-1-ol (11)

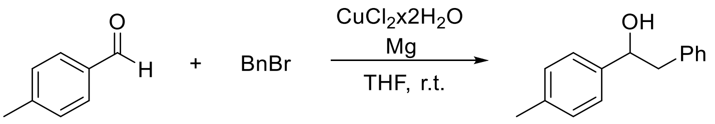

11

Compound **11** was prepared by adopting a reported procedure.[6] To a stirring mixture of 4-methylbenzaldehyde, benzyl bromide, and $CuCl_2{\cdot}2H_2O$ in THF were added Mg turnings. The mixture was stirred at ambient temperature and monitored by TLC. After the reaction was completed, the reaction mixture was then treated successively with 20 mL of H_2O and EtOAc, stirred for 10 minutes more, and then filtered. The filtrate was treated with 50 mL of 2% $HCl_{(aq)}$ to dissolve a small number of suspended particles. The organic layer was separated, and the aqueous layer was extracted with EtOAc thrice (20 mL x 3). The combined organic layers were washed with H_2O, brine, and then dried over anhydrous Na_2SO_4. Removal of the solvent under reduced pressure yielded the crude product. Subsequent purification was performed by flash column chromatography on silica gel using *n*-hexane/EtOAc as the eluent. The pure product was isolated as a colourless oil (0.162 g, yield 38%). ^{1}H NMR (CD_3CN, 300 MHz): δ = 2.30 (s, 3 H), 2.94 (d, J = 6.8 Hz, 2 H), 3.17 (d, J = 4.3 Hz, 1 H), 4.80 (m, 1 H), 7.04-7.32 (m, 9 H) ppm. ^{13}C{^{1}H} NMR ($CDCl_3$, 100 MHz): δ = 21.2, 46.1, 75.3, 126.0, 126.6, 128.6, 129.2, 129.6, 137.3, 138.3, 141.0 ppm. The NMR spectroscopic data match the reported values.[4]

Procedure for the synthesis of substrates 12, **13**, and **22**

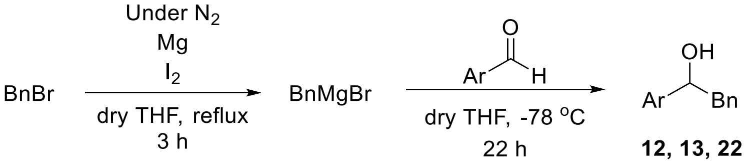

Substrates **12**, **13**, and **22** were prepared by using the general procedure shown above. Under a N_2 atmosphere, to an oven-dried two-neck round bottom flask fitted with a reflux condenser, Mg (1.2 eq.) was added, followed by the addition of anhydrous THF and I_2 (1-2 crystals). The mixture was stirred at reflux for five minutes and then benzyl bromide or 4-*tert*-butylbenzyl bromide (1 eq.) was added dropwise. The reaction mixture was warmed up until it began to reflux, and discolouration was observed. The addition of benzyl bromide was maintained at that rate. After the complete addition, the reaction was maintained at reflux for three hours. The reaction mixture was

subsequently cooled down to room temperature, and then further down to -78 °C using an acetone-dry ice bath. The mixture was then stirred at -78 °C for 10 minutes. Subsequently, the corresponding aryl aldehyde (1.0 eq. diluted with anhydrous THF) was added dropwise into the reaction mixture at -78 °C. After complete addition, the acetone-dry ice bath was removed, and the reaction mixture gradually warmed up to room temperature. The progress of the reaction was monitored by TLC. After the reaction was completed, saturated aqueous ammonium chloride solution was added dropwise into the reaction mixture. The aqueous layer was then extracted with EtOAc thrice. The combined organic layers were washed with brine, dried over anhydrous Na_2SO_4, and concentrated under reduced pressure to yield the crude product. Subsequent purification was performed by flash column chromatography on silica gel with solutions of hexane and EtOAc as the eluents.

Compound 1-(2,6-dimethylphenyl)-2-phenylethan-1-ol (12)

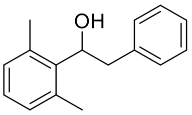

Compound **12** was synthesized by following the procedure described above. The pure product was isolated as a colourless oil (0.455 g, yield 40%). ^{1}H NMR (CD_3CN, 300 MHz): δ = 2.38 (s, 6 H), 3.00 (dd, J = 6.1, 13.3 Hz, 1 H), 3.17 (dd, J = 8.7, 12.9 Hz, 2 H), 5.26-5.32 (m, 1 H), 6.95-7.05 (m, 3 H), 7.18-7.28 (m, 5 H) ppm. ^{13}C{^{1}H} NMR ($CDCl_3$, 100 MHz): δ = 20.8, 42.2, 72.8, 126.5, 127.1, 128.5, 129.4, 129.5, 136.1, 138.6, 138.7 ppm. HRMS (ESI+, *m/z*) calculated for $C_{16}H_{19}O$ $[M+H]^+$ *m/z* = 227.1436, found 227. 1427.

Compound 1-(3,4-dimethoxyphenyl)-2-phenylethan-1-ol (13)

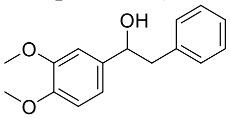

Compound **13** was synthesized by following the procedure described above with benzyl bromide and 3,4-dimethoxybenzaldehyde as precursors. The product was isolated as a white solid (1.55 g, yield 60%). ^{1}H NMR (CD_3CN, 300 MHz): δ = 2.96 (d, 2 H, J = 5.4 Hz), 3.23 (d, 1 H, J = 4.2 Hz), 3.76 (s, 3 H), 3.77 (s, 3 H), 4.76-4.82 (m, 1 H), 6.83 (s, 2 H), 6.90 (s, 1 H), 7.18-7.28 (m, 5 H) ppm. ^{13}C{1H} NMR (CD_3CN, 75.47 MHz): δ = 46.5, 56.2, 56.3, 75.4, 110.9, 112.2, 119.1, 126.9, 129.0, 130.5, 138.6, 140.1, 149.2, 149.9 ppm. HRMS (ESI+, *m/z*) calculated for $C_{16}H_{19}O_3$ $[M+H]^+$ *m/z* = 259.1334, found 259.1335.

Synthesis of compound 1-(4-nitrophenyl)-2-phenylethan-1-ol (14)

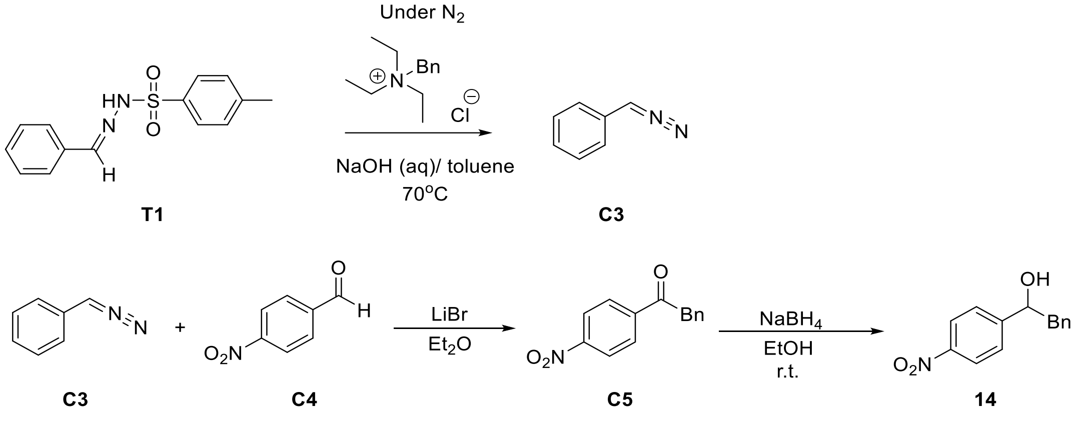

Compound **14** was prepared according to several reported procedures.[7-9] Precursor compound **T1** was prepared in accordance with a reported procedure.[10] Precursor **T1** (2.9 mmol, 1.0 eq.) was dissolved in 20 mL toluene and was slowly added to a solution of benzyltriethylammonium chloride (0.44 mmol, 0.15 eq.) in 8.2 mL of aqueous NaOH (14% w/w) under a N_2 atmosphere. The reaction mixture was heated to 70 °C and maintained at that temperature with stirring for two hours. A progressive red colouration of the solution was observed. After cooling the mixture to room temperature, the organic layer was quickly separated from the aqueous layer, washed twice with 10 mL of water, and dried over anhydrous Na_2SO_4. Removal of toluene under reduced pressure yielded intermediate **C3**, which was used in the next step without further purification.

To a stirred mixture of **C4** (2.92 mmol, 1.0 eq.) and LiBr (29.2 mmol, 10 eq.) in 20 mL of Et_2O cooled in an ice-salt bath (-5 °C) was added a diluted solution of **C3** (2.92 mmol, 1.0 eq.) in 20 mL toluene through a dropping funnel (note: the reaction flask was protected from light with Al-foil). The mixture was slowly warmed up to

room temperature and stirred overnight. Subsequently, deionized water was added, and the organic layer was separated and dried over anhydrous $MgSO_4$. The solvent was then removed under reduced pressure, yielding the crude product. Purification of the crude product was performed by flash column chromatography on silica gel using 6:1 *n*-hexane/EtOAc as the eluent to afford **C5** as colourless oil (0.32 g, 45% yield).

To a solution of **C5** (1.3 mmol) in ethanol (EtOH, 6 mL) was slowly added $NaBH_4$ (2.6 mmol, 2.0 eq.). The resulting clear solution was stirred at room temperature and monitored by TLC. After the reaction was completed, saturated aqueous ammonium chloride was added dropwise into the reaction mixture. The aqueous layer was then extracted with EtOAc thrice. The combined organic layers were washed with brine and concentrated under reduced pressure to yield the crude product. Purification of the crude material was performed by flash column chromatography on silica gel using 6:1 *n*-hexane/EtOAc as the eluent to afford **14** as a white solid (0.31 g, 97% yield). ^{1}H NMR (CD_3CN, 300 MHz): δ = 2.98 (d, J = 6.8 Hz, 2 H), 3.55 (d, J = 4.2 Hz, 1 H), 5.00 (m, 1 H), 7.15-7.28 (m, 5 H), 7.52 (d, J = 8.5 Hz, 2 H), 8.13-8.16 (m, 2 H) ppm. ^{13}C{^{1}H} NMR ($CDCl_3$, 100 MHz): δ = 46.0, 74.3, 123.6, 126.6, 127.0, 128.7, 129.5, 136.7, 147.3, 150.9 ppm. The NMR spectroscopic data match the reported values.[11]

Compound 1-(perfluorophenyl)-2-phenylethan-1-ol (15)

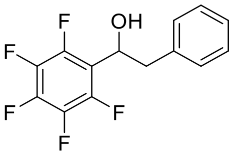

Compound **15** was prepared following the earlier described procedure using 2,3,4,5,6-pentafluorobenzaldehyde as **C1**. The product was isolated as a white solid (0.342 g, yield 59%).^{1}H NMR (CD_3CN, 300 MHz): δ = 3.08 (dd, J = 7.7, 13.3 Hz, 1 H), 3.28 (dd, J = 7.4, 13.3 Hz, 1 H), 3.84 (d, J = 5.4 Hz, 1 H), 5.22 (dd, J = 7.4, 12.7 Hz, 1 H), 7.15-7.28 (m, 5 H) ppm. ^{13}C{^{1}H} NMR ($CDCl_3$, 100 MHz): δ = 43.1, 67.4, 116.2 (t, J = 13.2 Hz), 127.2, 128.8, 129.1, 136.3, 138.7 (m), 139.3 (m), 141.7 (m), 143.5 (m), 146.0 (m) ppm. ^{19}F{^{1}H} (CD_3CN, 282.4 MHz): δ = -165.1-(-164.9) (m, 2 F), -158.3 (tt, J = 1.0, 20.1 Hz, 1 F) -144.7-(-144.6) (m, 2 F) ppm. The NMR spectroscopic data match the reported values.[12]

Compound 2-phenyl-1-(4-(4,4,5,5-tetramethyl-1,3,2-dioxaborolan-2-yl)phenyl)ethan-1-ol (16)

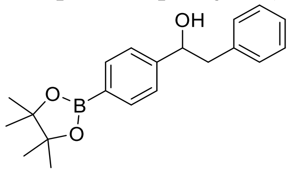

Compound **16** was prepared according to the procedure described above by using 4-(4,4,5,5-tetramethyl-1,3,2-dioxaborolan-2-yl)benzaldehyde as **C1**. The product was isolated as a white solid (0.147 g, yield 45%). ^{1}H NMR (CD_3CN, 300 MHz): δ = 1.32 (s, 12 H), 2.95 (d, J = 6.7 Hz, 2 H), 3.30 (d, J = 4.3 Hz, 1 H), 4.86 (m, 1 H), 7.15-7.25 (m, 5 H), 7.33 (d, J = 7.8 Hz, 2 H), 7.65 (d, J = 7.8 Hz, 2 H) ppm. ^{13}C{^{1}H} NMR (CD_3CN, 100 MHz): δ = 24.8, 46.0, 75.3, 83.8, 125.2, 126.6, 128.5, 129.5, 134.9, 137.9, 146.9 ppm. ^{11}B NMR (CD_3CN, 128 MHz): δ = 36.2 ppm. HRMS (ESI+, *m/z*) calculated for $C_{20}H_{26}BO_3$ $[M+H]^+$ *m/z* = 325.1975, found 325. 1973.

Synthesis of substrates 20 and 21

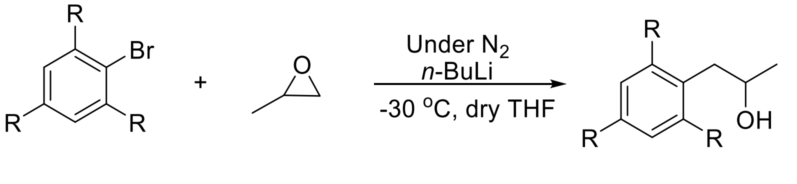

20: R = Me
21: R = *i*Pr

Compounds 1-mesitylpropan-2-ol (**20**) and 1-(2,4,6-triisopropylphenyl)propan-2-ol (**21**) were prepared by adapting a reported procedure.[13] To a stirred solution of 2-bromo-1,3,5-trimethylbenzene or 2-bromo-1,3,5-triisopropylbenzene (2.0 mmol) in anhydrous tetrahydrofuran (THF, 10 mL) at -30 °C was added *n*-butyllithium (2.2 mmol). The solution was stirred for 30 min at -30 °C, and then propylene oxide (2.0 mmol) was added. The solution was then allowed to warm up to room temperature and was stirred for an additional hour. The reaction

was subsequently quenched by the addition of saturated ammonium chloride (10 mL), and then diethyl ether (30 mL) was added. The combined organic layers were washed with water and dried with anhydrous $MgSO_4$. Finally, the solvent was removed under reduced pressure to yield the crude products, which were purified by flash column chromatography on silica (eluent: petroleum ether/EtOAc 2:1) to give the pure **20** or **21** (yield of 55 and 75%, respectively).

Compound 1-mesitylpropan-2-ol (20)

Compound **20** was prepared by following the procedure described above with 3-bromo-1,3,5-trimethylbenzene as a precursor. After the purification, **20** was isolated as a colourless liquid (0.195 g, yield 55%). ^{1}H NMR ($CDCl_3$, 300 MHz): δ = 1.30 (d, J = 6.3 Hz, 3 H), 1.60 (s, 1 H), 2.27 (s, 3 H), 2.33 (s, 6 H), 2.72-2.88 (m, 2 H), 4.01-4.07 (m, 1 H), 6.87 (s, 2 H) ppm. ^{13}C{^{1}H} NMR ($CDCl_3$, 75.47 MHz): δ = 20.4, 20.7, 23.2, 38.7, 67.8, 129.1, 132.3, 135.6, 137.0 ppm. HRMS (ESI+, *m/z*) calculated for $C_{12}H_{19}O$ $[M+H]^+$ *m/z* = 179.1436, found 179. 1435.

Compound 1-(2,4,6-triisopropylphenyl)propan-2-ol (21)

Compound **21** was prepared by following the procedure described above with 3-bromo-1,3,5-triisopropylbenzene as a precursor. After the purification, **21** was isolated as a colourless oil (0.391 g, yield 75%). ^{1}H NMR ($CDCl_3$, 500 MHz): δ = 1.20-1.29 (m, 18 H), 1.33 (d, J = 6.1 Hz, 3 H), 1.59 (s, 1 H), 2.83-2.93 (m, 3 H), 3.27-3.32 (m, 2 H), 3.95-3.99 (m, 1 H), 7.02 (s, 2 H) ppm. ^{13}C{^{1}H} NMR ($CDCl_3$, 126 MHz): δ = 23.0, 23.9, 24.0, 24.2, 24.4, 29.3, 34.1, 36.6, 69.1, 121.1, 129.1, 146.9, 147.7 ppm. HRMS (ESI+, *m/z*) calculated for $C_{18}H_{31}O$ $[M+H]^+$ *m/z* = 263.2375, found 263. 2367.

Compound 2-(4-(tert-butyl)phenyl)-1-(3,4-dimethoxyphenyl)ethan-1-ol (22)

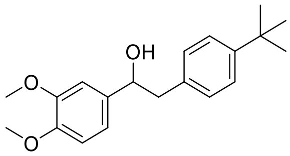

Compound **22** was synthesized by following the procedure described above with 4-*tert*-butylbenzylbromide and 3,4-dimethoxybenzaldehyde as precursors. The product was isolated as a pale yellow solid (1.55 g, yield 55%). ^{1}H NMR (CD_3CN, 300 MHz): δ = 1.29 (s, 9 H), 2.92 (d, J = 6.6 Hz, 2 H), 3.74 (s, 3 H), 3.77 (s, 3 H), 4.77 (t, 1 H, J = 6.6 Hz), 6.86 (s, 3 H), 7.11 (d, J = 8.1 Hz, 2 H,, 7.30 (d, J = 8.1 Hz, 2 H) ppm. ^{13}C{1H} NMR (CD_3CN, 75.47 MHz): δ = 31.6, 34.9, 45.9, 56.2, 56.3, 75.4, 110.9, 112.3, 119.0, 125.9, 130.2, 137.1, 138.8 149.2, 149.7, 149.8 ppm. HRMS (ESI+, *m/z*) calculated for $C_{20}H_{27}O_3$ $[M+H]^+$ *m/z* = 315.1960, found 315.1963.

Substrates **17**-**19**, **23**-**25** and **27**-**38** are commercially available and have been used as received without any further purification.

Procedures for the transformation of a reaction product E0 into an API etamivan

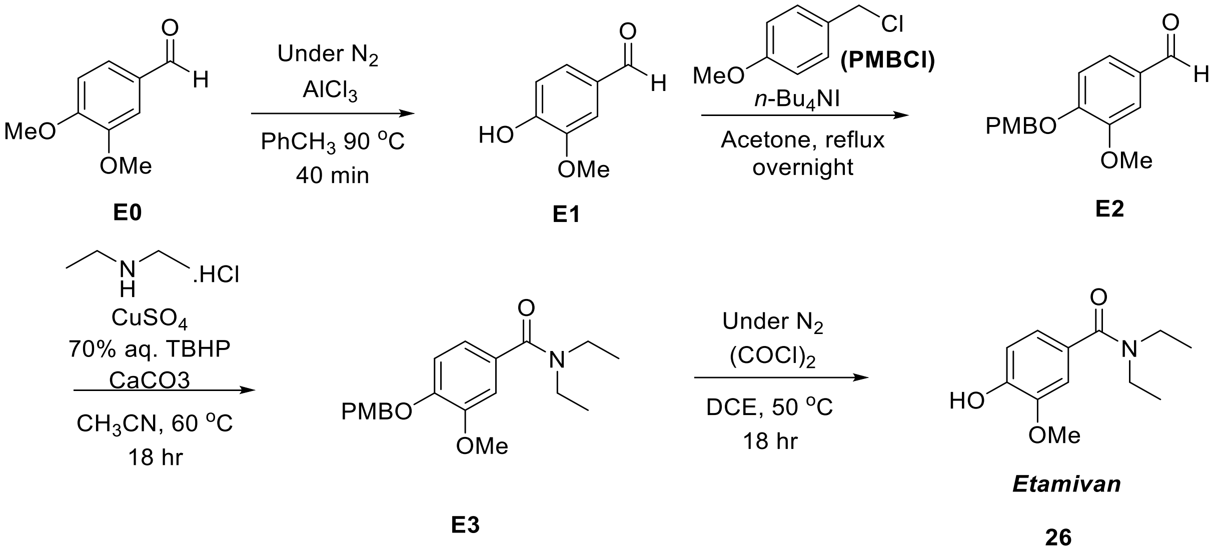

Compound 4-hydroxy-3-methoxybenzaldehyde (**E1**) was prepared from the C-C cleavage product **E0** by adopting a reported procedure.[14] Under a N_2 atmosphere, to an oven dried Schlenk flask, **E0** (16.6 mg, 0.10 mmol) was added, followed by anhydrous toluene (1 mL). To this solution, $AlCl_3$ (53.3 mg, 0.40 mmol) was added in one portion at room temperature (ca. 25 °C). The reaction mixture was then stirred and heated at 90 °C for 40 min. The reaction mixture was then allowed to cool down to room temperature. Water was added dropwise into the reaction mixture. The aqueous layer was then extracted with EtOAc thrice. The combined organic layers were washed with brine, dried over anhydrous Na_2SO_4, and concentrated under reduced pressure to yield the crude product. Subsequent purification by flash column chromatography on silica (30% EtOAc/hexanes) gave **E1** as a yellow solid (5.8 mg, yield 40%). ^{1}H NMR ($CDCl_3$, 500 MHz): δ = 3.95 (s, 3 H), 6.38 (s, 1 H), 7.03 (d, J = 8.5 Hz, 1 H), 7.42 (m, 2 H), 9.81 (s, 1 H) ppm. $^{13}C\{^1H\}$ NMR ($CDCl_3$, 126 MHz): δ = 56.2, 109.0, 114.6, 127.7, 130.0, 147.3, 151.9, 191.1 ppm. HRMS (ESI+, m/z) calculated for $C_8H_9O_3$ $[M+H]^+$ m/z = 153.0552, found 153.0551.

Compound 3-methoxy-4-((4-methoxybenzyl)oxy)benzaldehyde (**E2**) was prepared by adopting a reported procedure.[15] To a stirred suspension of **E1** (0.76 g, 5.0 mmol) in acetone (50 mL) was added K_2CO_3 (0.83 g, 5.0 mmol), n-Bu_4NI (0.37 g, 1.0 mmol), and PMBCl (0.81 mL, 6.0 mmol) at room temperature. The mixture was refluxed until all **E1** was consumed as indicated by TLC. After cooling down to room temperature, water and dichloromethane (DCM) were added. The two phases were separated, and the aqueous layer was extracted with DCM thrice. The combined organic layers were washed with brine once, dried over Na_2SO_4, filtered, and concentrated under reduced pressure to give a crude product which was further purified by column chromatography on silica (30% EtOAc/hexanes) to give **E2** as a white solid (0.61 g, yield 45%). Further purification was performed by recrystalizing the product from an appropriate solvent system to yield analytically pure sample (hexane/ EtOAc). ^{1}H NMR ($CDCl_3$, 500 MHz): δ = 3.80 (s, 3 H), 3.92 (s, 3 H), 5.16 (s, 2 H), 6.95 – 6.82 (m, 2 H), 7.00 (d, J = 8.2 Hz, 1 H), 7.39 (ddd, J = 14.1, 10.3, 5.2 Hz, 4 H), 9.83 (s, 1 H) ppm.$^{13}C\{^1H\}$ NMR ($CDCl_3$, 126 MHz): δ = 55.4, 56.1, 70.8, 109.4, 112.5, 114.2, 126.7, 128.1, 129.2, 130.3, 150.2, 153.8, 159.7, 191.0 ppm. HRMS (ESI+, m/z) calculated for $C_{16}H_{17}O_4$ $[M+H]^+$ m/z = 273.1127, found 273.1133.

Compound *N*, *N*-diethyl-3-methoxy-4-((4-methoxybenzyl)oxy)benzamide (**E3**) was prepared by adopting a reported procedure.[16] To a mixture of $CuSO_4{\cdot}5H_2O$ (1.2 mg, 5.0 μmol), diethylamine hydrochloride salt (13.2 mg, 0.12 mmol), and $CaCO_3$ (11.0 mg, 0.11 mmol) in acetonitrile (20 μL) were added **E2** (27.2 mg, 0.10 mmol) and TBHP (70 wt % in H_2O, 16 μL, 0.11 mmol) under a N_2 atmosphere at room temperature. The reaction vessel was capped and allowed to stir at 60 °C for overnight. After completion of the reaction (as indicated by TLC), water and DCM were added. The two phases were separated, and the aqueous layer was extracted with DCM thrice. The combined organic layers were washed with saturated aqueous $NaHCO_3$, H_2O (repeated three times), and once with brine, dried over Na_2SO_4, filtered, and concentrated under reduced pressure to give a crude product. This was further purified by column chromatography on silica (30% EtOAc/hexanes) to give **E3** as a pale pink solid (9.5 mg, yield 28%). ^{1}H NMR ($CDCl_3$, 500 MHz): δ = 1.16 (br, 6 H), 3.34 (br, 4 H), 3.77 (s, 3 H), 3.85 (s, 3 H), 5.06 (s, 2 H), 6.86-6.90 (m, 5 H), 7.33 (d, J = 8.4 Hz, 2 H) ppm. $^{13}C\{^1H\}$ NMR ($CDCl_3$, 126

MHz): δ = 13.0, 39.4, 55.3, 56.0, 70.8, 110.7, 113.4, 114.0, 119.1, 128.8, 129.1, 130.1, 149.0, 149.6, 159.5, 171.1 ppm. HRMS (ESI+, *m/z*) calculated for $C_{20}H_{26}N_1O_4$ $[M+H]^+$ *m/z* = 344.1862, found 344.1863.

Compound *N*, *N*-diethyl-4-hydroxy-3-methoxybenzamide (Etamivan, **26**) was prepared by adopting a reported procedure.[17] To a solution of **E3** (34.3 mg, 0.10 mmol) in dichloroethane (1 mL), $(COCl)_2$ (200 µL, in excess) was added and stirred at 50 °C for overnight. After completion of the reaction (as indicated by TLC), it was quenched in ice water. The organic layer was separated, and the aqueous layer was extracted with DCM (2 x 5 mL). The combined organic layers were washed with brine, dried over anhydrous Na_2SO_4, and concentrated under reduced pressure to give a crude product. This was then further purified by column chromatography on silica (50% EtOAc/hexanes) to give **26** as a white solid (9.7 mg, yield 44%). ^1H NMR ($CDCl_3$, 500 MHz): δ = 1.19 (br, 6 H), 3.42 (br, 4 H), 3.90 (s, 3 H), 5.82 (s, 1 H), 6.89 (d, *J* = 0.8 Hz, 2 H), 6.95 (s, 1 H) ppm. $^{13}C\{^1H\}$ NMR ($CDCl_3$, 75 MHz): δ = 13.7, 42.6, 56.2, 110.1, 114.1, 119.9, 129.0, 146.6, 146.8, 171.4 ppm. HRMS (ESI+, *m/z*) calculated for $C_{12}H_{18}N_1O_3$ $[M+H]^+$ *m/z* = 224.1287, found 224.1287.

Procedures for the optimization of the photocatalytic reactions

Screening of HAT additives

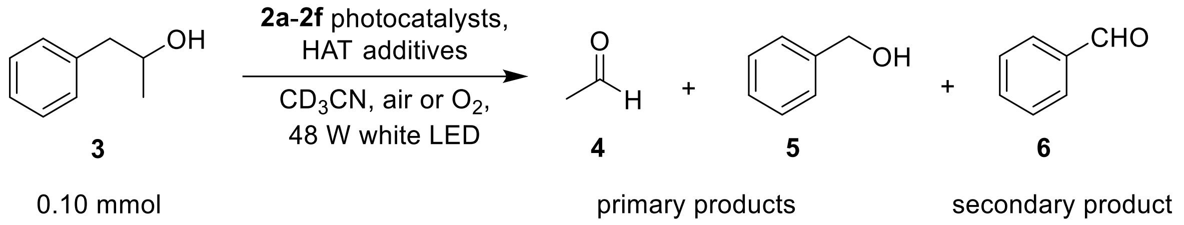

Photocatalytic experiments were carried out in NMR tubes. In a general procedure, **3** (0.10 mmol), **2e** (2.4 mg, 5.0 µmol), and hydrogen atom transfer (HAT) additives (0-0.80 mmol) were dissolved in CD_3CN (0.50 mL), and 1,1,2,2-tetrachloroethane (10.6 µL, 0.10 mmol) was added as an internal standard to aid in the calculation of the conversion and yield of the products. The NMR tube was fitted with a needle to supply air into the reaction vessel with minimal evaporation throughout the experiment. The reaction mixture was irradiated with 48 W white light emitting diodes (LEDs) at ambient temperature. A continuous water circulator was used to maintain the reaction temperatures below 30 °C. The NMR tube containing the reaction mixture was used in the NMR spectroscopic experiments before and after the light irradiation to allow us to monitor the progress of the reaction. The reaction products were identified by ^1H NMR spectroscopy by comparing the peaks from the reaction mixture's spectrum to those of the commercially available compounds (where available). The results are summarized in the table below.

Table S1 Full screening conditions of different HAT additives to maximize the primary C-C bond cleavage products. The optimized reaction conditions are emboldened in blue.

Entry	Additive (mmol)	Irradiation time (h)	Substrate conversion (%)	Distribution of products (%)		
				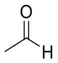	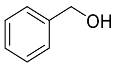	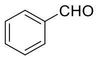
1	-	6	89	47	32	34
2	Ph_3CH (0.15)	14	71	38	33	10
3	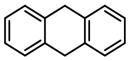 (0.15)	14	>95	82	58	10
4	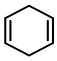 (0.15)	14	>95	68	56	16
5	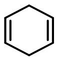 (0.15)	20	>95	84	55	24
6	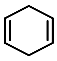 (0.10)	20	>95	73	49	28
7	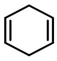 (0.40)	20	>95	87	50	19
8	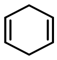 (0.80)	14	>95	85	58	11
**9**	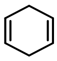 **(0.40)**	**14**	**>95**	**90**	**60**	**11**

Examining the effects of using air or pure O_2 on the reaction

The photocatalytic experiments were carried out in NMR tubes. In a typical procedure, **3** (14 µL, 0.10 mmol), **2e** (2.4 mg, 5.0 µmol), and 1,4-cyclohexadiene (1,4-CHD, 40 µL, 0.40 mmol) were dissolved in CD_3CN (0.50 mL), and 1,1,2,2-tetrachloroethane (10.6 µL, 0.10 mmol) was added as an internal standard to aid calculations of the conversion and yield of the products. The NMR tube was fitted with a needle to supply air or O_2 gas from a balloon into the reaction vessel throughout the experiment. The reaction mixture was irradiated with white LEDs (48 W) at ambient temperature. A continuous water circulator was used to maintain the reaction temperatures below 30 °C. The NMR tube containing the reaction mixture was monitored at specific time intervals by ^1H NMR spectroscopy before and after irradiation, to follow the progress of the reaction. The reaction products were identified by ^1H NMR spectroscopy by comparing the peaks from the reaction mixture's spectrum to those of the commercially available compounds, where available. The results are summarized in the table below.

Table S2 Effects of air or O_2 on the substrate conversion and product distribution. The optimal conditions are emboldened in blue.

Entry	Reaction time (h)	Catalyst (mol%)	Environment	Substrate conversion (%)	Distribution of products (%)		
					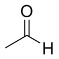	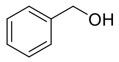	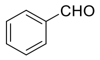
1	4	**2e** (5)	air	59	46	33	2
2	8	**2e** (5)	air	84	65	50	8
3	10	**2e** (5)	air	92	68	54	10
**4**	**14**	**2e (5)**	**air**	**>95**	**90**	**60**	**11**
5	4	**2e** (5)	O_2	63	55	40	4
6	8	**2e** (5)	O_2	81	64	51	8
7	10	**2e** (5)	O_2	89	69	53	11

Varying the catalyst loading

Photocatalytic experiments were carried out in NMR tubes. In an exemplary experiment, **3** (14 µL, 0.10 mmol), **2e** (0-7.2 mg, 0-15.0 µmol), and 1,4-CHD (40 µL, 0.40 mmol) were dissolved in CD_3CN (0.50 mL), and 1,1,2,2-tetrachloroethane (10.6 µL, 0.10 mmol) was added as an internal standard to aid in the calculation of the conversion and yield of the products. The NMR tube was fitted with a needle to supply air into the reaction vessel throughout the experiment, while minimizing evaporation of the solvent. The reaction mixture was irradiated with white LEDs (48 W) at ambient temperature. A continuous water circulator was used to maintain the reaction temperatures below 30 °C. The NMR tube containing the reaction mixture was used in the NMR spectroscopic experiments before and after 14 h of irradiation to allow us to monitor the progress of the reaction. The reaction products were identified by ^{1}H NMR spectroscopy by comparing the peaks from the reaction mixture's spectrum to those of the commercially available compounds. The results are summarized in the table below.

Table S3 Effects of various catalyst loadings on substrate conversion and product distribution. The optimal conditions are emboldened in blue.

Entry	Reaction time (h)	Catalyst	Catalyst loading (mol%)	Substrate conversion (%)	Distribution of products (%)		
					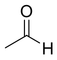	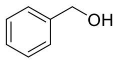	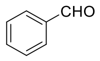
1	14	**2e**	0.0	-	-	-	-
2	14	**2e**	2.5	81	64	51	9
**3**	**14**	**2e**	**5.0**	**>95**	**90**	**60**	**11**
4	14	**2e**	10	>95	90	56	10
5	14	**2e**	15	>95	91	54	14

Screening of vanadium catalysts

Photocatalytic experiments were carried out in NMR tubes. In a typical experiment, **3** (14 µL, 0.10 mmol), the vanadium catalyst (**2a**-**2f**, 1.7-2.6 mg, 5.0 µmol), and 1,4-CHD (40 µL, 0.40 mmol) were dissolved in CD_3CN (0.50 mL), and 1,1,2,2-tetrachloroethane (10.6 µL, 0.10 mmol) was added as an internal standard to aid in the calculation of the conversion and yield of the products. The NMR tube was fitted with a needle to supply air into the reaction vessel throughout the experiment. The reaction mixture was irradiated with white LEDs (48 W) at ambient temperature. A continuous water circulator was used to maintain the reaction temperatures below 30 °C. The NMR tube containing the reaction mixture was used in the NMR spectroscopic experiments before and after 14 h of light irradiation to allow us to monitor the progress of the reaction. The reaction products were identified by ^{1}H NMR spectroscopy by comparing the peaks from the reaction mixture's spectrum to those of the commercially available compounds. The results are summarized in the table below.

Table S4 Effects of different catalysts on substrate conversion and product distribution. The optimal conditions are emboldened in blue.

**Entry**	**Reaction time (h)**	**Catalyst**	**Catalyst loading (mol%)**	**Substrate conversion (%)**	**Distribution of products (%)**		
					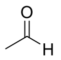	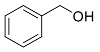	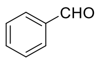
1	14	**2a**	5.0	80	62	60	7
2	14	**2b**	5.0	85	70	63	8
3	14	**2c**	5.0	64	56	44	12
4	14	**2d**	5.0	93	74	63	10
**5**	**14**	**2e**	**5.0**	**>95**	**90**	**60**	**11**
6	14	**2f**	5.0	>95	76	55	25

Examining the effects of different oxidants

The photocatalytic experiments were carried out in J. Young NMR tubes *under a N_2 atmosphere*. In a typical procedure, **3** (14 μL, 0.10 mmol) and **2e** (2.4 mg, 5.0 μmol) were dissolved in CD_3CN (0.50 mL), and 1,1,2,2-tetrachloroethane (10.6 μL, 0.10 mmol) was added as an internal standard to aid the calculations of the conversions and yields of the products. To the NMR tube was then added 1.5 eq. (0.15 mmol) of hydrogen peroxide (H_2O_2, 35% aq.), *tert*-butyl hydroperoxide (TBHP, 70% aq.), or copper(II) triflate. Each reaction mixture was irradiated with a white LED (48 W) at ambient temperature. A continuous water circulator was used to maintain the reaction temperatures below 30 °C. The sealed NMR tube containing the reaction mixture was monitored at specific time intervals by ^{1}H NMR spectroscopy before and after irradiation to provide information about the progress of the reaction. The reaction products were identified by ^{1}H NMR spectroscopy by comparing the peaks from each reaction mixture's spectrum to those of the commercially available compounds, where available. The results are summarized in the table below.

Table S5 Effects of alternative oxidants on substrate conversion and product distribution. The reaction in Entry 3 was found to generate completely different products from the expected oxidative C-C bond cleavage.

**Entry**	**Reaction time (h)**	**Catalyst (mol%)**	**Oxidant (1.5 eq.)**	**Substrate conversion (%)**	**Distribution of products (%)**				
					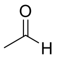	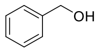	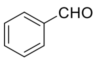	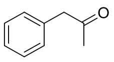	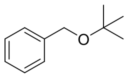
1	19	**2e (5.0)**	H_2O_2	60	43	17	34	7	-
2	19	**2e (5.0)**	TBHP	60	18	19	10	10	14
3	19	**2e (5.0)**	Cu(II) triflate	>95	-	-	-	-	-

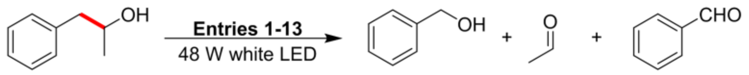

3 4 5 6
major products minor product

Entry	HAT agents (eq.)	Catalyst (mol % loading)	Environment (air or O_2)	Reaction time (h)	Substrate conversion (%)	Distribution of products (%)		
						**4**	**5**	**6**
1	-	**2e** (5.0)	air	6	89	32	47	34
2	Ph_3CH (1.5)	**2e** (5.0)	air	14	71	33	38	10
3	9,10-DHA (1. 5)	**2e** (5.0)	air	14	>95	58	82	10
4	1,4-CHD (1.5)	**2e** (5.0)	air	14	>95	56	68	16
**5**	**1,4-CHD (4.0)**	**2e (5.0)**	**air**	**14**	**>95**	**60**	**90**	**11**
6	1,4-CHD (4.0)	**2e** (5.0)	O_2	10	89	53	69	11
7	1,4-CHD (4.0)	-	air	14	-	-	-	-
8*	1,4-CHD (4.0)	**2e** (5.0)	O_2	24	-	-	-	-
9	1,4-CHD (4.0)	**2e** (2.5)	air	14	81	51	64	9
10	1,4-CHD (4.0)	**2e** (10)	air	14	>95	56	90	10
11	1,4-CHD (4.0)	**2a** (5.0)	air	14	80	60	62	7
12	1,4-CHD (4.0)	**2c** (5.0)	air	14	64	44	56	12
13	1,4-CHD (4.0)	**2f** (5.0)	air	14	>95	55	76	25
14**	1,4-CHD (4.0)	**2e** (5.0)	air	26	>95	78	67	17

Figure S2 Optimization of conditions for the photocatalytic C-C bond cleavage in **3**. The optimized reaction conditions are emboldened. *No light applied, but heated at 80 °C. **The photocatalytic experiments were conducted at 10 times (1.0 mmol, 136 mg of **3**) the scale of the usual optimal conditions.

Procedures for the mechanistic screening of substrates

The photocatalytic experiments were carried out in NMR tubes. In a typical procedure, **3** (14 µL, 0.10 mmol), **2e** (2.4 mg, 5.0 µmol), 1,4-CHD (40 µL, 0.40 mmol) and one eq. of a suitably substituted benzene (0.10 mmol) were dissolved in CD_3CN (0.50 mL), and 1,1,2,2-tetrachloroethane (10.6 µL, 0.10 mmol) was added as an internal standard to aid calculations of the conversion and yield of the products. The NMR tube was fitted with a needle to supply air into the reaction vessel throughout the experiment. The reaction mixture was irradiated with white LEDs (48 W) at ambient temperature. A continuous water circulator was used to maintain the reaction temperatures below 30 °C. The NMR tube containing the reaction mixture was used in the NMR spectroscopic experiments before and after light irradiation to allow us to monitor the progress of the reaction. The reaction products were identified by ^1H NMR spectroscopy by comparing the peaks from the reaction mixture's spectrum to those of the commercially available compounds, where available. The results are summarized in Figure S3 below.

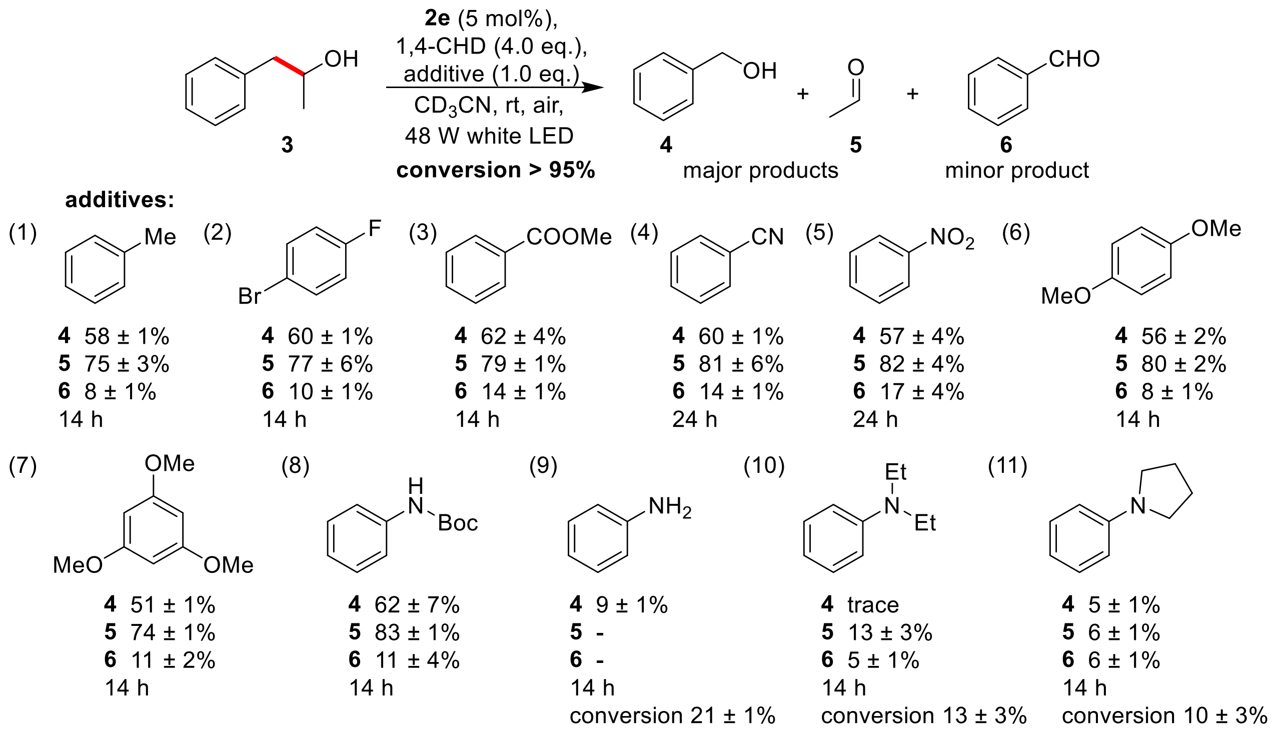

Figure S3 Mechanistic screening of substrates proposed by Glorius *et al.* Note that we did not synthesize each substrate with the different functional group, but only introduced the substituted benzene molecule. The derivatized benzenes displayed different effects on the rate and product distribution of photocatalytic C-C bond cleavage in **3** when added in stoichiometric amounts. In particular, the initially detrimental anilines were well tolerated once protected with a *tert*-butyloxycarbonyl (Boc) group.

Procedures for the photocatalytic reactions with small and macromolecular alcohols

The photocatalytic experiments were carried out in NMR tubes. In a typical reaction, the substrate (10-100 μmol), **2e** (0.60-2.4 mg, 1.0-5.0 μmol), and 1,4-CHD (40 μL, 400 μmol) were dissolved in CD_3CN (0.50-0.60 mL) or suspended (if poorly soluble) in a CD_3CN/toluene-d_8 mixture (0.50:0.10 mL, respectively), and 1,1,2,2-tetrachloroethane (1.1-10.6 μL, 10-100 μmol) was added as an internal standard to aid calculations of the conversion and yield of the products. The antioxidant 1,4-CHD was not added for the reactions of the macromolecular substrates to accelerate the reaction rates. The NMR tube was then fitted with a needle to supply air into the reaction vessel throughout the experiment. The reaction mixture was irradiated with white LEDs (48 W) at ambient temperature. A continuous water circulator was used to maintain the reaction temperatures below 30 °C for soluble substrates. The NMR tube containing the reaction mixture was used in the NMR spectroscopic experiments before and after light irradiation to allow us to monitor the progress of the reaction. The reaction products were identified by ^{1}H NMR spectroscopy by comparing the peaks from the reaction mixture's spectra to those of the commercially available compounds, where available. Additionally, control experiments without **2e**, with **2e** in the dark, and with **2e** at elevated temperatures, were conducted under otherwise identical reaction conditions and with the same quantities of reagents.

Reaction of PEG 400 (34)

In a typical reaction, 5.6 μL (16 μmol) of **34** were dissolved in 0.50 mL of CD_3CN, along with 2.4 mg of **2e** (5 μmol), 10.6 μL(100 μmol) of 1,1,2,2-tetrachloroethylene, and 8.1 μL of MeOH or MeOD-d_4 (200 μmol). The reaction mixture was irradiated with white LEDs at temperatures below 30 °C for 2.5 days. Subsequently, the reaction mixture was introduced into a round bottom flask and the volatile compounds were vacuum transferred using liquid N_2 into another round bottom flask. The internal standard (1,1,2,2-tetrachloroethane) was added and the volatile products (methyl formate, formic acid, and others) were quantified by ^{1}H NMR spectroscopy and confirmed by GC. The conversion of **34** is more than 95% and the yield of methyl formate was 54%, as observed by integrating only the formate signal in the ^{1}H NMR spectrum of the crude reaction mixture in the experiment where MeOD-d_4 was used.

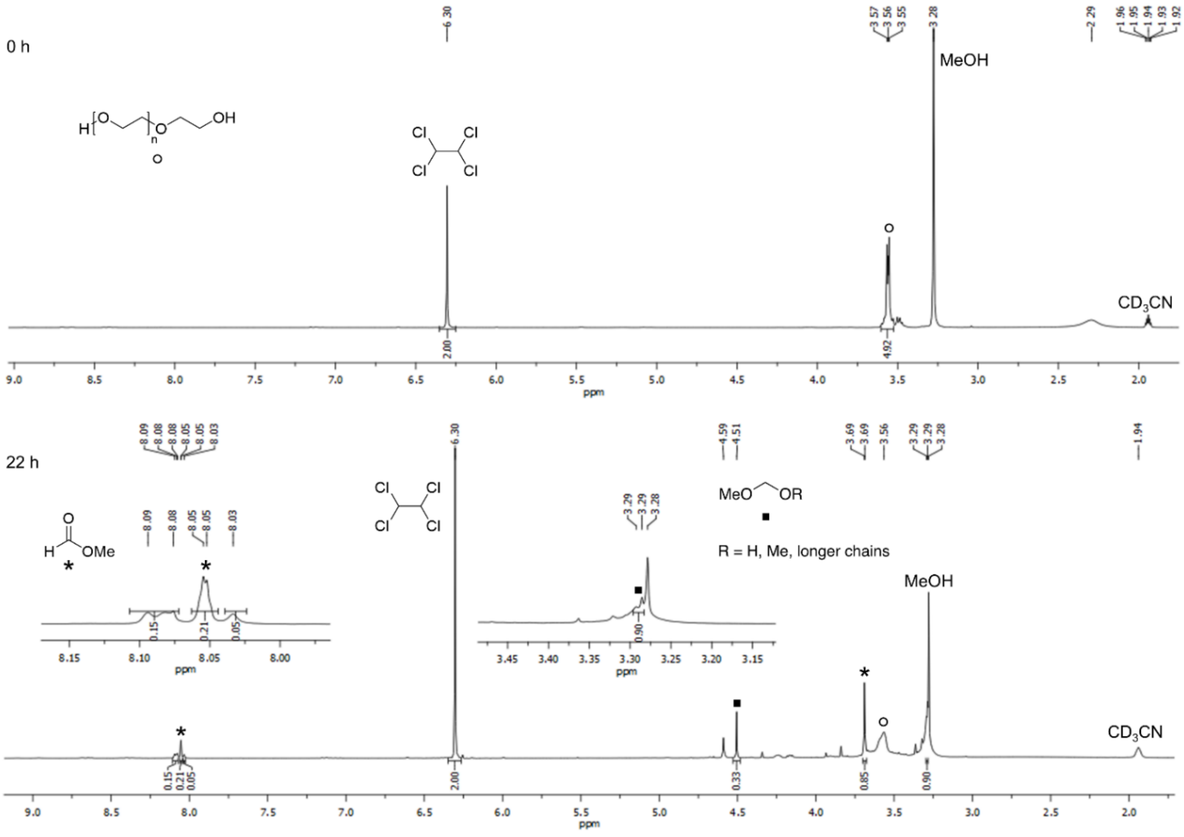

Figure S4 The ^{1}H NMR spectra of **34** before (top) and after 22 h (bottom) of visible light irradiation. The reaction products include methyl formate (star) and possibly the hemiacetal or acetal of formaldehyde (square). The latter is unstable and the signal is diminished at full conversion as seen in Figure 6 in the main manuscript.

Reaction of PCL-PEG-PCL (35)

In a general reaction, 2.3 mg (0.40 µmol) of **35** were dissolved in 0.50 mL of CD_3CN, along with 2.4 mg of **2e** (5 µmol) and 10.6 µL(100 µmol) of 1,1,2,2-tetrachloroethylene. The reaction mixture was irradiated with white LEDs at temperatures below 30 °C for 2 days. After that, the reaction mixture was introduced into a round bottom flask and the volatile compounds were vacuum transferred using liquid N_2 into another round bottom flask. The internal standard (1,1,2,2-tetrachloroethane) was added and the volatile products (formic acid and other unidentified oxygenated compounds) were quantified by ^{1}H NMR spectroscopy and confirmed by GC. The conversion of **35** is essentially quantitative and the yield of formic acid is 67%, as observed in the ^{1}H NMR spectrum of the crude reaction mixture.

Reaction of 1-hexadecanol (36)

In a typical procedure, 12 mg (50 µmol) of **36** were suspended in 0.60 mL of CD_3CN, along with 1.2 mg of **2e** (2.5 µmol), 5.3 µL(50 µmol) of 1,1,2,2-tetrachloroethylene. The reaction mixture was irradiated with white LEDs at 55 °C to allow for complete dissolution. After 5 days, the conversion of **36** is more than 95% and the yield of formic acid is 66%, as observed in the ^{1}H NMR spectrum of the crude reaction mixture.

Reaction of PE-PEG (37)

In a general reaction, 1.3 mg (2.3 µmol) of **37** were suspended in a mixture of 0.50 mL of CD_3CN and 0.10 mL of toluene-d_8, to which 0.60 mg (1.0 µmol) of **2e** and 1.3 µL (10 µmol) of 1,1,2,2-tetrachloroethylene were added. The mixture was irradiated with white LEDs at 85 °C to allow for complete dissolution. After 7 days of irradiation, the conversion of **37** is more than 95% and the yield of formic acid is 6%, as observed in the ^{1}H NMR spectrum of the crude reaction mixture. Due to the elevated temperatures needed to dissolve **37**, more volatile products presumably evaporated, and even the formic acid yield is just the lower limit.

Reaction of PE-monoalcohol (38)

In a typical reaction, 0.60 mg (1.3 µmol) of **38** were suspended in a mixture of 0.50 mL of CD_3CN and 0.10 mL of toluene-d_8 to which 0.60 mg (1.0 µmol) of **2e** and 1.3 µL (10 µmol) of 1,1,2,2-tetrachloroethylene were added. The mixture was irradiated with white LEDs at 85 °C. After 6 days of irradiation, the conversion of **38** is more than 95% and the yield of formic acid is 6%, as observed in the ^{1}H NMR spectrum of the crude reaction mixture. Due to the elevated temperatures needed to dissolve **38** as well, more volatile products presumably evaporated, and even the formic acid yield is just the lower limit.

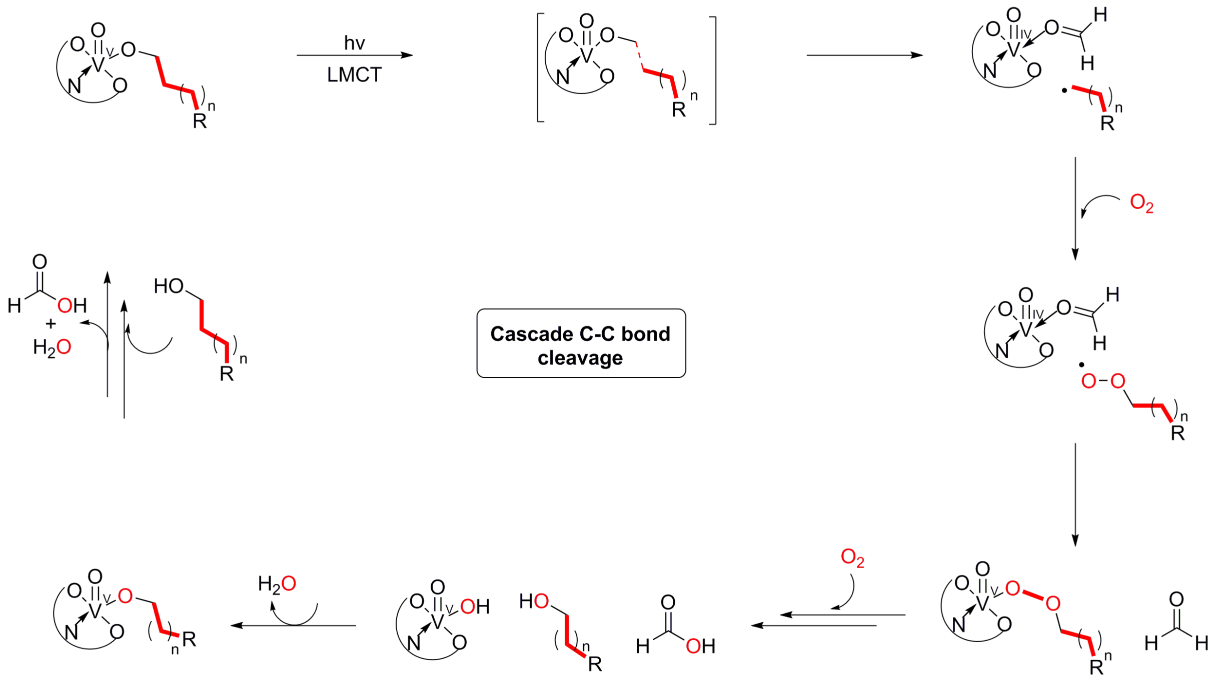

Figure S5 Proposed reaction mechanism for the cascade C-C bond cleavage in polyethylene-monoalcohol. The bonds that undergo cleavage are highlighted in bold and red.

Identification of reaction products

Products of the photocatalytic reactions that are new compounds have been identified after isolation by preparative TLC and have been characterized by NMR spectroscopy and high-resolution mass spectrometry. The identities of known compounds were verified by comparing the peak positions in the ^{1}H NMR spectrum, as well as their GC-MS/LC-MS spectra, with those of the commercially available compounds. For performing a preparative TLC analysis, at least three NMR tube-scale reaction mixtures were combined to provide sufficient amounts of products.

Calculations for the amount of CO_2 amount produced during the PE-monoalcohol (38) photolysis

The concentration of CO_2 in the headspace gas sample was determined to be 300 ppm (equivalent to 300 mg L^{-1}) by gas chromatography. We estimated that the total volume of the reaction vessel was 3.8 mL by using a liquid to completely fill up the headspace. Hence, the amount of CO_2 produced after the photolysis of **38** was calculated to be 1.14 mg (26 µmol). The amount of **38** (average molecular weight of 460 according to the chemical supplier) used for the photolysis was 0.60 mg (1.3 µmol). The number of repeating ethylene units is 16 and each unit can potentially produce two CO_2 molecules. Hence, the theoretical number of moles of CO_2 produced is 42 µmol. Based on this theoretical yield, the estimated amount of CO_2 produced is 62%.

References

1. S. Gazi, M. Đokić, A. M. P. Moeljadi, R. Ganguly, H. Hirao, H. S. Soo, *ACS Catal*. **2017**, *7*, 4682-4691.
2. S. V. Chankeshwara, A. K. Chakraborti, *Org. Lett*. **2006**, *8*, 3259-3262.
3. L. W. Bieber, E. C. Storch, I. Malvestiti, M. F. da Silva, *Tetrahedron Lett*. **1998**, *39*, 9393-9396.
4. Y. Su, X. Sun, G. Wu, N. Jiao, *Angew. Chem., Int. Ed*. **2013**, *52*, 9808-9812.
5. G. J. Lovinger, M. D. Aparece, J. P. Morken, *J. Am. Chem. Soc*. **2017**, *139*, 3153-3160.
6. A. K. Dubey, D. Goswami, A. Chattopadhyay, *Arkivoc* **2010**, *2010*, 137-145.
7. M. Verdecchia, C. Tubaro, A. Biffis, *Tetrahedron Lett*. **2011**, *52*, 1136-1139.
8. C. A. Loeschorn, M. Nakajima, P. J. McCloskey, J.-P. Anselme, *J. Org. Chem*. **1983**, *48*, 4407-4410.
9. C. J. Easton, A. J. Ivory, C. A. Smith, *J. Chem. Soc., Perkin Trans. 2* **1997**, *1997*, 503-508.
10. L. Crespin, L. Biancalana, T. Morack, D. C. Blakemore, S. V. Ley, *Org. Lett*. **2017**, *19*, 1084-1087.
11. D. S. Noyce, D. R. Hartter, F. B. Miles, *J. Am. Chem. Soc*. **1968**, *90*, 3794-3796.
12. A. B. Terent'ev, T. T. Vasil'eva, A. A. Ambartsumyan, O. V. Chakhovskaya, N. E. Mysova, K. A. Kochetkov, *Russ. J. Org. Chem*. **2009**, *45*, 1181-1184.
13. G. R. Allan, A. J. Carnell, *J. Org. Chem*. **2001**, *66*, 6495-6497.
14. R. H. Prager,Y. T. Tan, *Tetrahedron Lett*. **1967**, *8*, 3661-3664.
15. P. Ploypradith, P. Cheryklin, N. Niyomtham, D. R. Bertoni, S. Ruchirawat, *Org. Lett*. **2007**, *9*, 2637-2640.
16. S. C. Ghosh, J. S.Y. Ngiam, A. M. Seayad, D. T. Tuan, C. L. L. Chai, A. Chen, *J. Org. Chem*. **2012**, *77*, 8007-8015.
17. A. Ilangovan, K. Anandhan, M. P. Kaushik, *Tetrahedron Lett*. **2015**, *56*, 1080-1084.

Identification of reaction products

Product 4-fluorobenzaldehyde

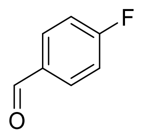

HRMS (ESI+, *m/z*) calculated for C_7H_6FO $[M+H]^+$ *m/z* = 125.0403, found 125.0405.

Product 4-bromobenzaldehyde

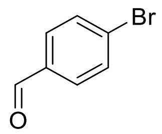

HRMS (ESI+, *m/z*) calculated for C_7H_6BrO $[M+H]^+$ *m/z* = 184.9602, found 184.9598.

Product 4-(trifluoromethyl)benzaldehyde

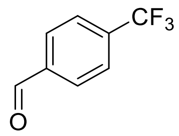

HRMS (ESI+, *m/z*) calculated for $C_8H_6F_3O$ $[M+H]^+$ *m/z* = 175.0371, found 175.0374.

Product 4-methylbenzaldehyde

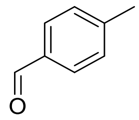

HRMS (ESI+, *m/z*) calculated for C_8H_9O $[M+H]^+$ *m/z* = 121.0653, found 121.0654.

Product 2,6-dimethylbenzaldehyde

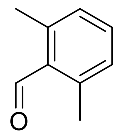

HRMS (ESI+, *m/z*) calculated for $C_9H_{11}O$ $[M+H]^+$ *m/z* = 135.0810, found 135.0813.

Product 3,4-dimethoxybenzaldehyde

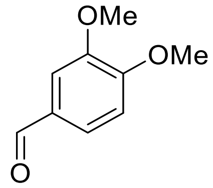

HMRS (ESI+, *m/z*) calculated for $C_9H_{11}O_3$ $[M+H]^+$ *m/z* = 167.0708, found 167.0713.

Product 4-nitrobenzaldehyde

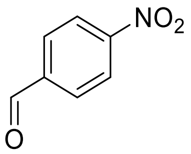

HRMS (ESI+, *m/z*) calculated for $C_7H_6NO_3$ $[M+H]^+$ *m/z* = 152.0348, found 152.0349.

Product 2,3,4,5,6-pentafluorobenzaldehyde

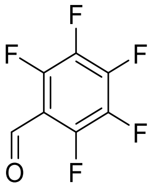

HRMS (ESI+, *m/z*) calculated for $C_7H_2F_5O$ $[M+H]^+$ *m/z* = 197.0026, found 197.0027.

Product 4-(4,4,5,5-tetramethyl-1,3,2-dioxaborolan-2-yl)benzaldehyde

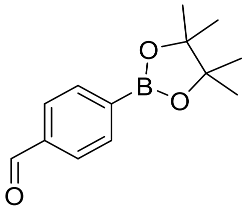

HRMS (ESI+, *m/z*) calculated for $C_{13}H_{18}O_3B$ $[M+H]^+$ *m/z* = 233.1349, found 233.1358.

Product mesitylmethanol

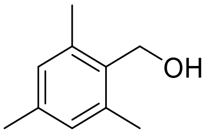

HMRS (ESI+, *m/z*) calculated for $C_{10}H_{15}O$ $[M+H]^+$ *m/z* = 151.1123, found 151.1125.

Product 2,4,6-trimethylbenzaldehyde

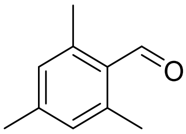

HRMS (ESI+, *m/z*) calculated for $C_{10}H_{13}O$ $[M+H]^+$ *m/z* = 149.0966, found 149.0972.

Product (2,4,6-triisopropylphenyl)methanol

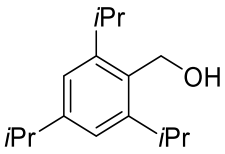

HMRS (ESI+, *m/z*) calculated for $C_{16}H_{27}O$ $[M+H]^+$ *m/z* = 235.2062, found 235.2057.

Product 2,4,6-triisopropylbenzaldehyde

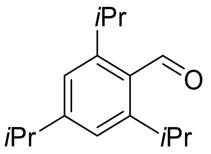

HMRS (ESI+, *m/z*) calculated for $C_{16}H_{25}O$ $[M+H]^+$ *m/z* = 233.1905, found 233.1904.

Product (4-(tert-butyl)phenyl)methanol

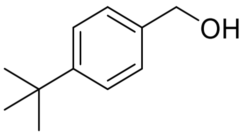

HRMS (ESI+, *m/z*) calculated for $C_{11}H_{17}O$ $[M+H]^+$ *m/z* = 165.1279, found 165.1274.

Product 4-(*tert*-butyl)benzaldehyde

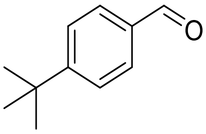

HRMS (ESI+, *m/z*) calculated for $C_{11}H_{15}O$ $[M+H]^+$ *m/z* = 163.1123, found 163.1127.

Product (3,4-dimethoxyphenyl)methanol

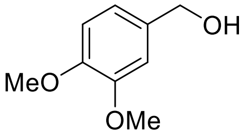

HRMS (ESI+, *m/z*) calculated for $C_9H_{13}O_3$ $[M+H]^+$ *m/z* = 169.0865, found 169.0866.

Product thiophen-3-ylmethanol

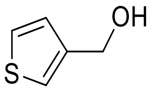

HRMS (ESI+, *m/z*) calculated for C_5H_7OS [M+H]+ *m/z* = 115.0218, found 115.0224.

Product thiophene-3-carbaldehyde

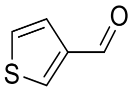

HRMS (ESI+, *m/z*) calculated for C_5H_5OS $[M+H]^+$ *m/z* = 113.0061, found 113.0059.

Product naphthalen-1-ylmethanol

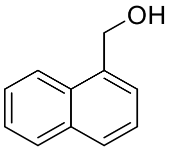

HRMS (ESI+, *m/z*) calculated for $C_{11}H_{11}O$ $[M+H]^+$ *m/z* = 159.0810, found 159.0814.

Product 1-naphthaldehyde

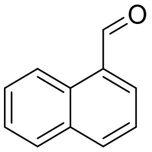

HRMS (ESI+, *m/z*) calculated for $C_{11}H_9O$ $[M+H]^+$ *m/z* = 157.0653, found 157.0654.

Spectral data

NMR spectra of substrates

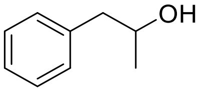

^{1}H NMR

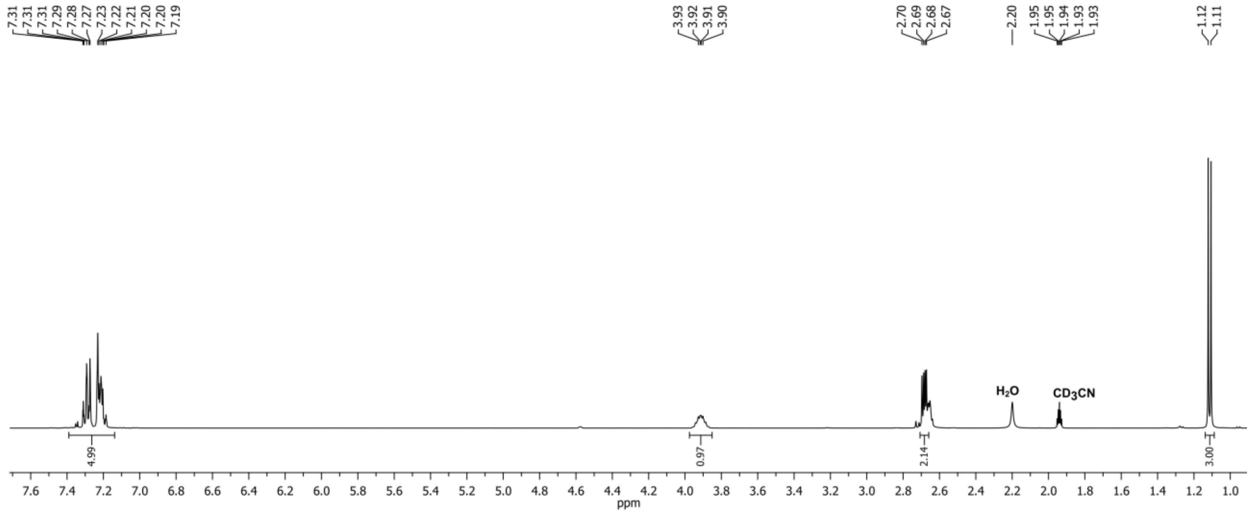

^{13}C{^{1}H} NMR

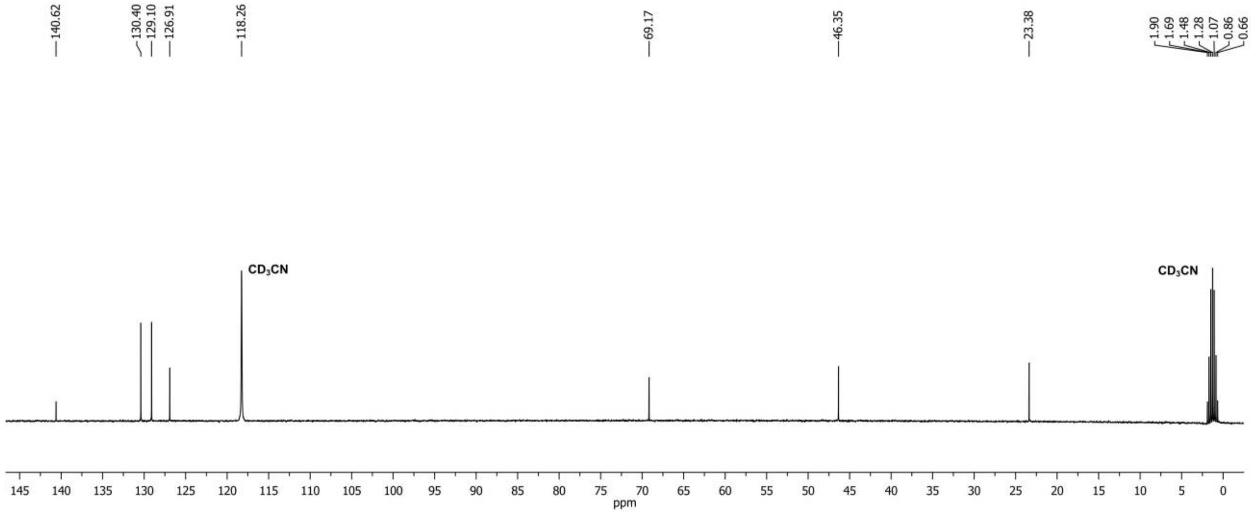

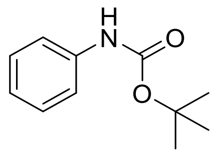

^{1}H NMR

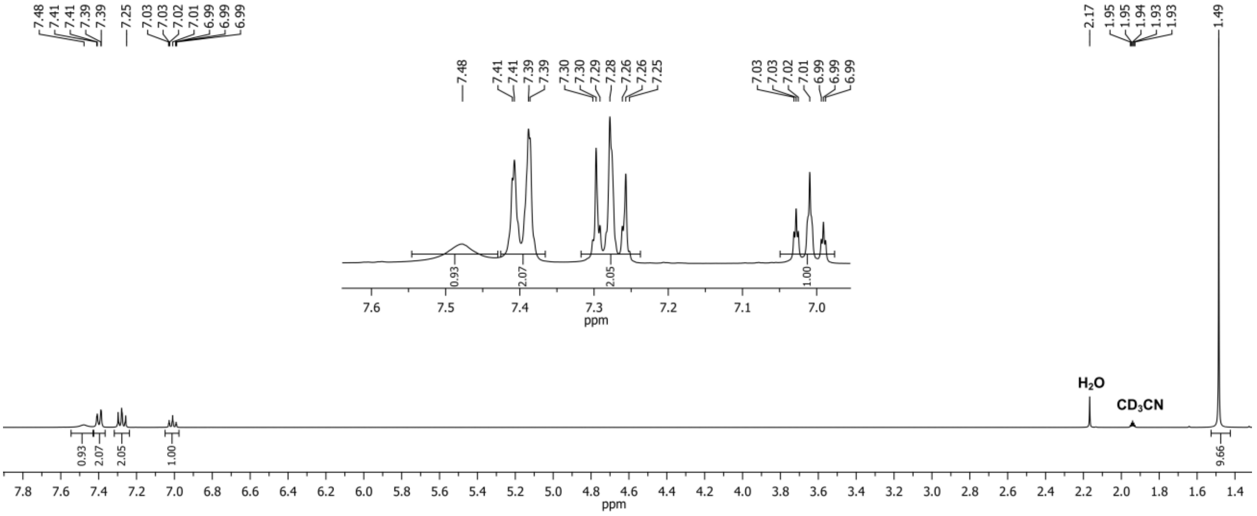

^{13}C{^{1}H} NMR

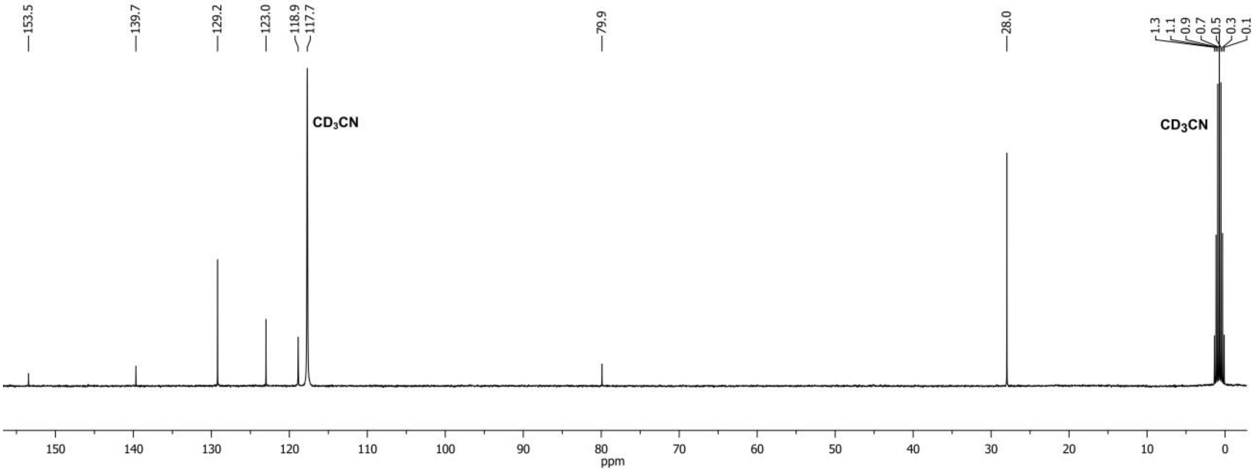

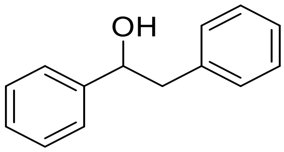

^{1}H NMR

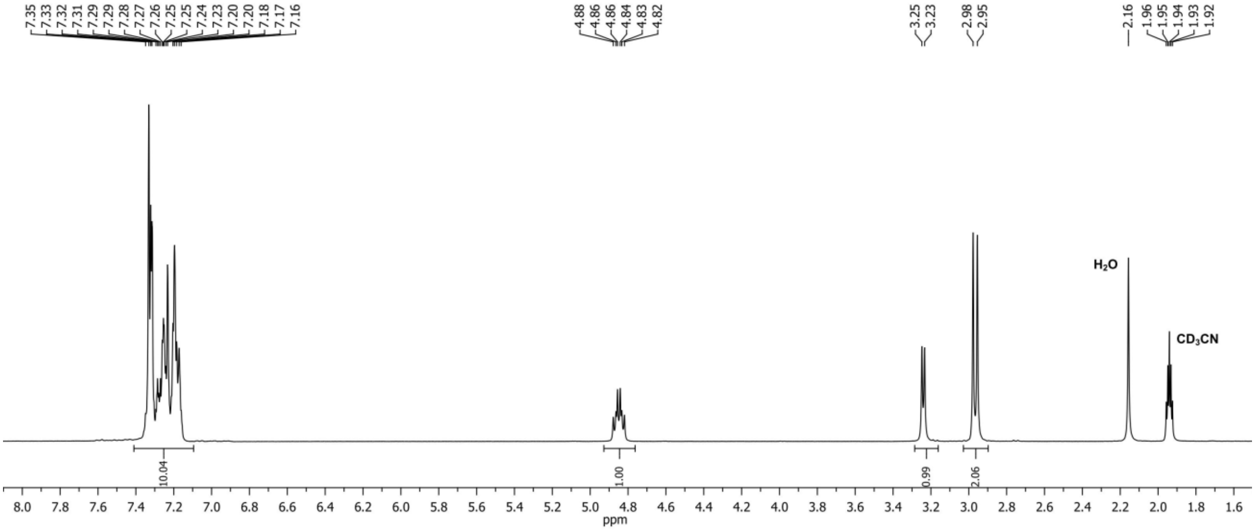

^{13}C{^{1}H} NMR

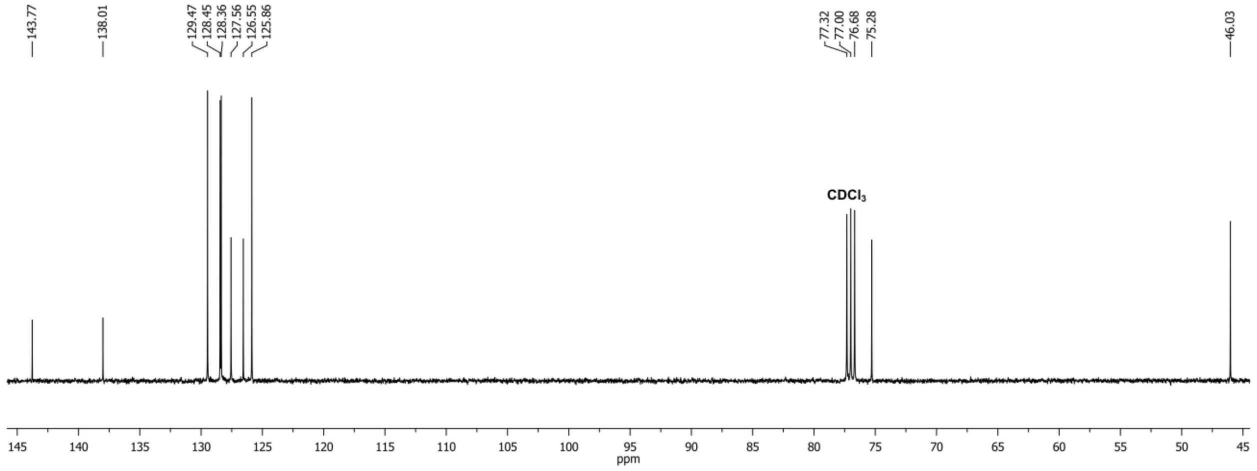

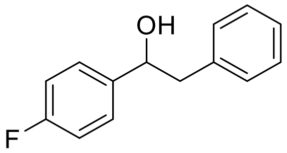

^{1}H NMR

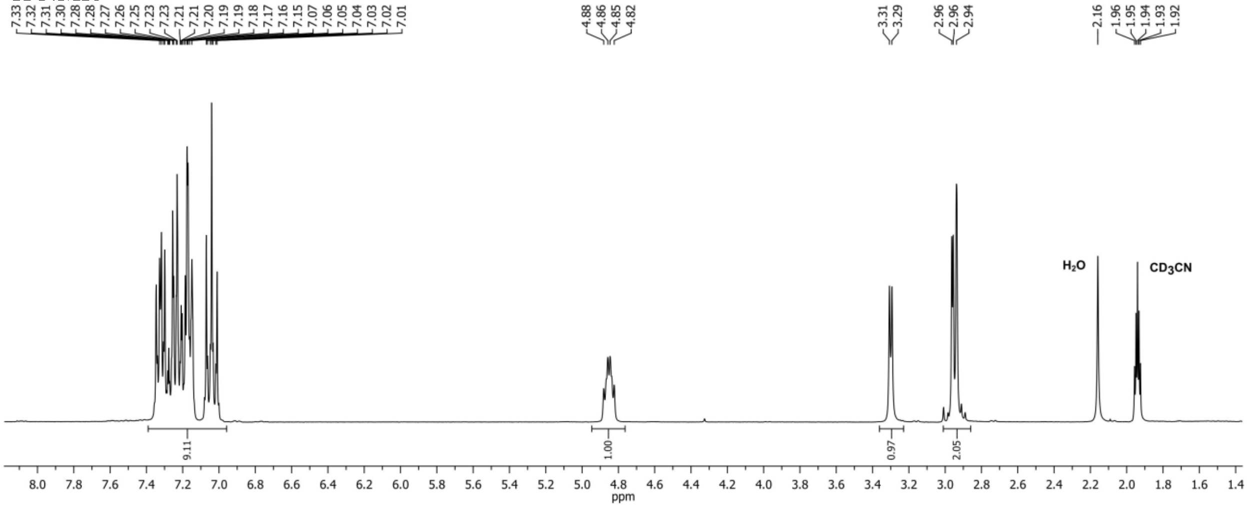

^{13}C{^{1}H} NMR

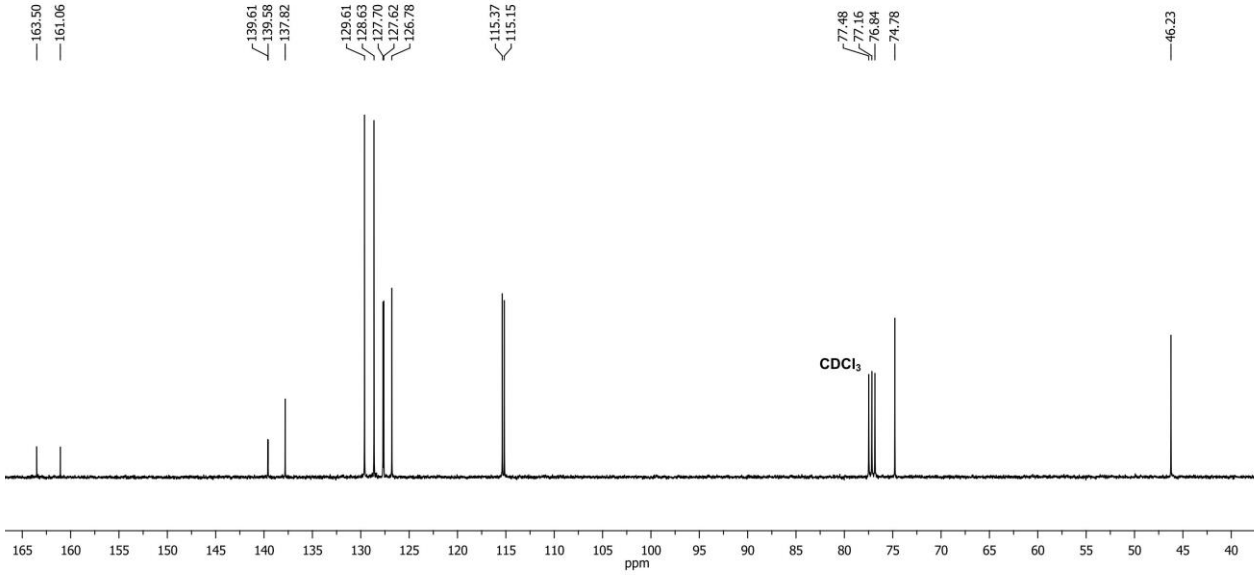

$^{19}F\{^{1}H\}$ NMR

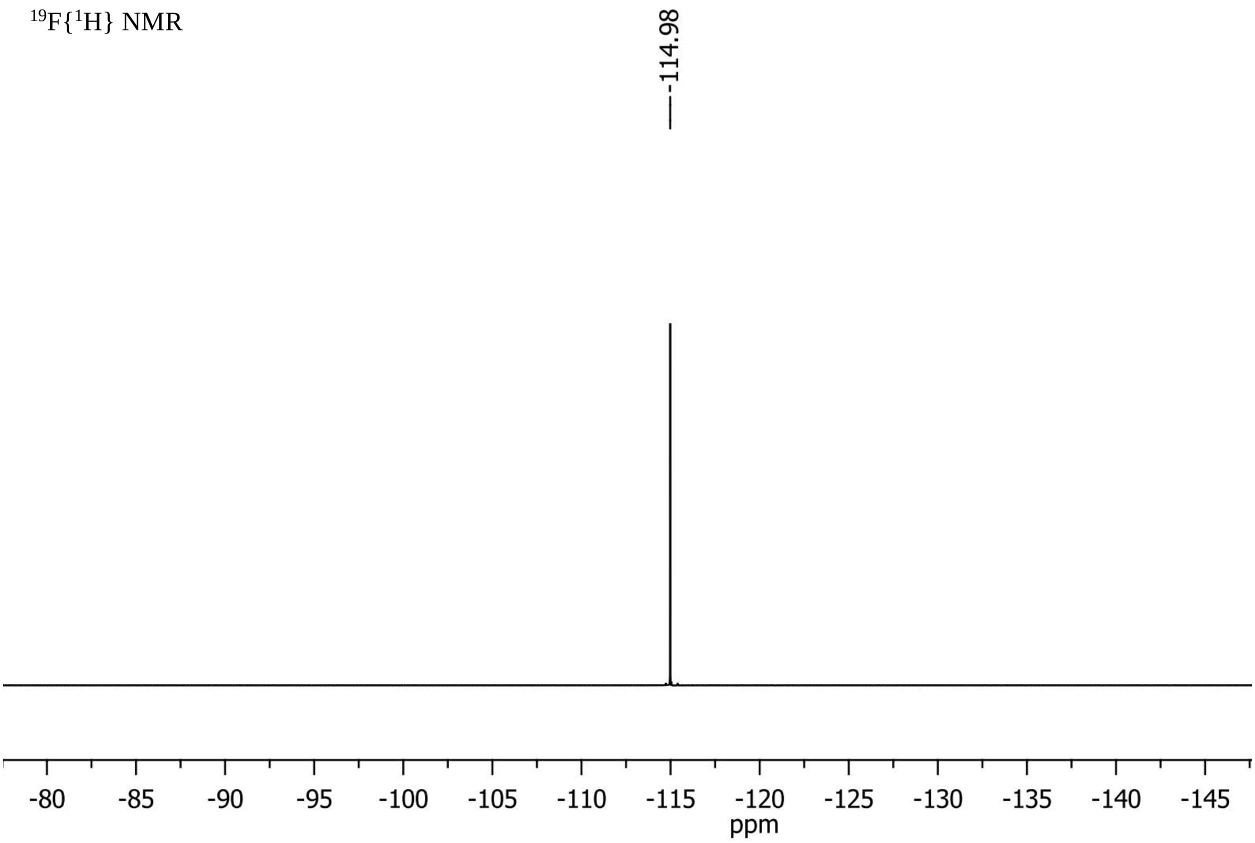

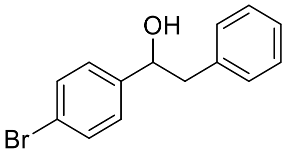

^{1}H NMR

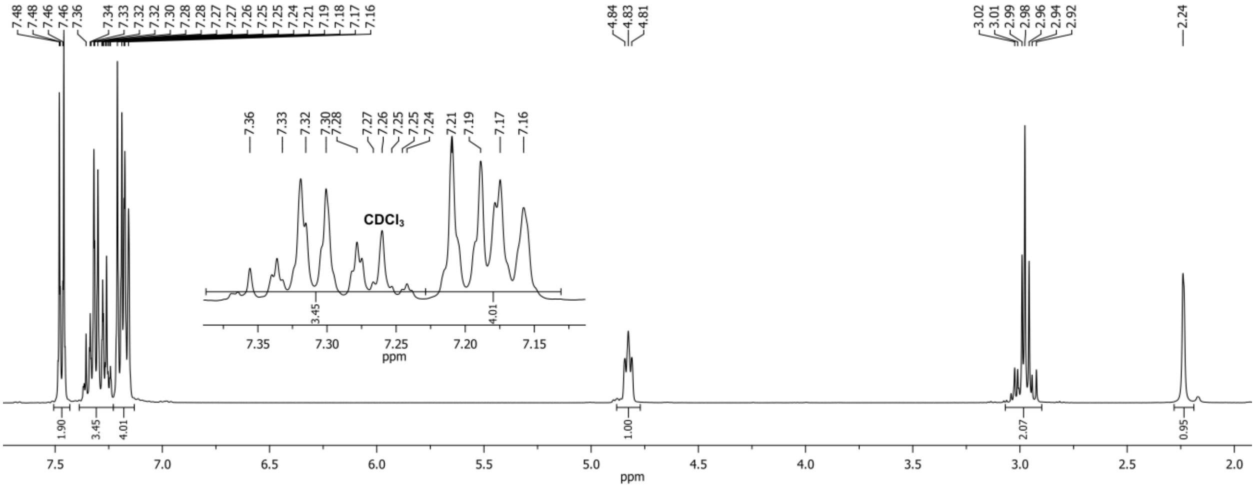

^{13}C{^{1}H} NMR

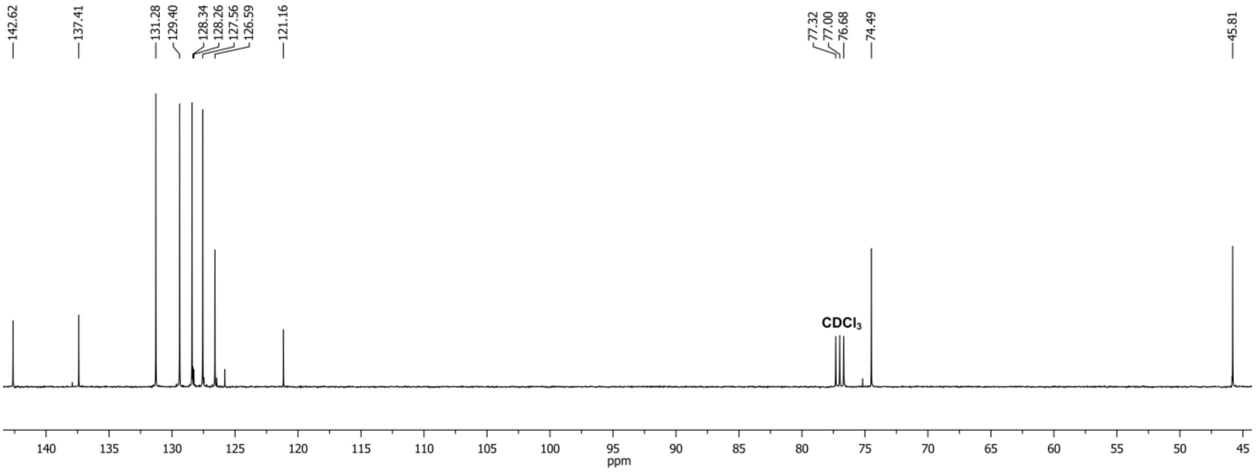

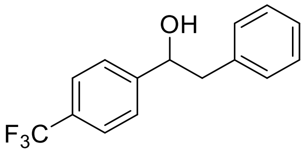

^{1}H NMR

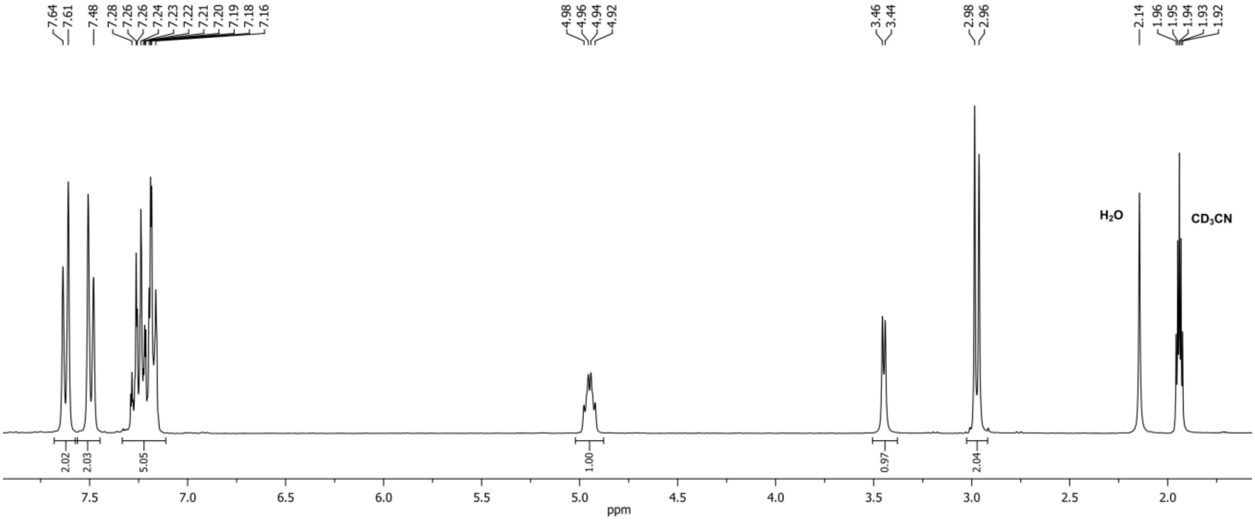

$^{13}C\{^{1}H\}$ NMR

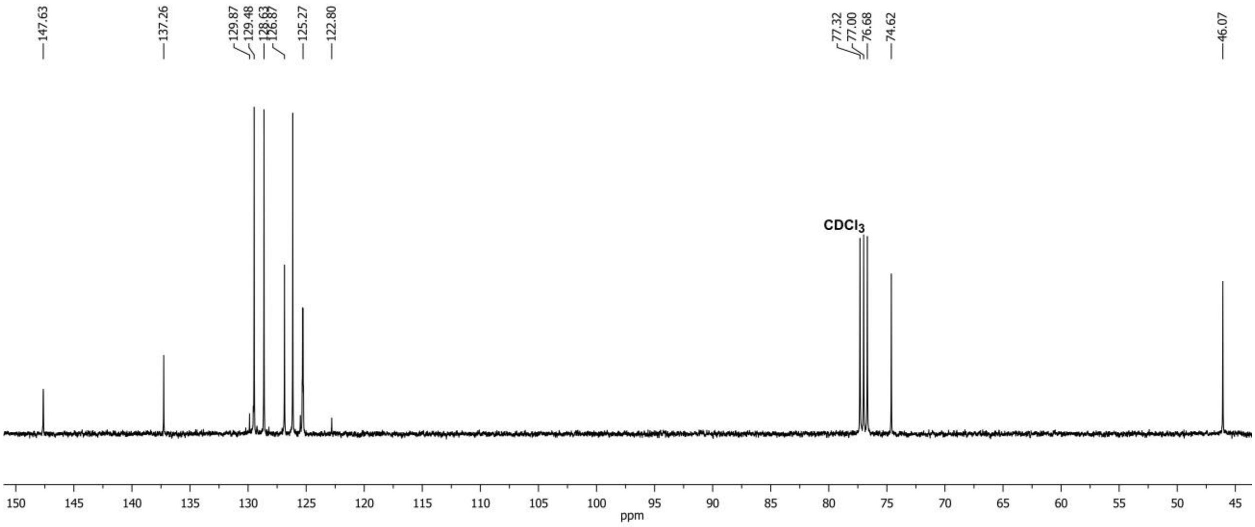

$^{19}F\{^{1}H\}$ NMR

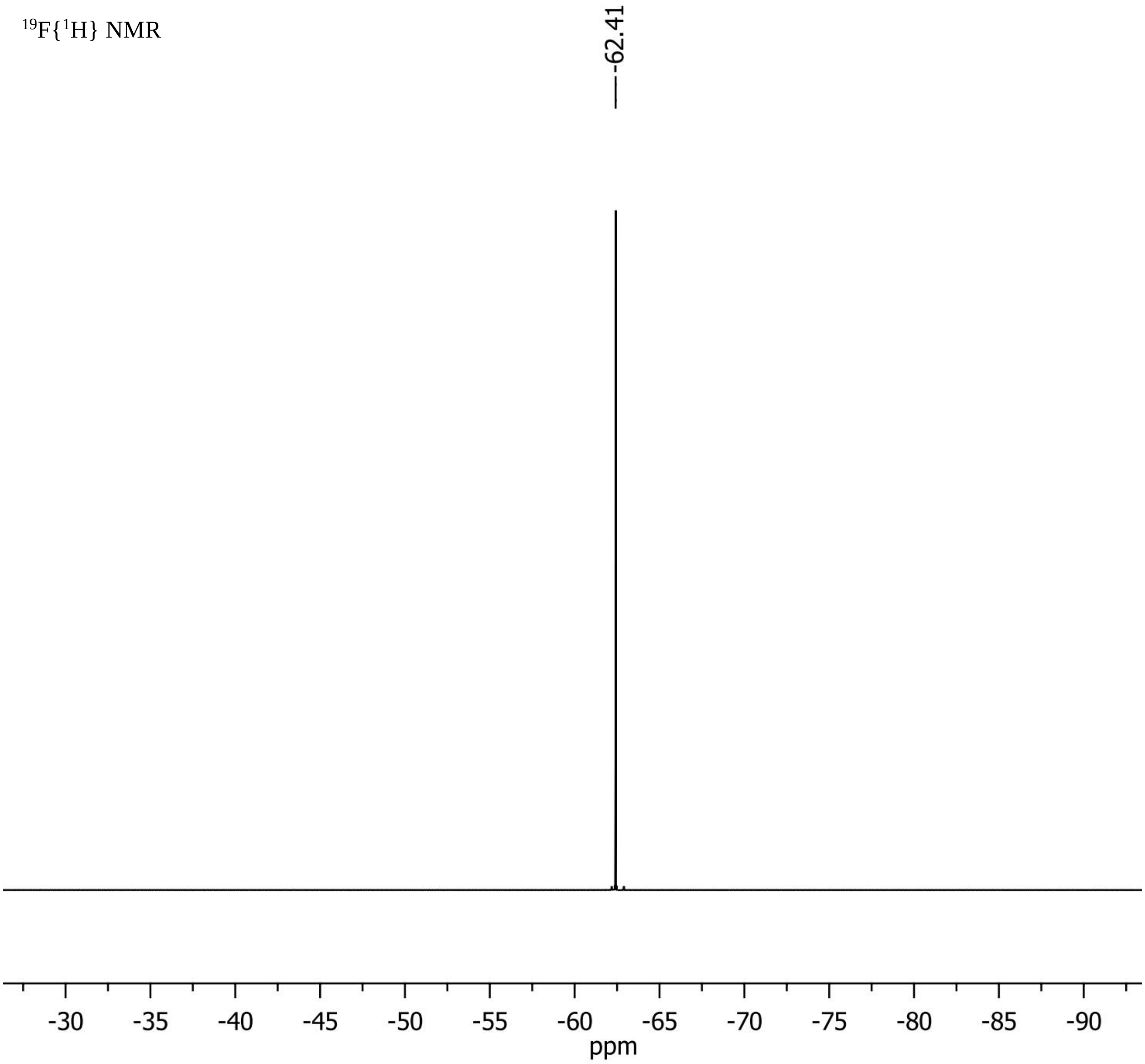

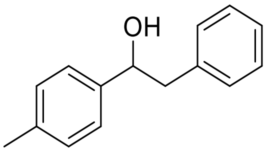

^{1}H NMR

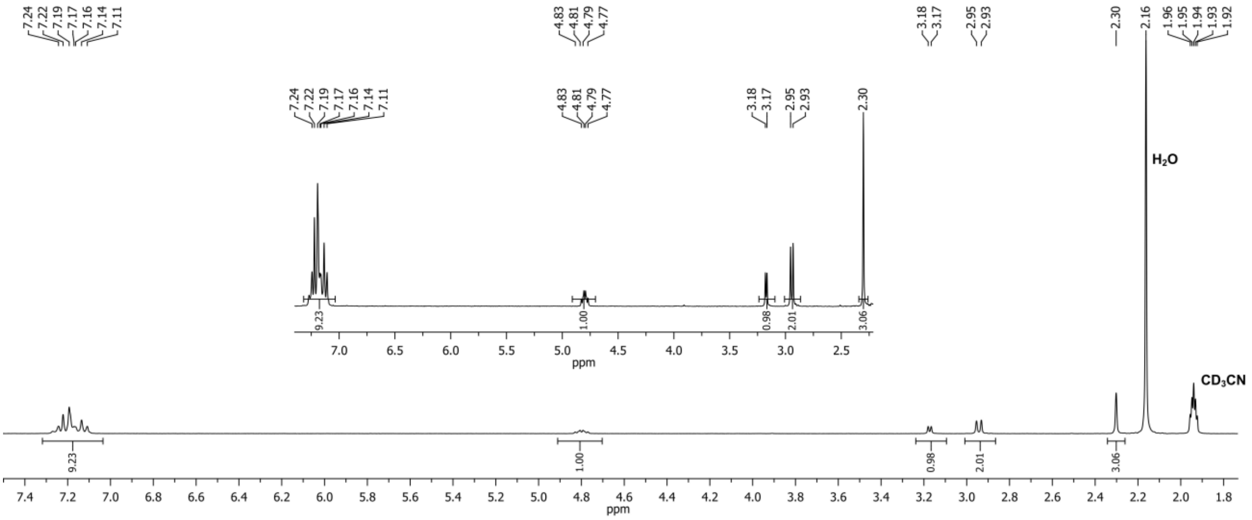

$^{13}C\{^1H\}$ NMR

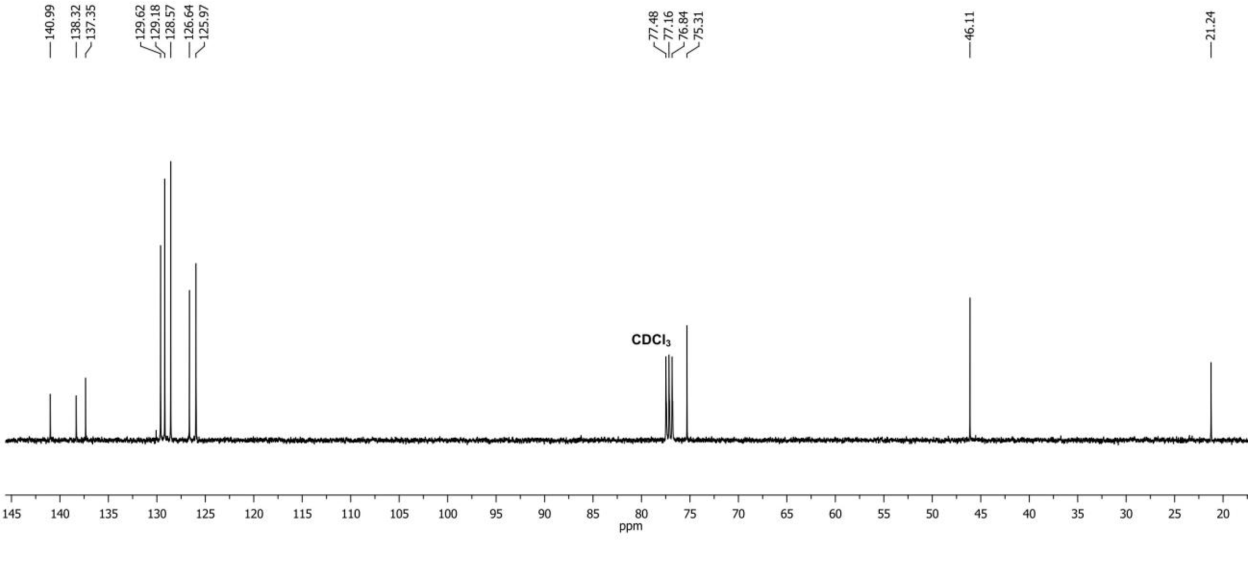

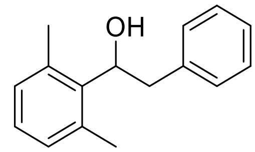

^{1}H NMR

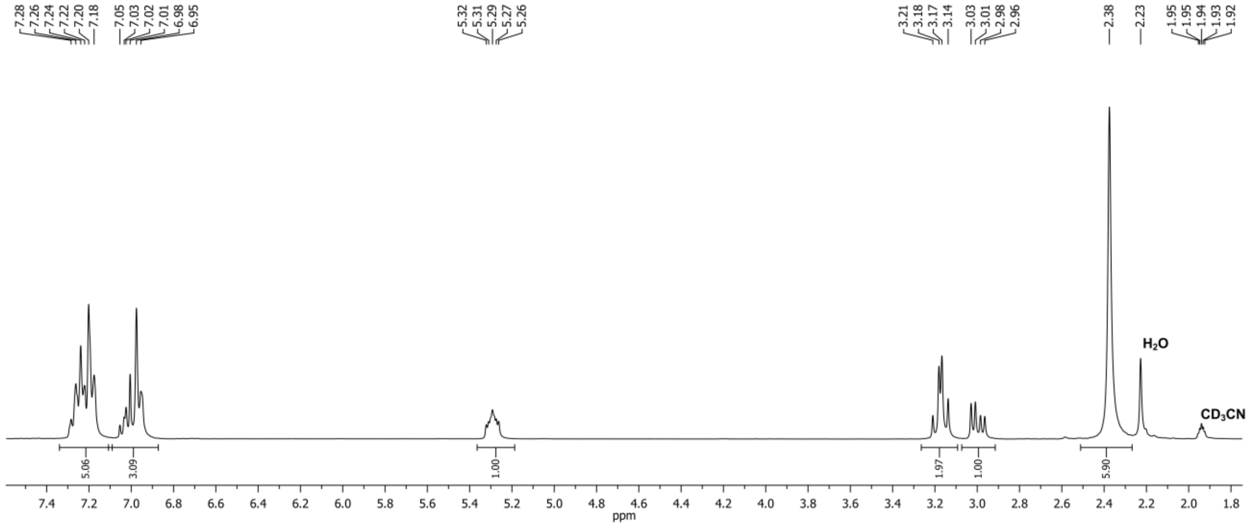

^{13}C{^{1}H} NMR

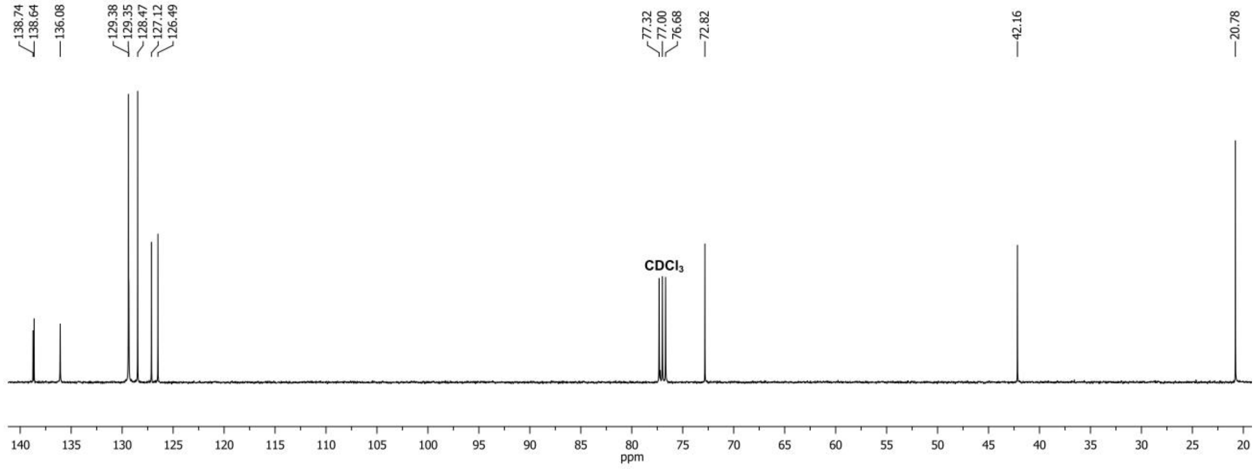

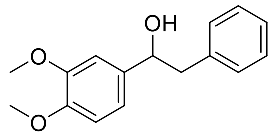

^{1}H NMR

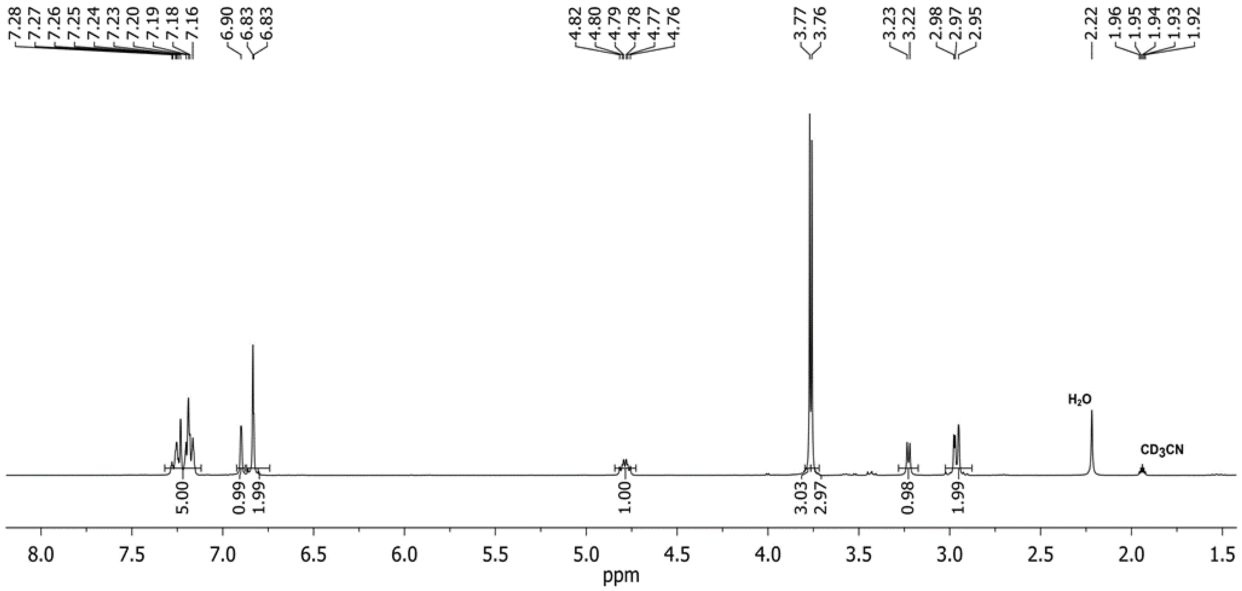

$^{13}C\{^{1}H\}$ NMR

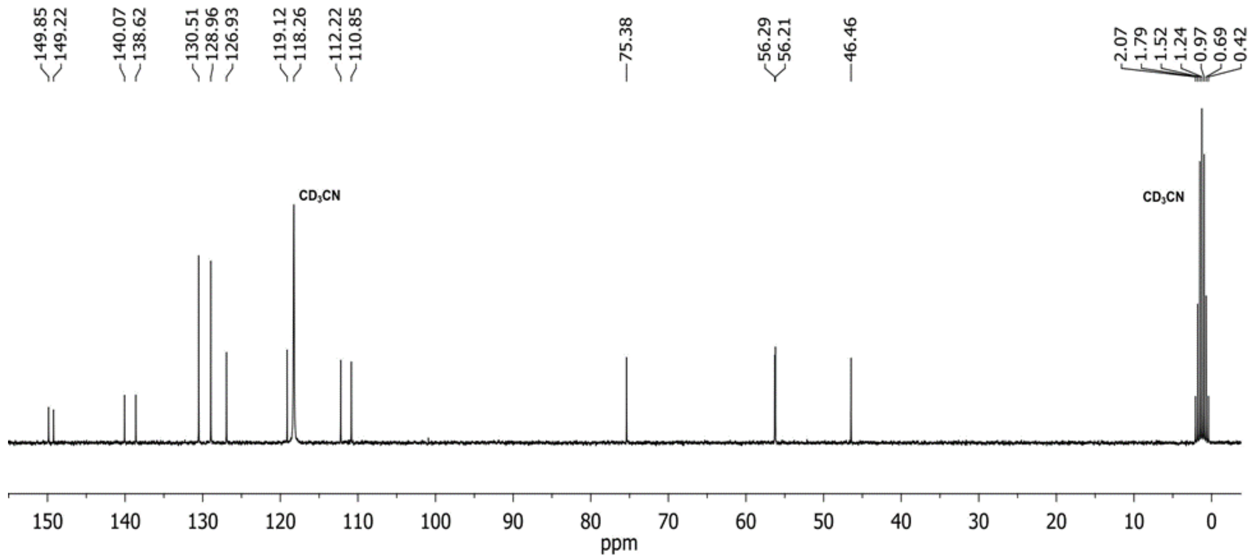

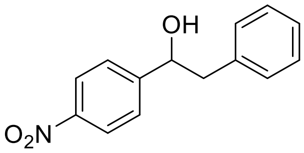

^{1}H NMR

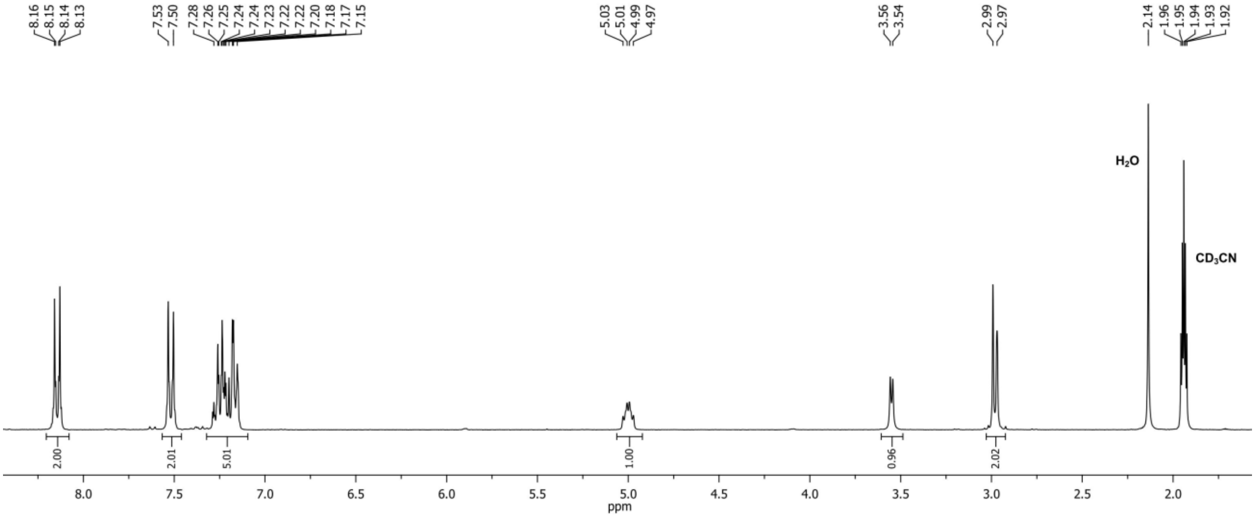

^{13}C{^{1}H} NMR

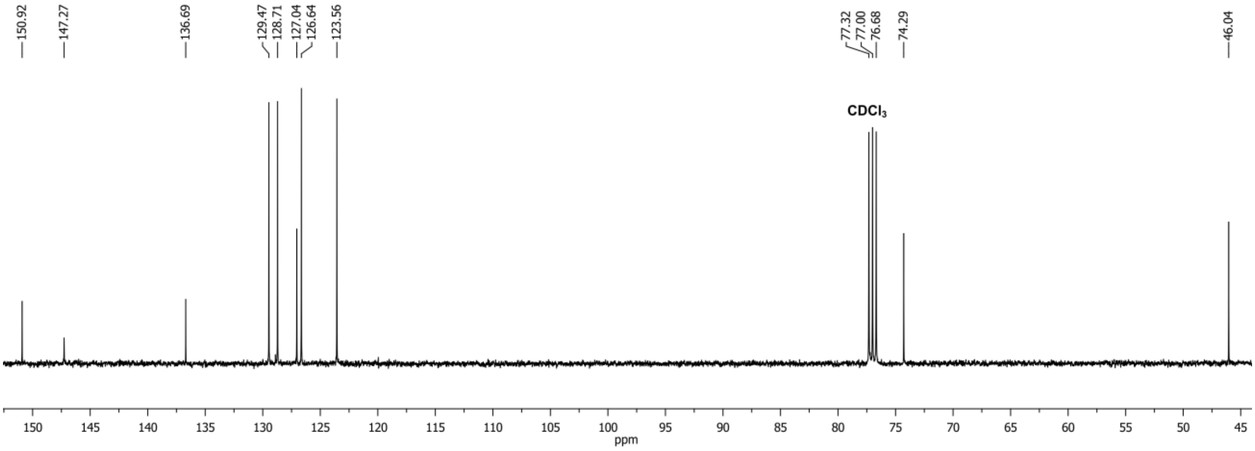

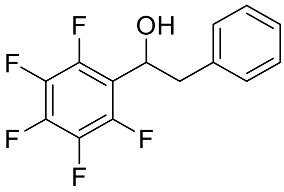

^{1}H NMR

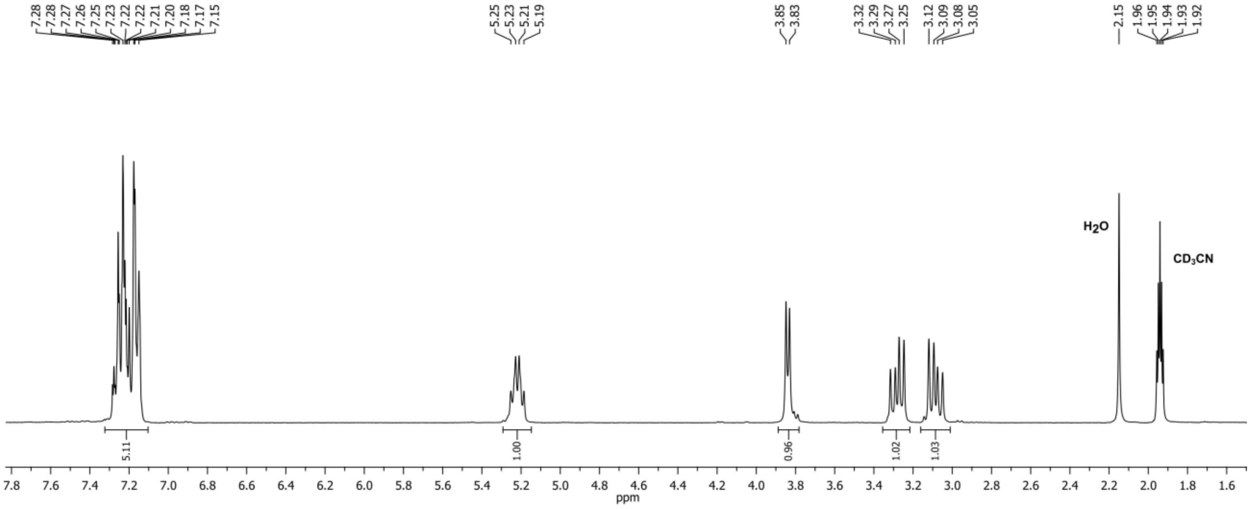

^{13}C{^{1}H} NMR

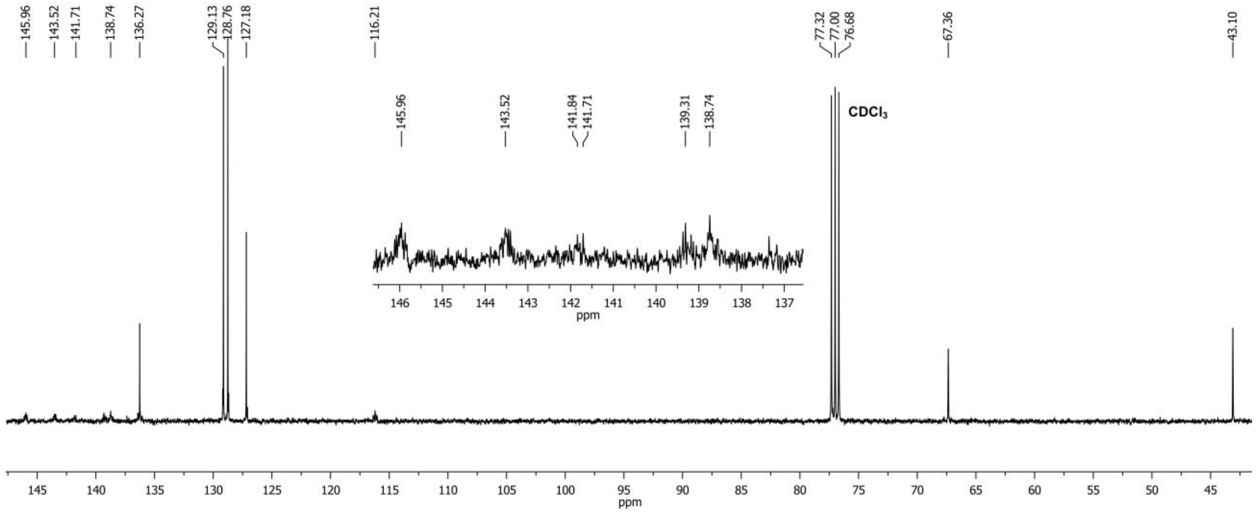

$^{19}F\{^{1}H\}$ NMR

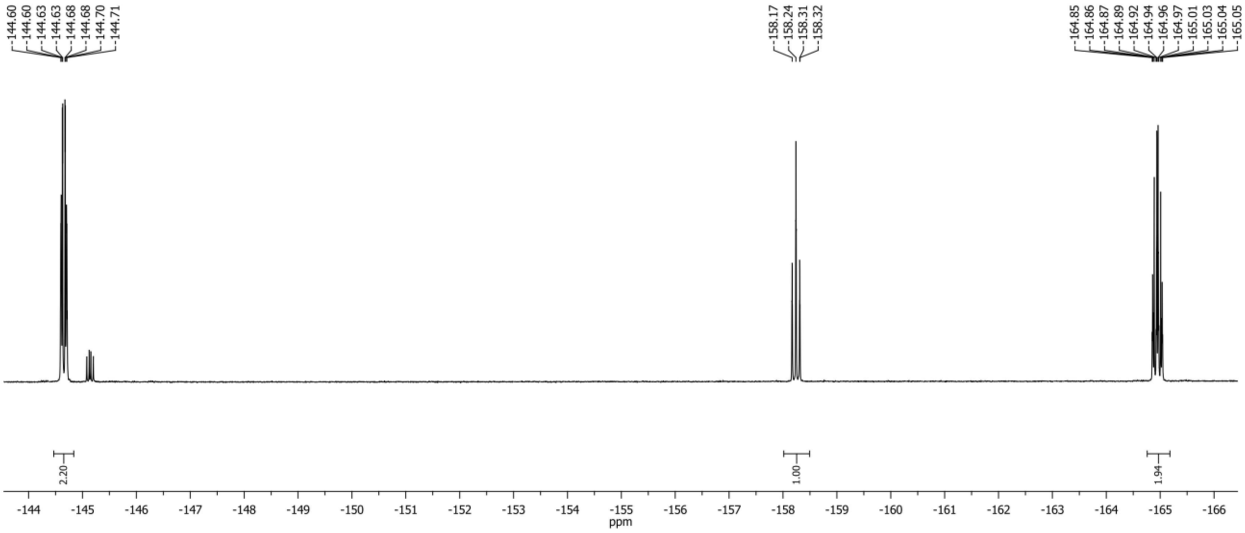
-144.60
-144.60
-144.63
-144.63
-144.68
-144.68
-144.70
-144.71
-158.17
-158.24
-158.31
-158.32
-164.85
-164.86
-164.87
-164.89
-164.92
-164.94
-164.96
-164.97
-165.01
-165.03
-165.04
-165.05
2.20
1.00
1.94
ppm

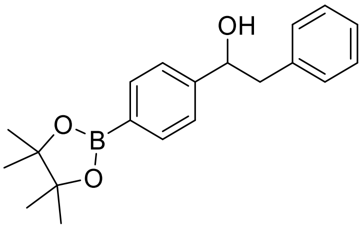

^{1}H NMR

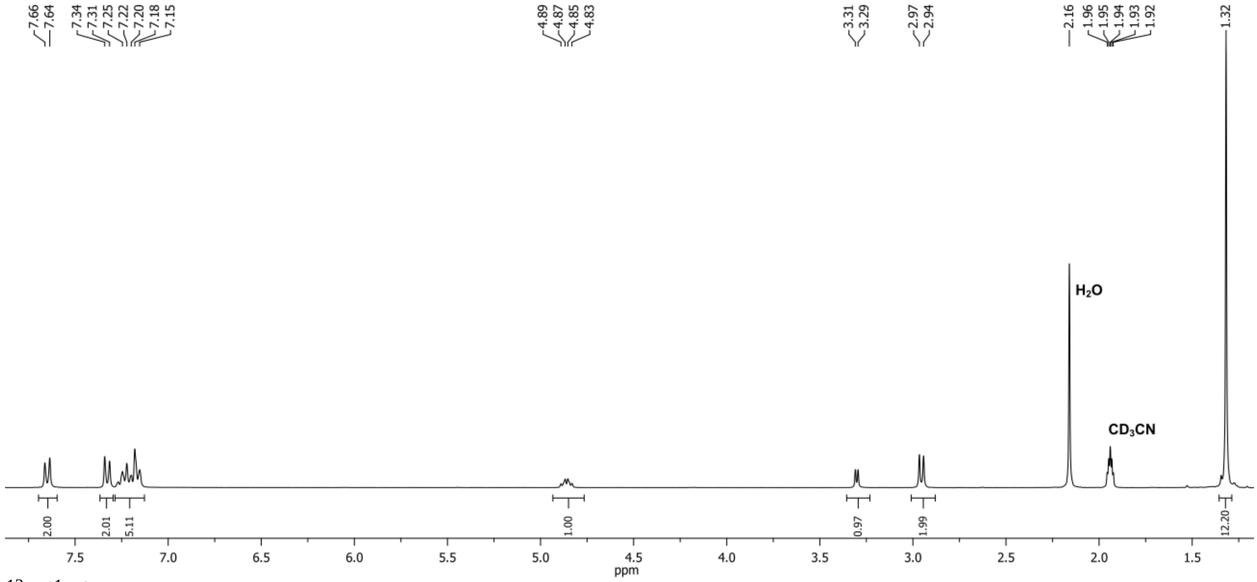

^{13}C{^{1}H} NMR

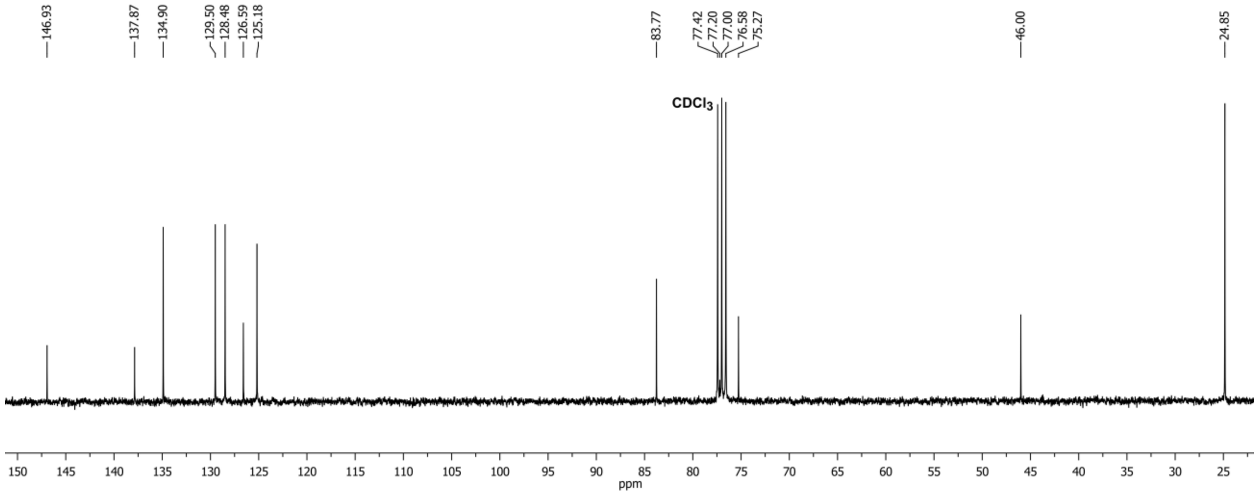

^{11}B NMR

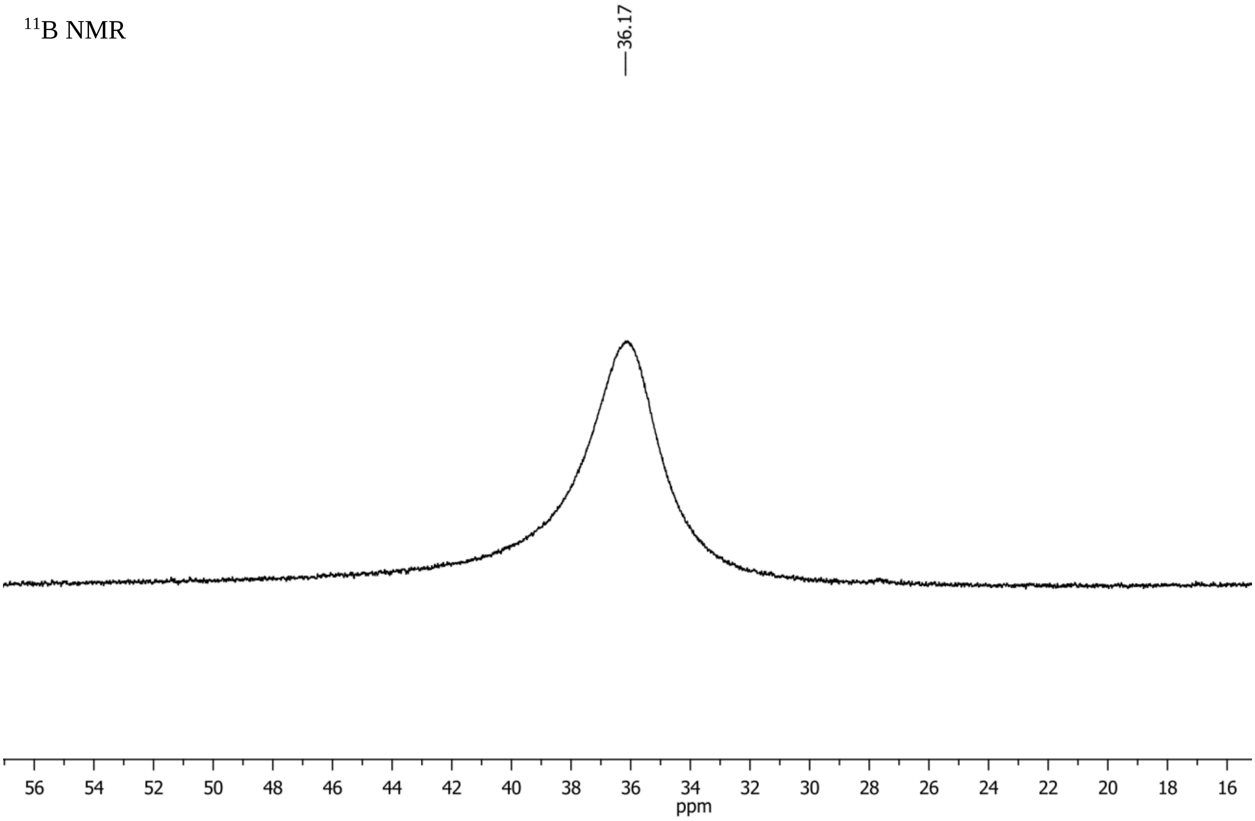

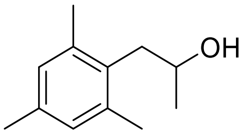

^{1}H NMR

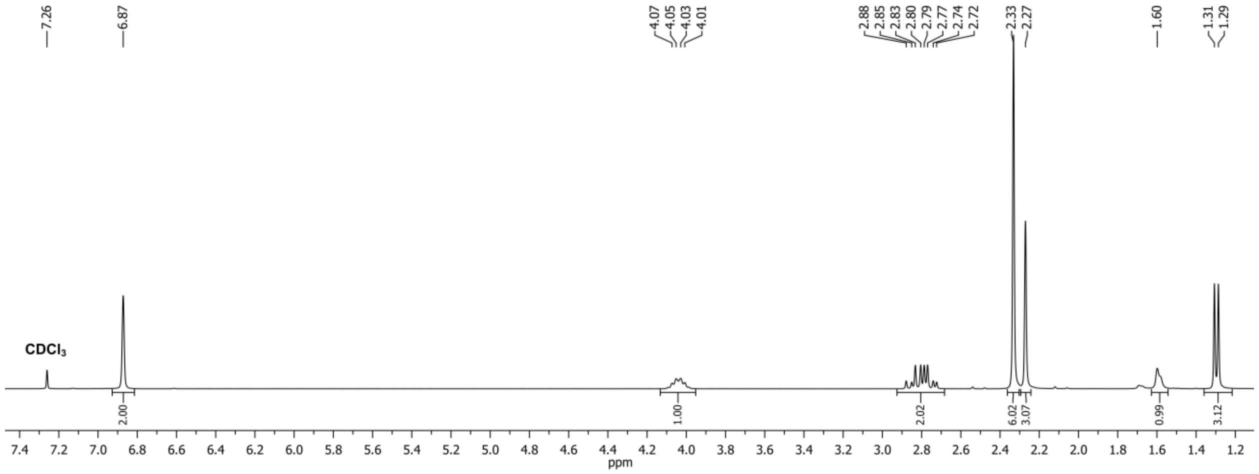

^{13}C{^{1}H} NMR

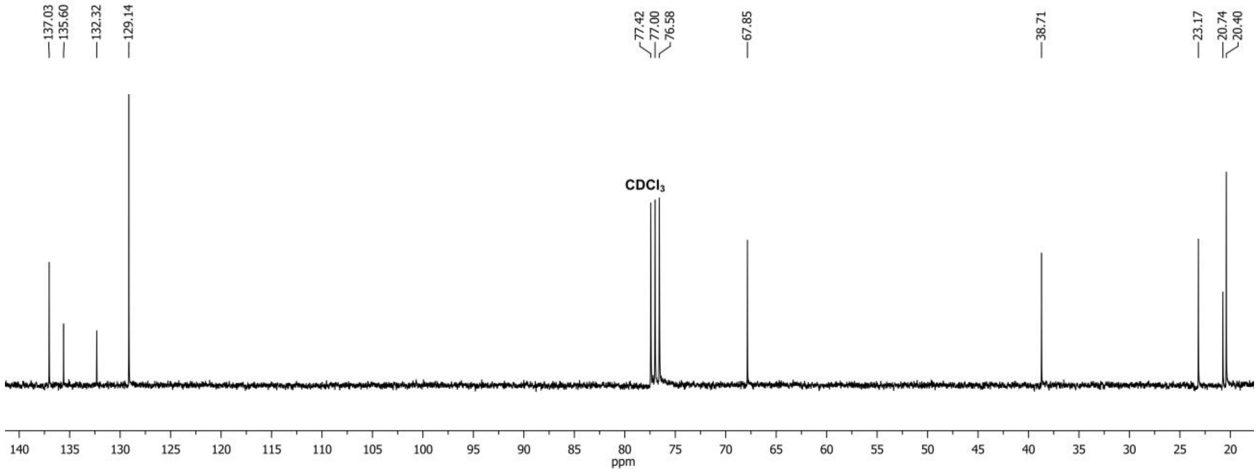

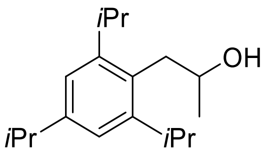

^{1}H NMR

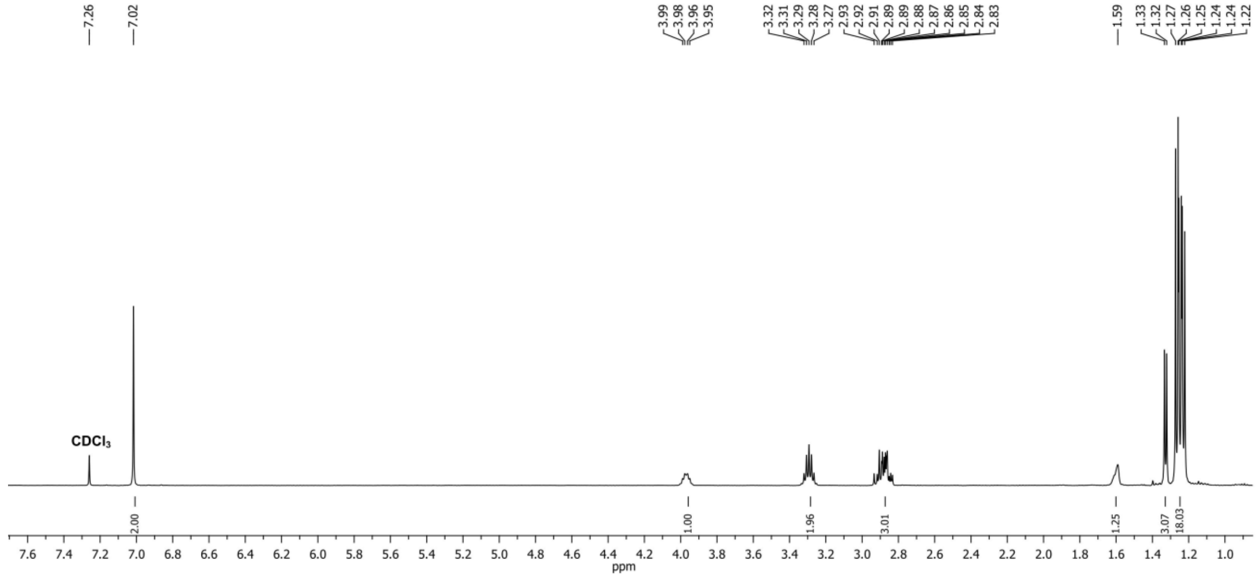

^{13}C{^{1}H} NMR

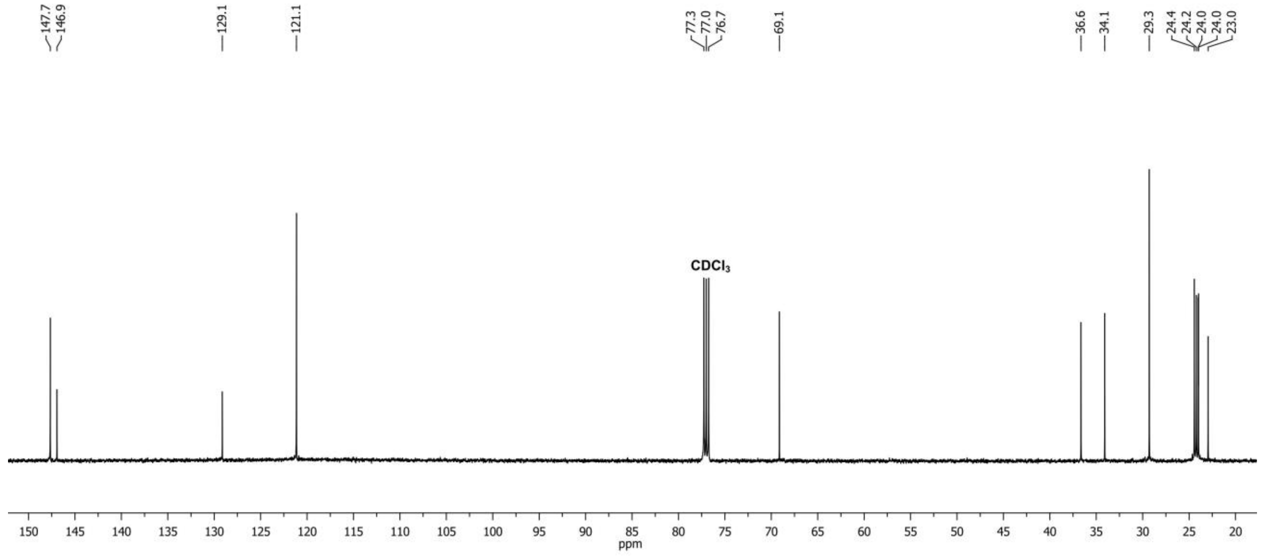

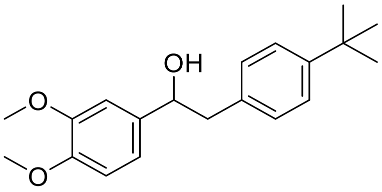

^{1}H NMR

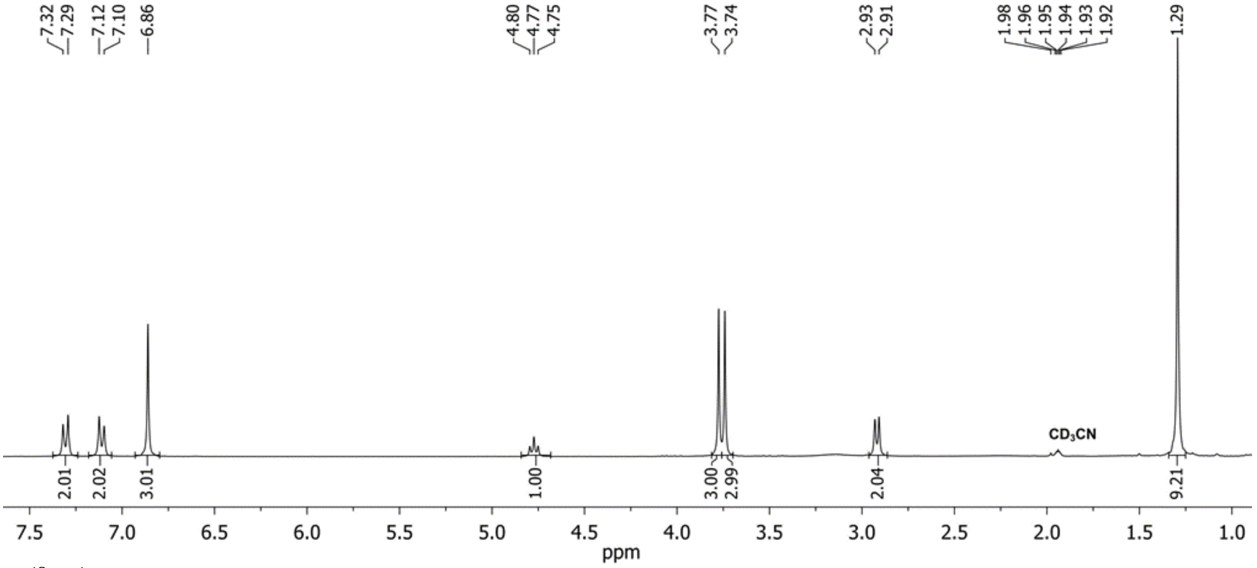

$^{13}C\{^{1}H\}$ NMR

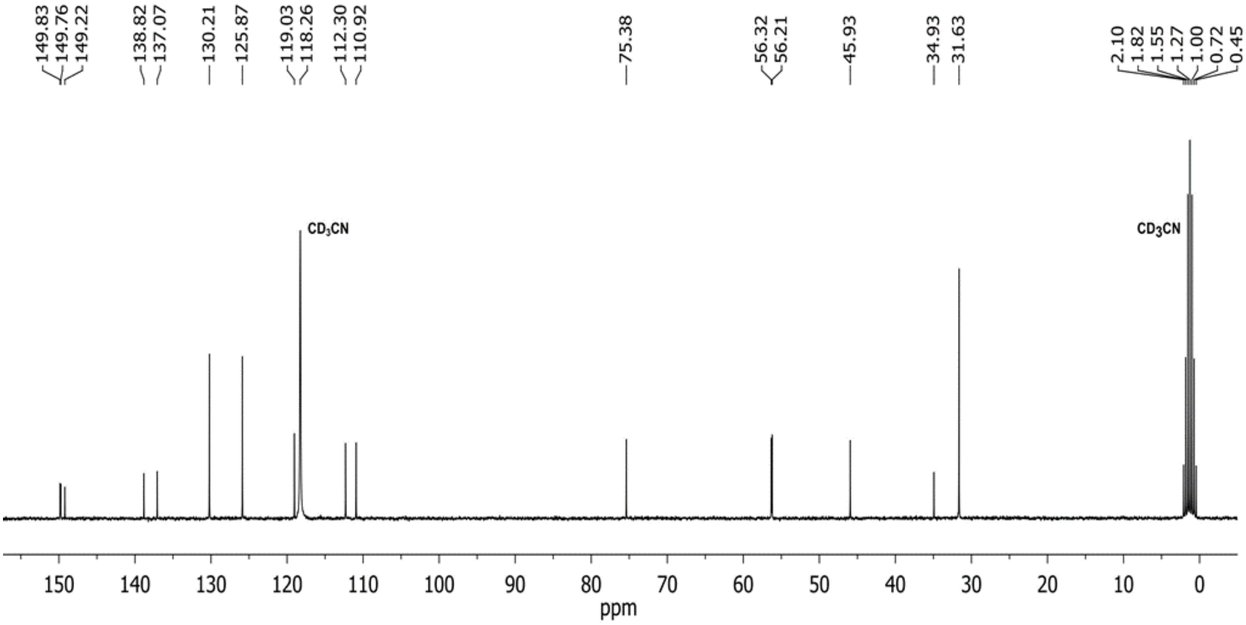

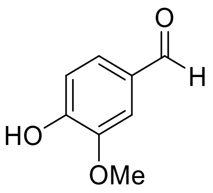

^{1}H NMR

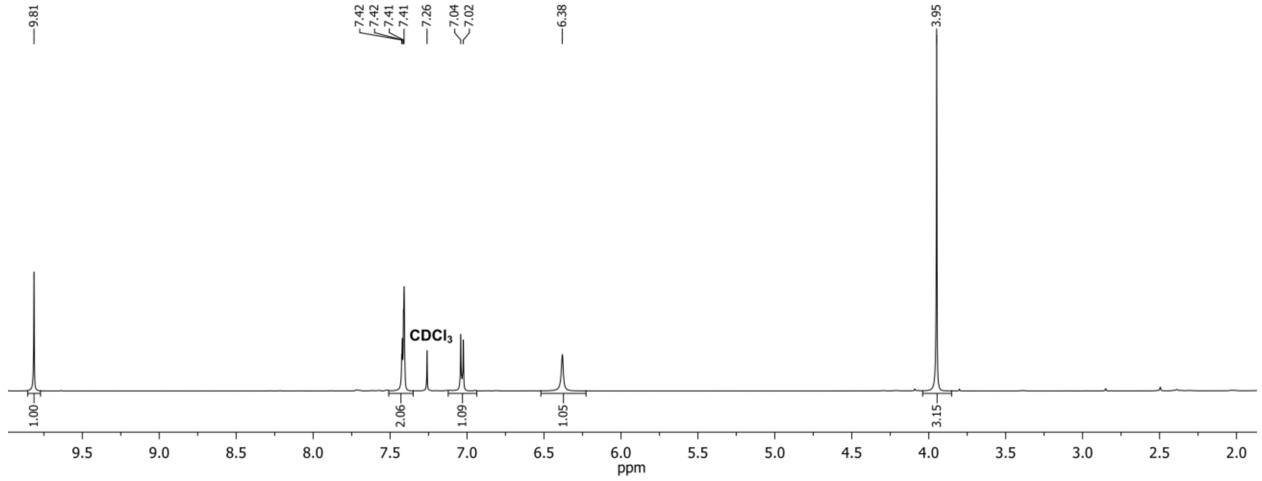

^{13}C{^{1}H} NMR

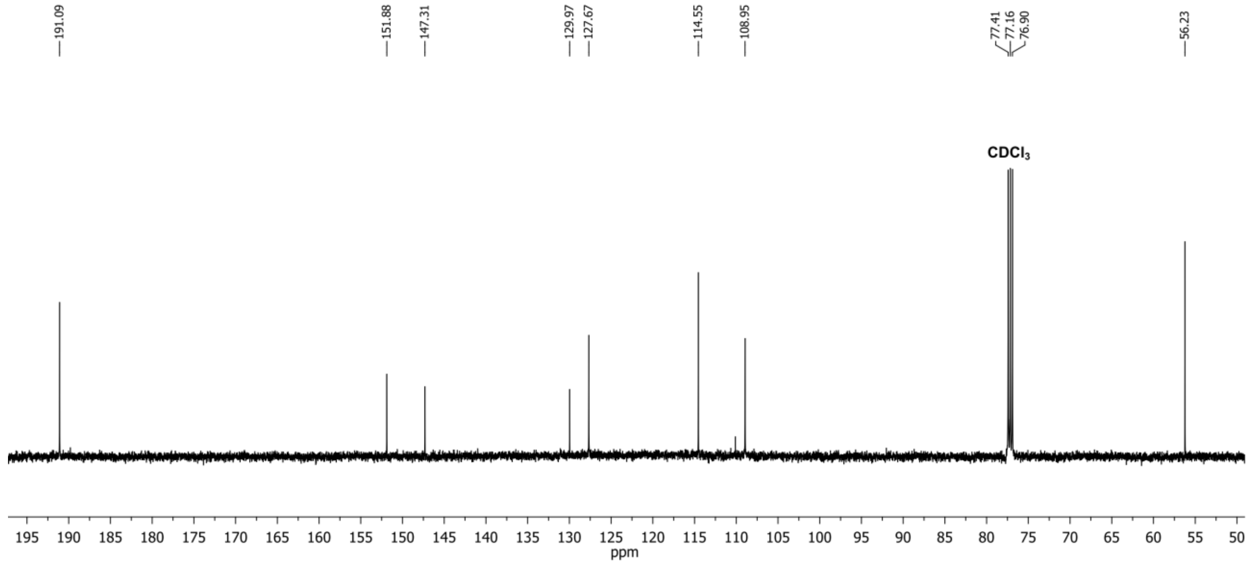

Heteronuclear Multiple Bond Correlation (HMBC) NMR

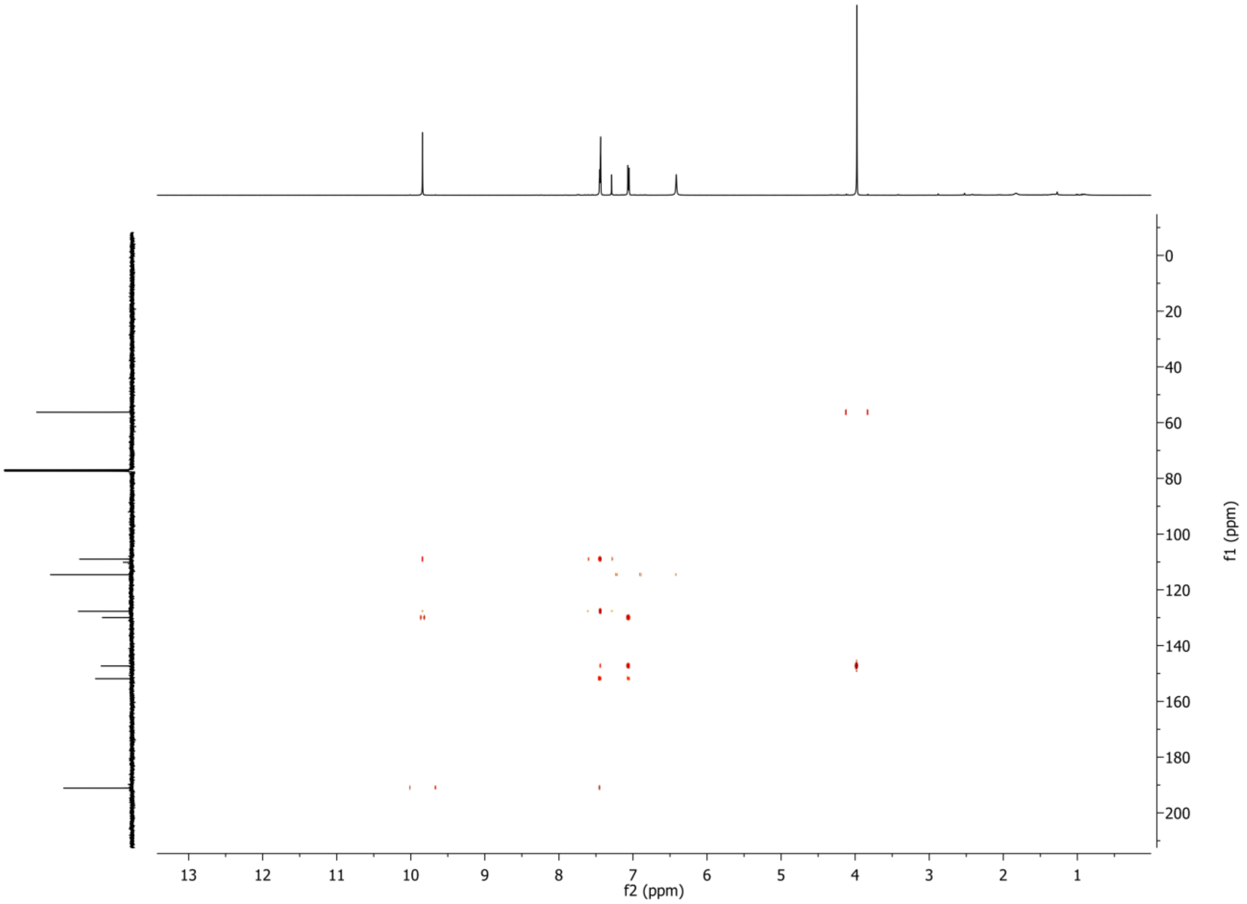

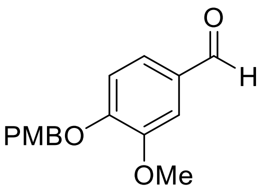

^{1}H NMR

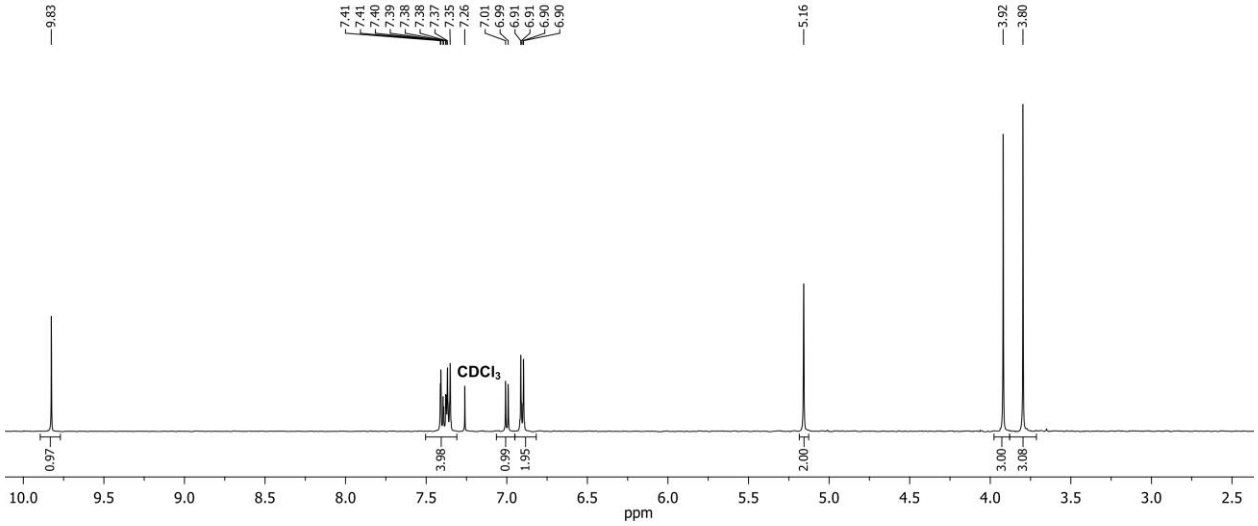

^{13}C{^{1}H} NMR

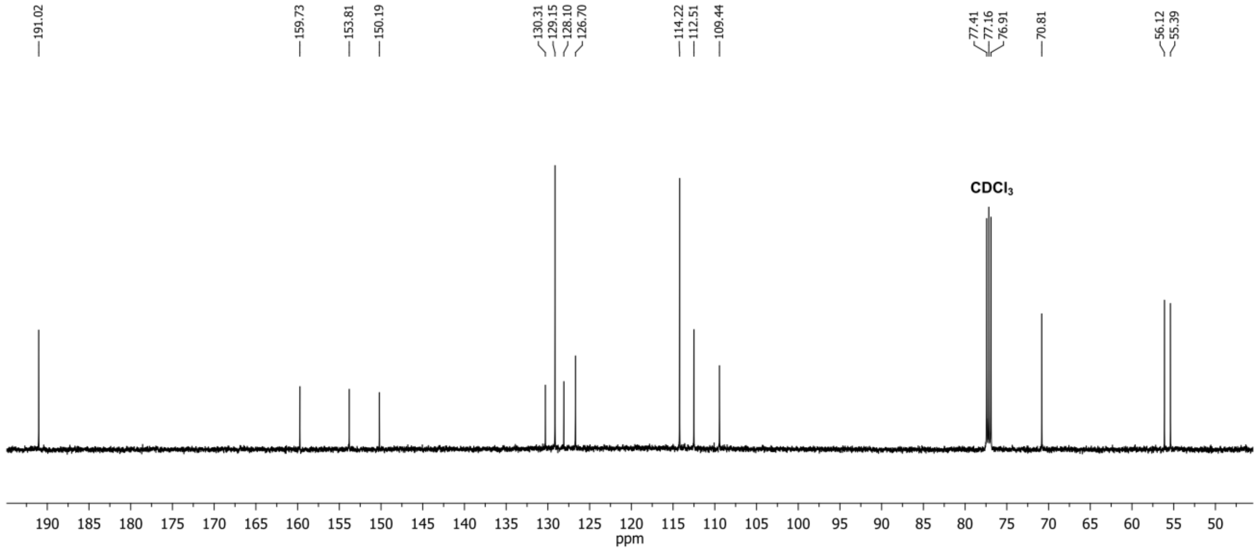

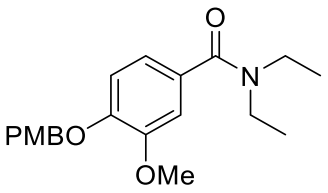

^{1}H NMR

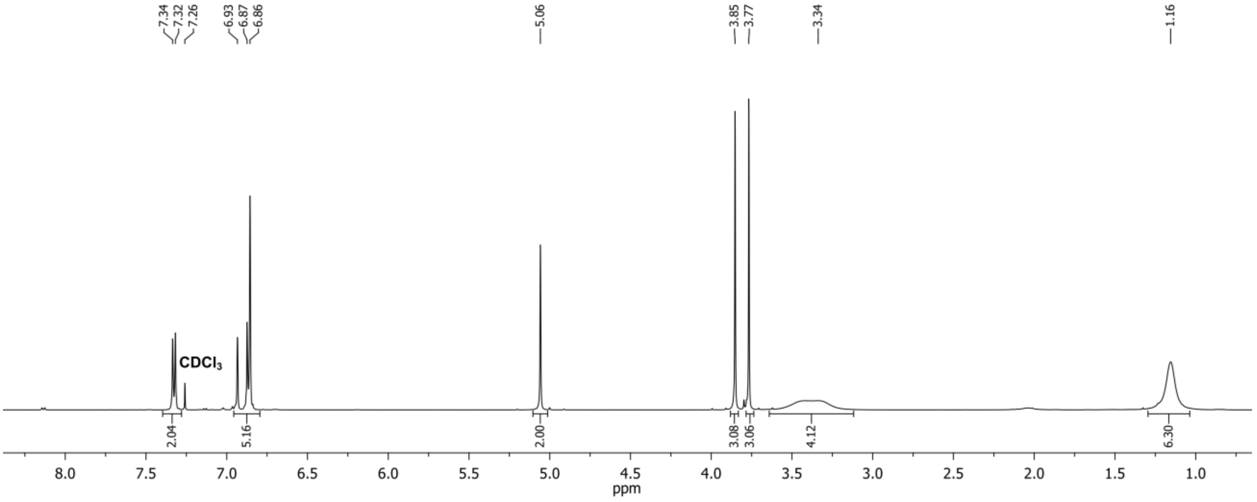

^{13}C{^{1}H} NMR

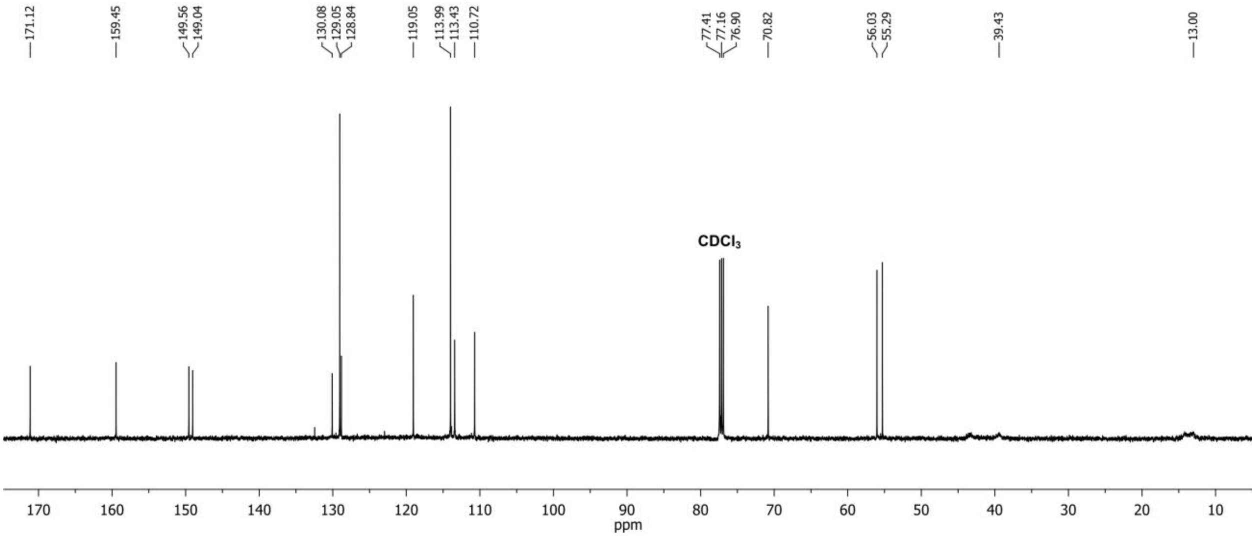

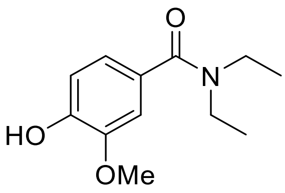

Etamivan

^{1}H NMR

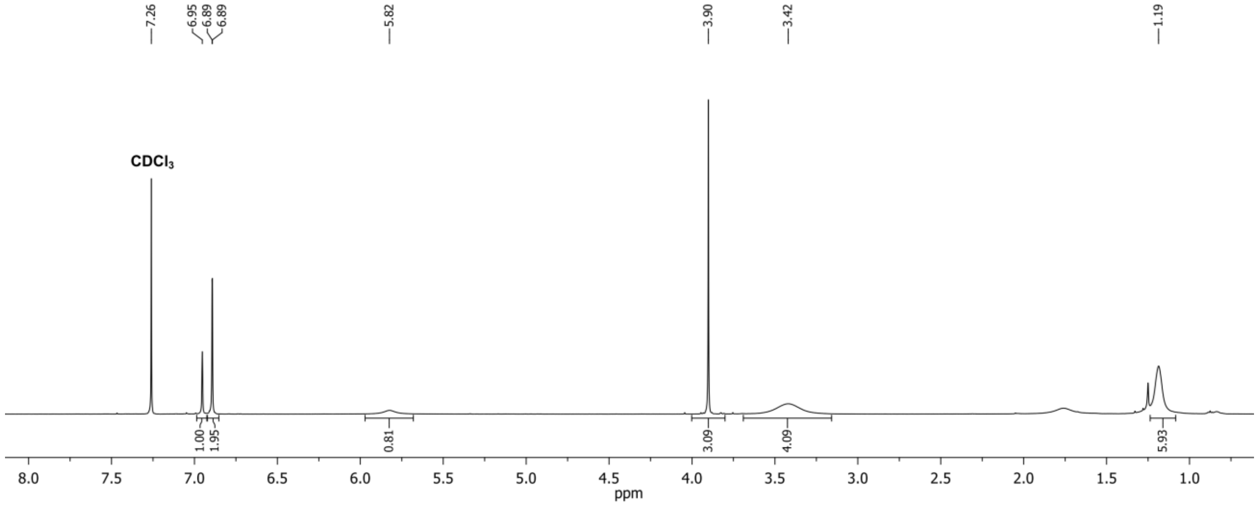

^{13}C{^{1}H} NMR

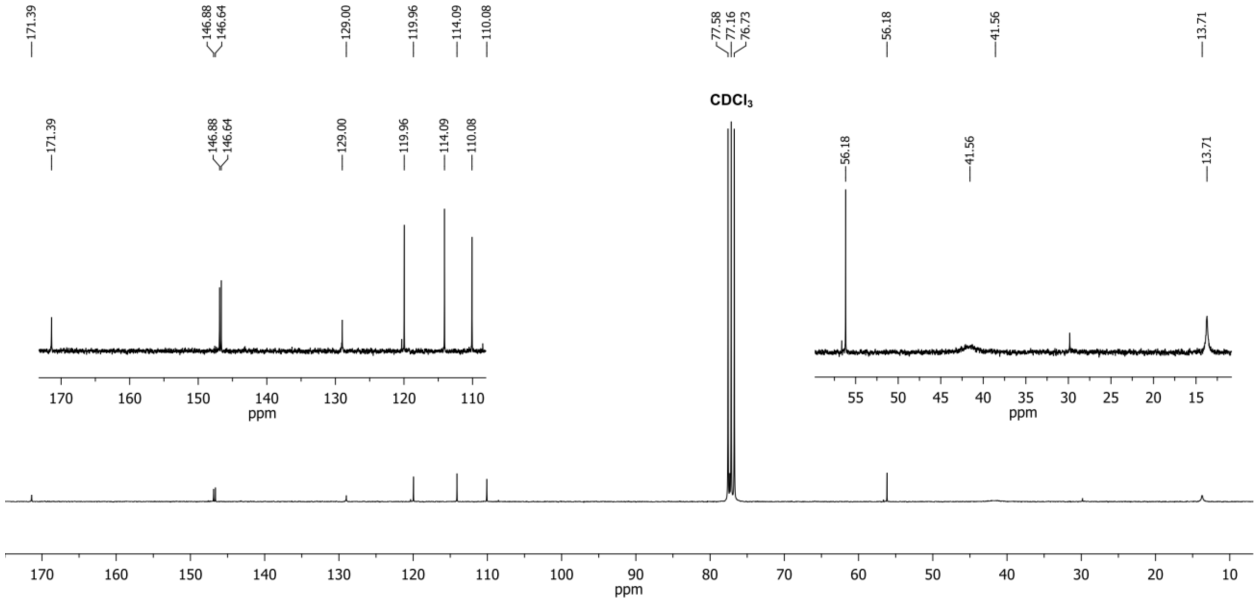

NMR spectra of reaction products

^{1}H NMR spectrum of the reaction of **3** with 1.5 eq. of 35% aq. H_2O_2 as the terminal oxidant instead of O_2.

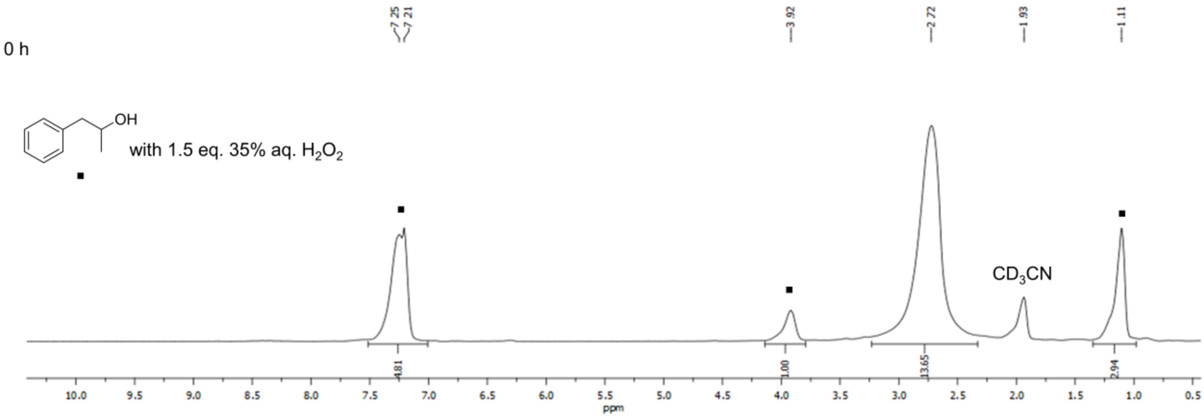

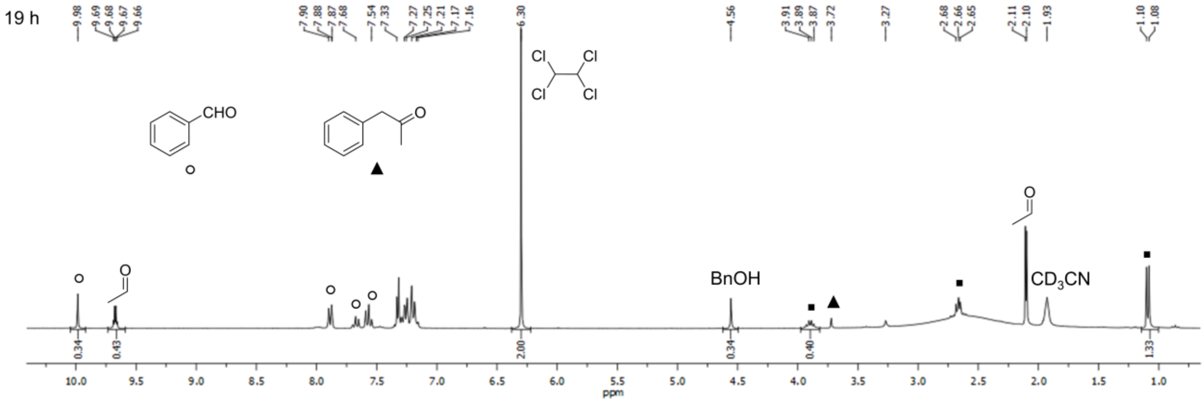

^{1}H NMR spectrum of the reaction of **3** with 1.5 eq. of 70% aq. TBHP as the terminal oxidant instead of O_2.

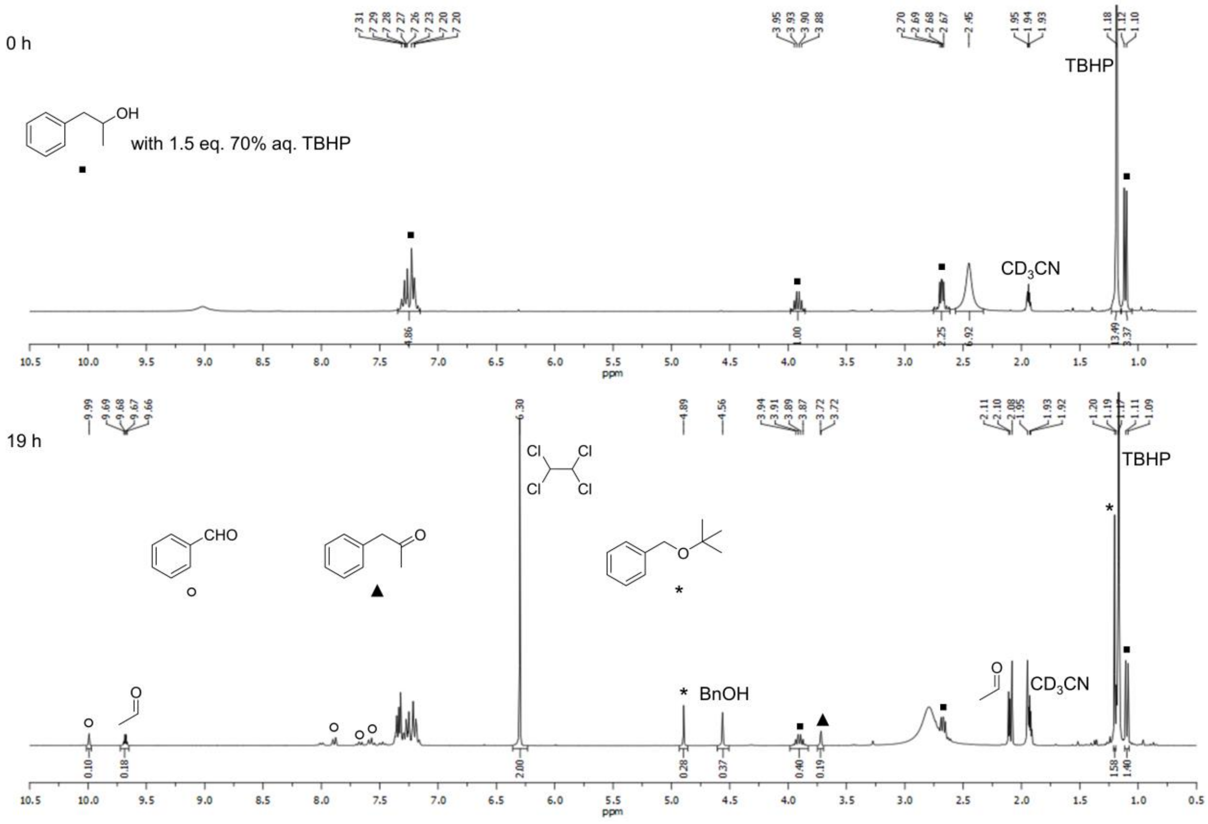

^1H NMR spectrum of the reaction of **3** with 1.5 eq. of Cu(II) triflate as the terminal oxidant instead of O_2.

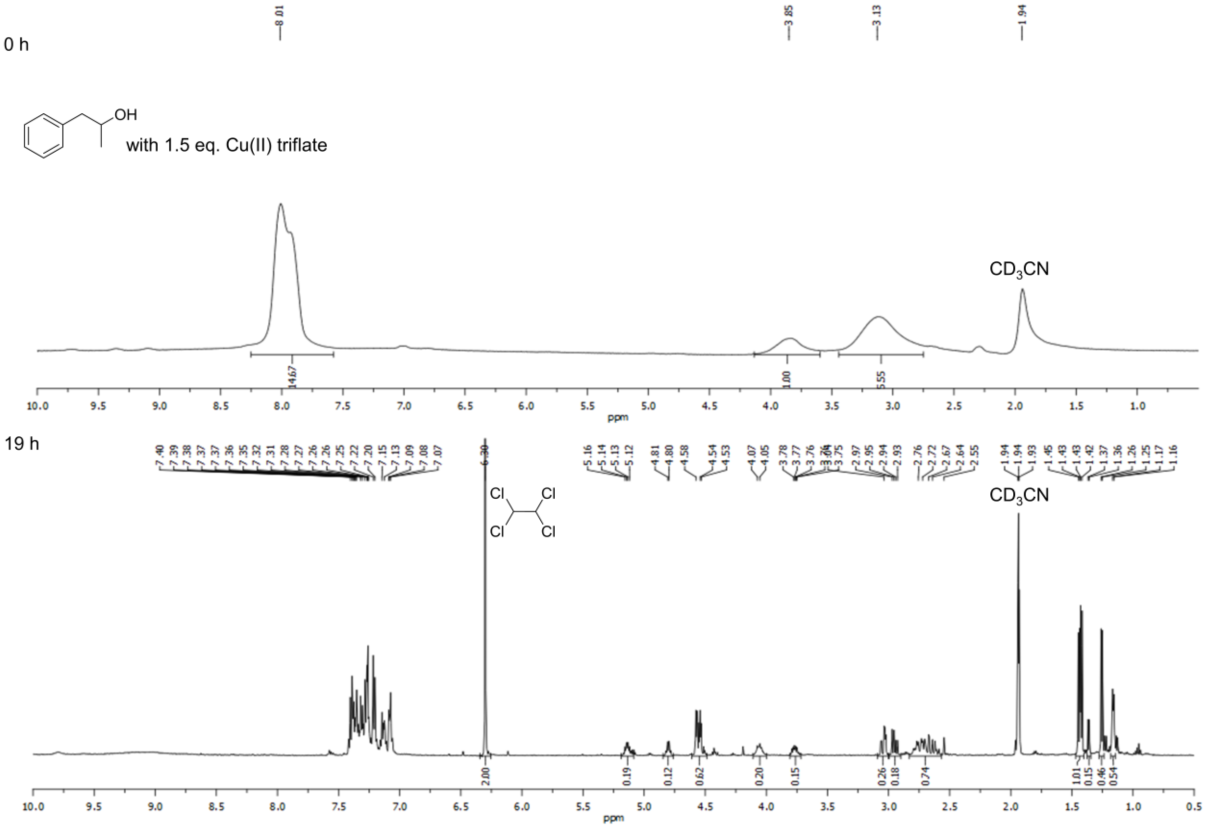

^{1}H NMR

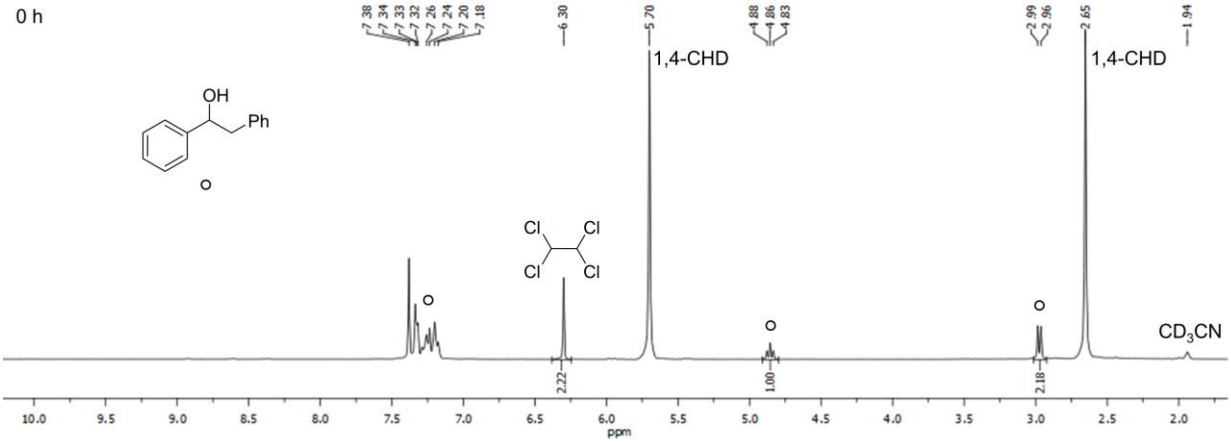
0 h
OH
Ph
1,4-CHD
1,4-CHD
Cl Cl
Cl Cl
CD3CN
2.22
1.00
2.18
ppm

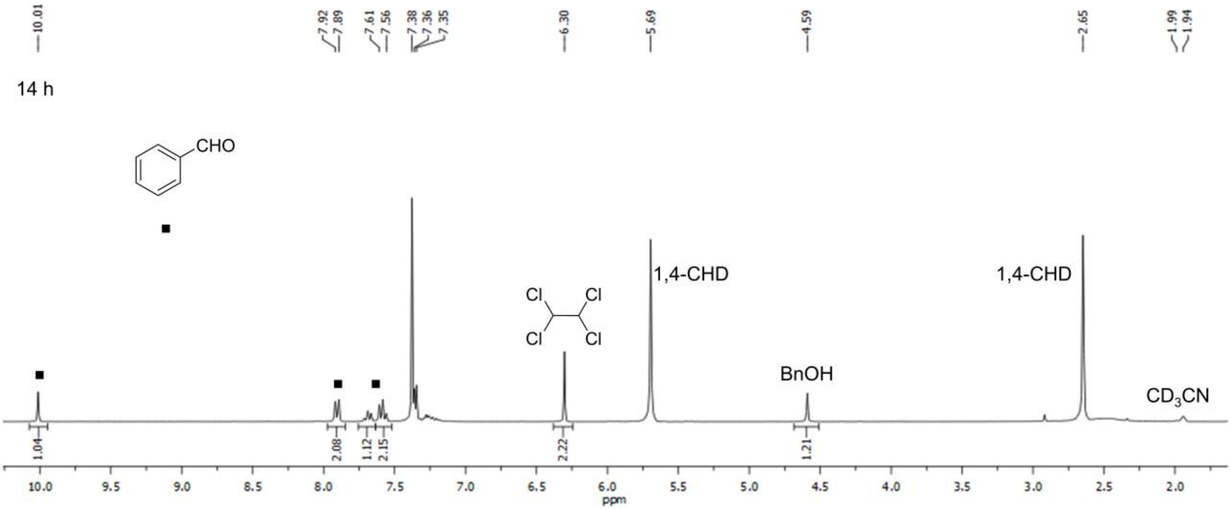
14 h
CHO
1,4-CHD
1,4-CHD
Cl Cl
Cl Cl
BnOH
CD3CN
1.04
2.08
1.12
2.15
2.22
1.21
ppm

^1H NMR spectrum of the isolated benzaldehyde.

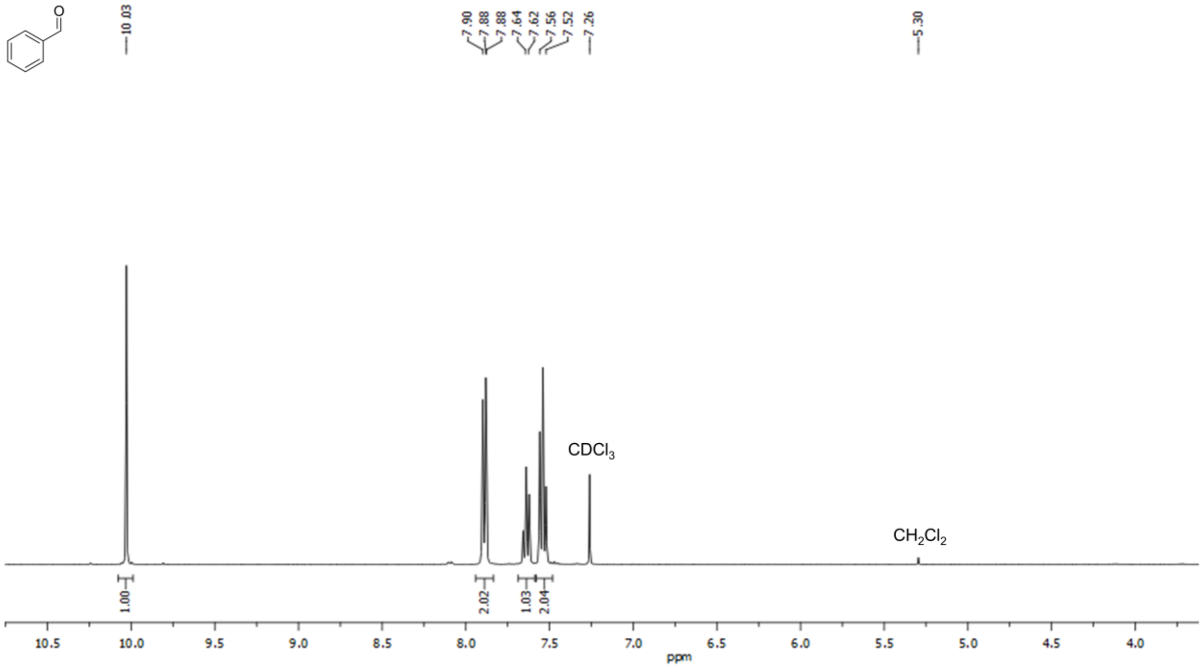

^1H NMR spectrum of the isolated benzyl alcohol.

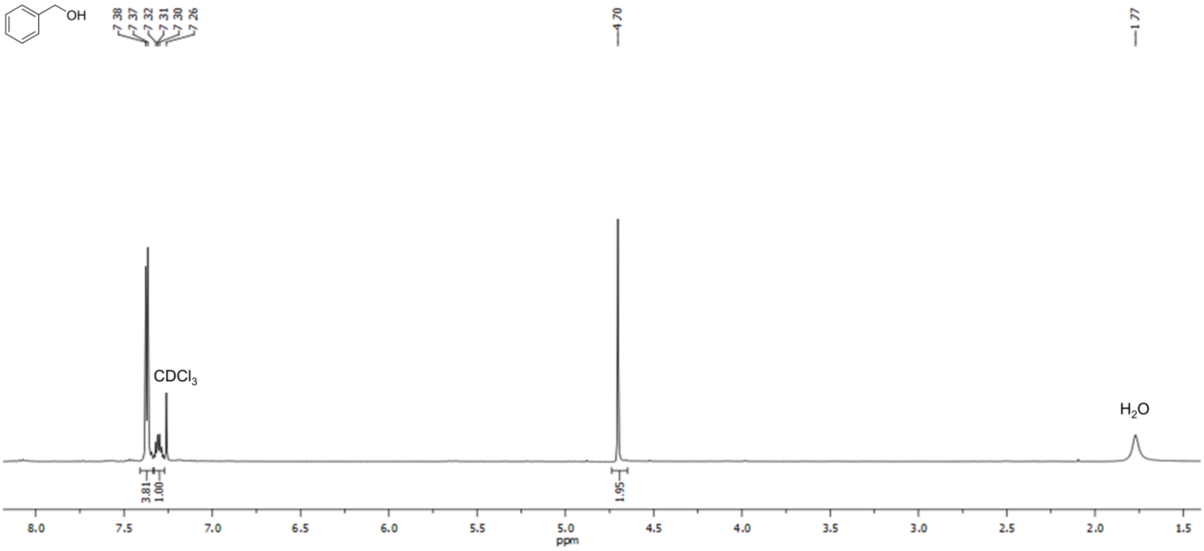

^{1}H NMR

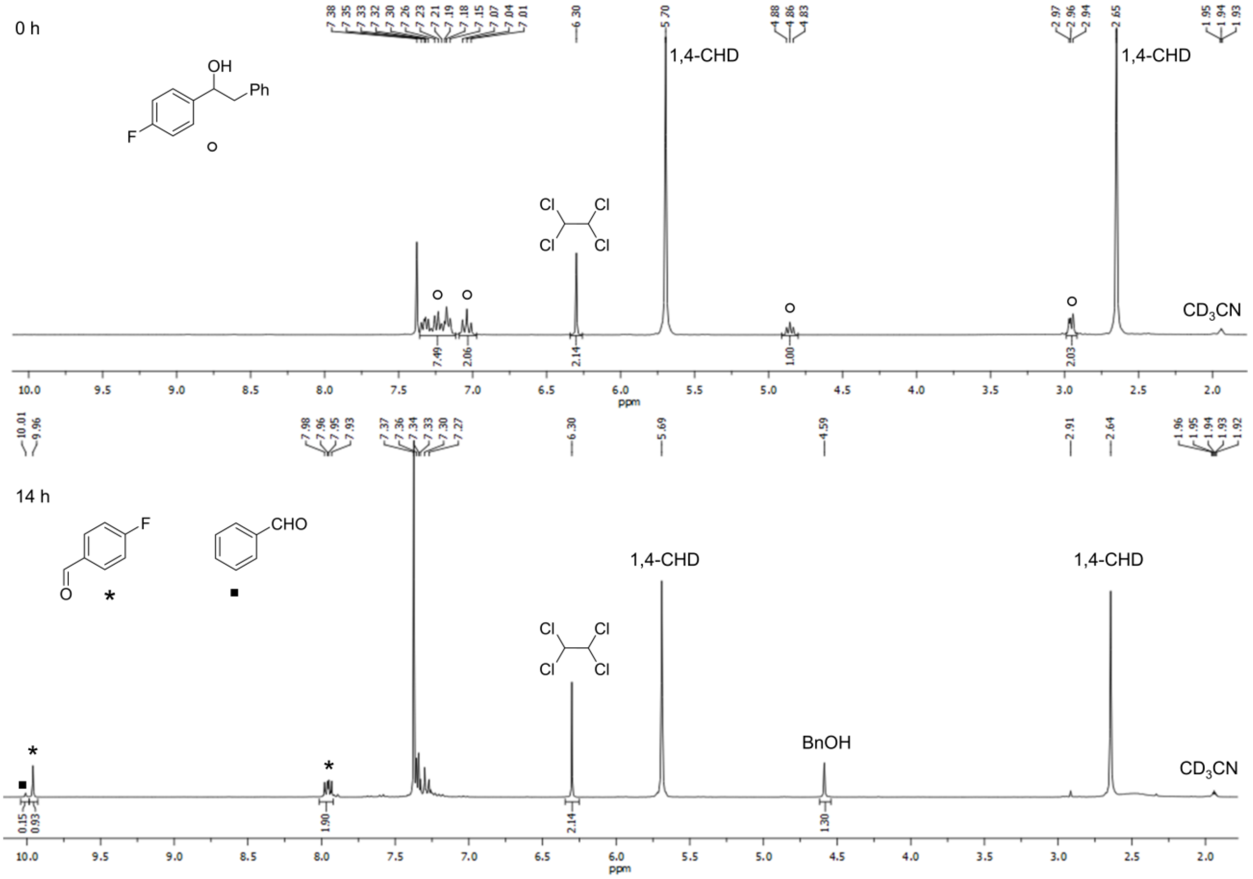
0 h
OH
Ph
F
7.38 7.35 7.33 7.32 7.30 7.26 7.23 7.21 7.19 7.18 7.15 7.07 7.04 7.01
6.30
5.70
4.88 4.86 4.83
2.97 2.96 2.94
2.65
1.95 1.94 1.93
1,4-CHD
1,4-CHD
Cl Cl Cl Cl
CD3CN
7.49 2.06 2.14 1.00 2.03
ppm
10.01 9.96
7.98 7.96 7.95 7.93
7.37 7.36 7.34 7.33 7.30 7.27
6.30
5.69
4.59
2.91
2.64
1.96 1.95 1.94 1.93 1.92
14 h
F
O
CHO
1,4-CHD
1,4-CHD
Cl Cl Cl Cl
BnOH
CD3CN
0.15 0.93 1.90 2.14 1.30
ppm

^{1}H NMR

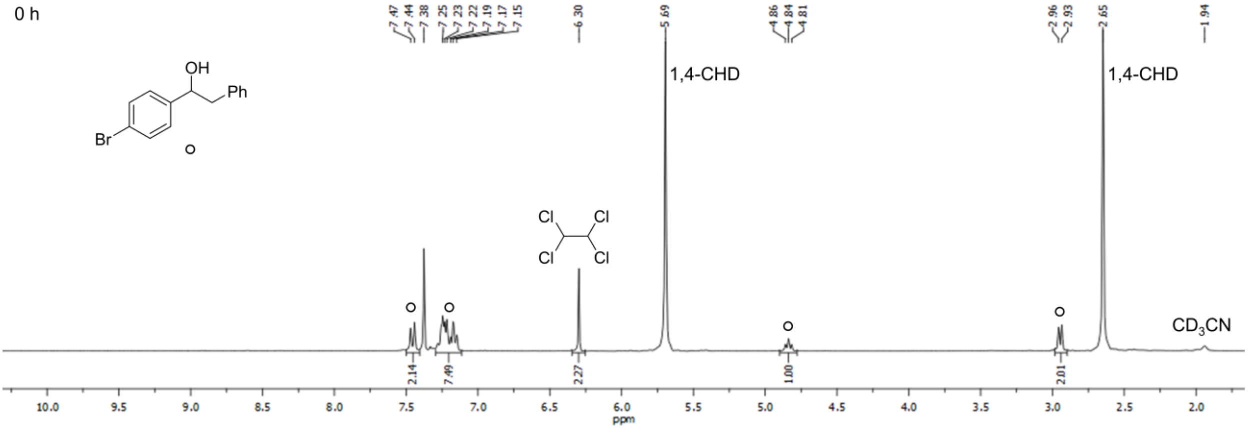
0 h
OH
Ph
Br
1,4-CHD
1,4-CHD
Cl
Cl
Cl
Cl
CD$_3$CN
2.14
7.49
2.27
1.00
2.01
ppm

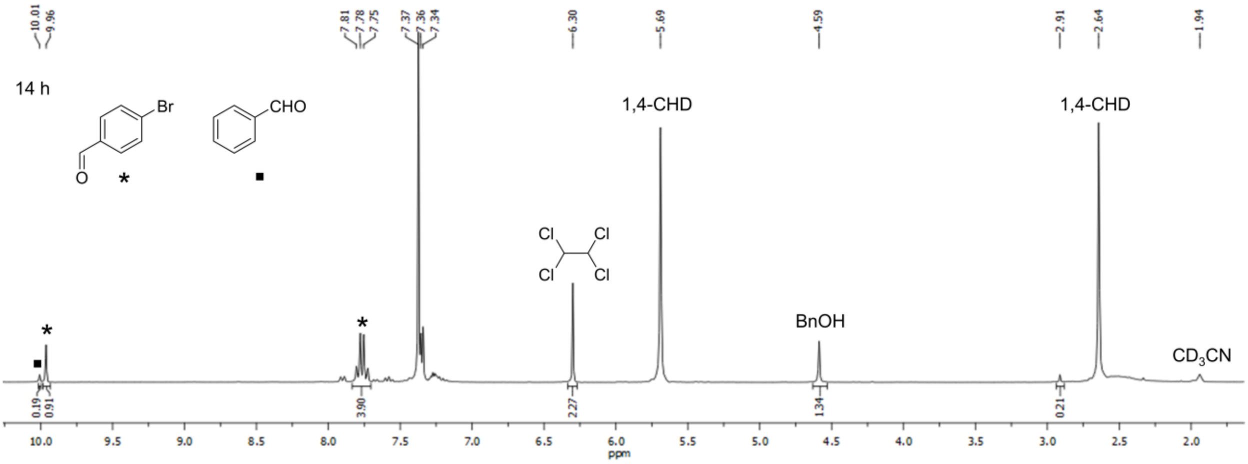
14 h
Br
CHO
O
1,4-CHD
1,4-CHD
Cl
Cl
Cl
Cl
BnOH
CD$_3$CN
0.19
0.91
3.90
2.27
1.34
0.21
ppm

^{1}H NMR

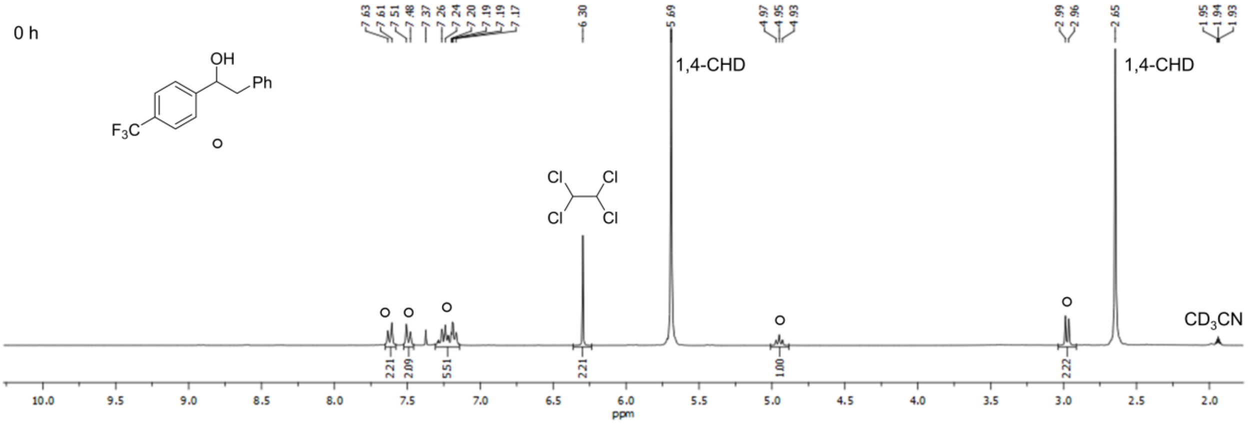
0 h
OH
Ph
F3C
1,4-CHD
1,4-CHD
Cl Cl
Cl Cl
CD3CN
ppm

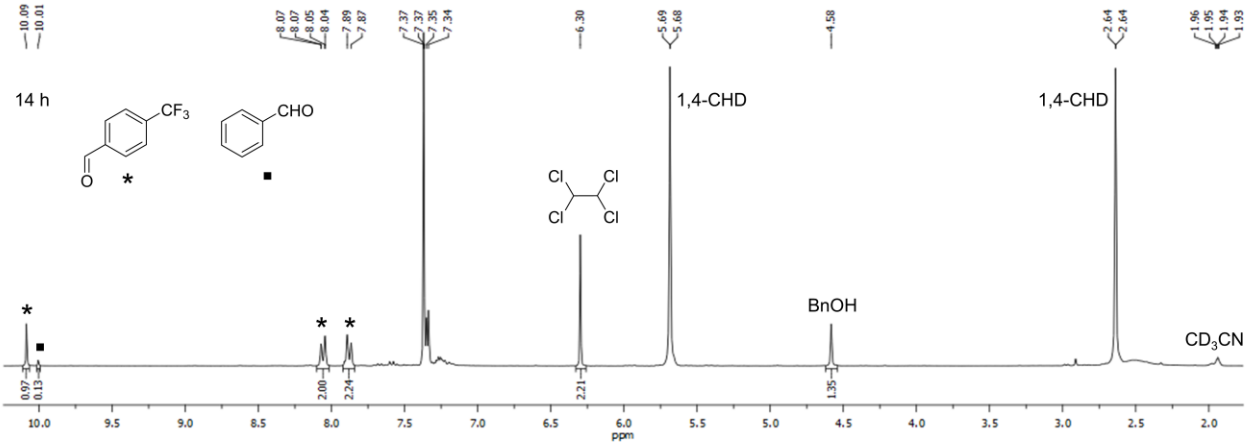
14 h
CF3
CHO
O
1,4-CHD
1,4-CHD
Cl Cl
Cl Cl
BnOH
CD3CN
ppm

1H NMR spectrum of the isolated 4-(trifluoromethyl)benzaldehyde.

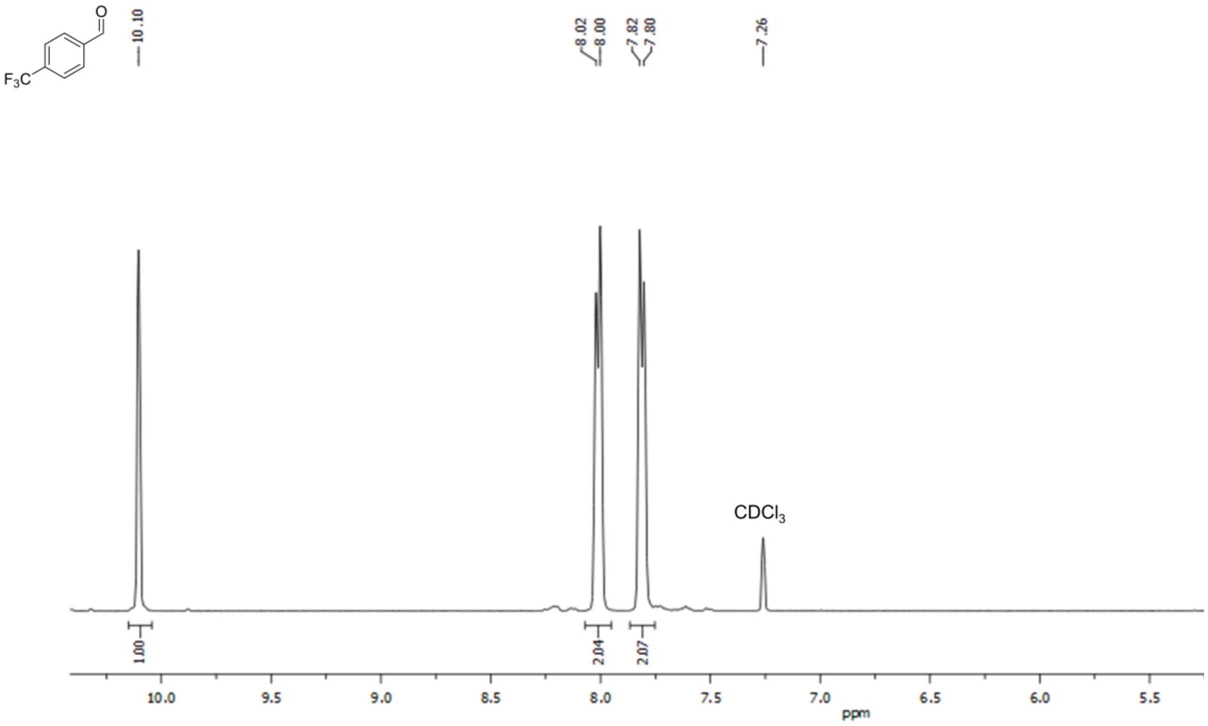

^{1}H NMR

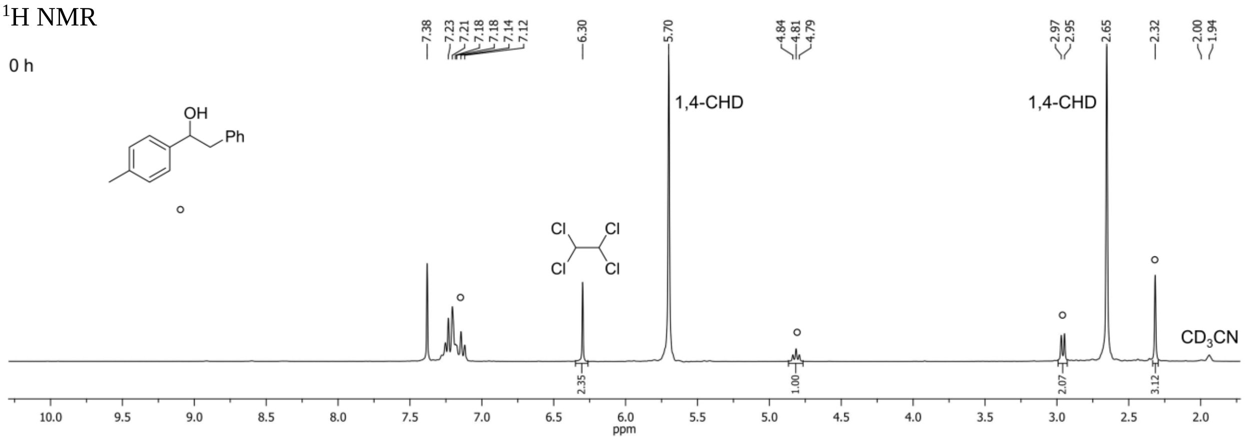

0 h
7.38
7.23
7.21
7.18
7.18
7.14
7.12
6.30
5.70
4.84
4.81
4.79
2.97
2.95
2.65
2.32
2.00
1.94
OH
Ph
1,4-CHD
1,4-CHD
Cl
Cl
Cl
Cl
CD3CN
2.35
1.00
2.07
3.12
ppm

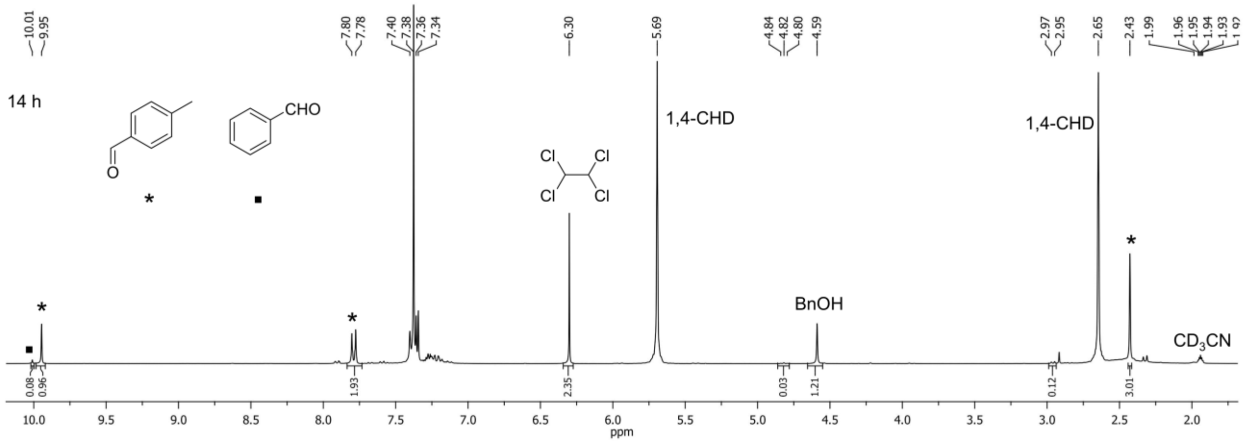

14 h
10.01
9.95
7.80
7.78
7.40
7.38
7.36
7.34
6.30
5.69
4.84
4.82
4.80
4.59
2.97
2.95
2.65
2.43
1.99
1.96
1.95
1.94
1.93
CHO
O
1,4-CHD
1,4-CHD
Cl
Cl
Cl
Cl
BnOH
CD3CN
0.08
0.96
1.93
2.35
0.03
1.21
0.12
3.01
ppm

^{1}H NMR

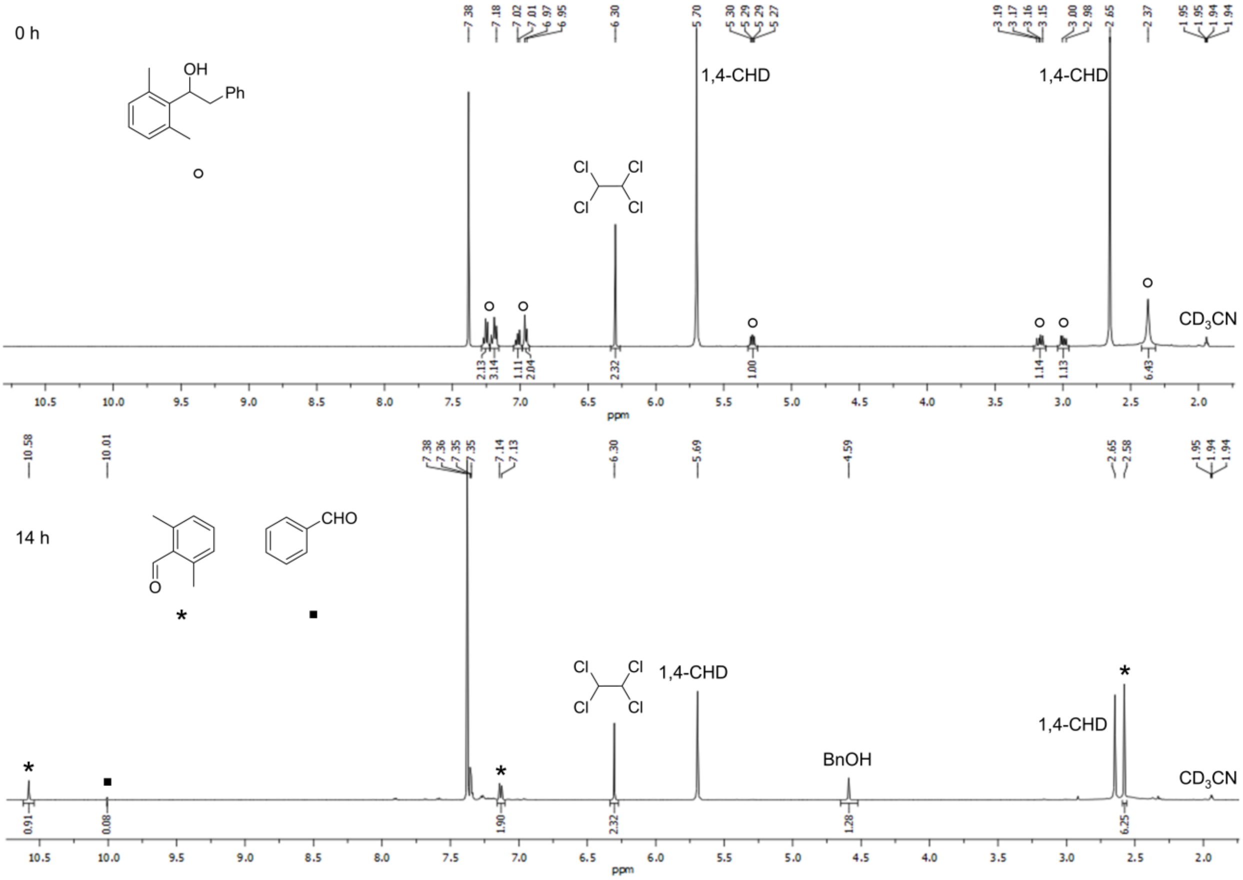

^{1}H NMR

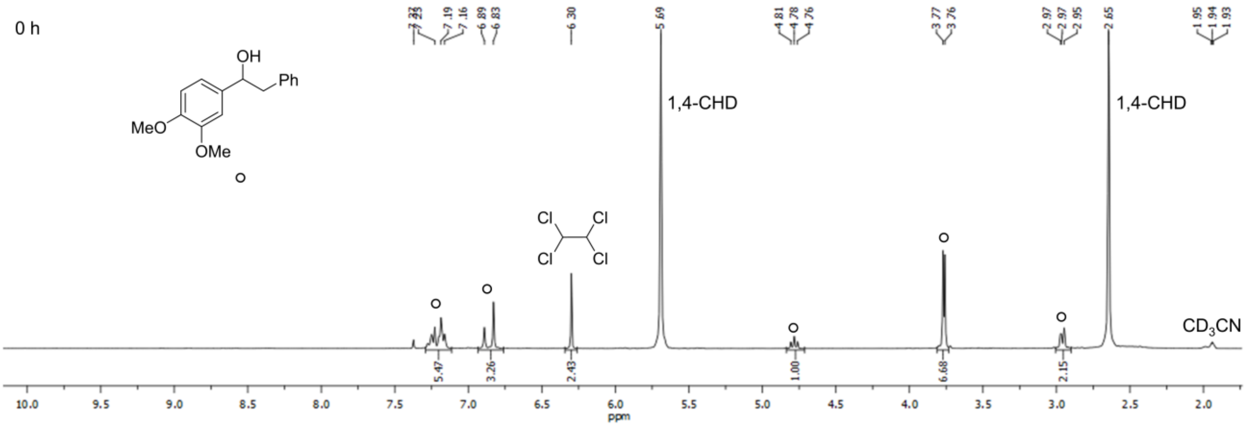
0 h
OH
Ph
MeO
OMe
1,4-CHD
1,4-CHD
Cl
Cl
Cl
Cl
CD3CN
ppm

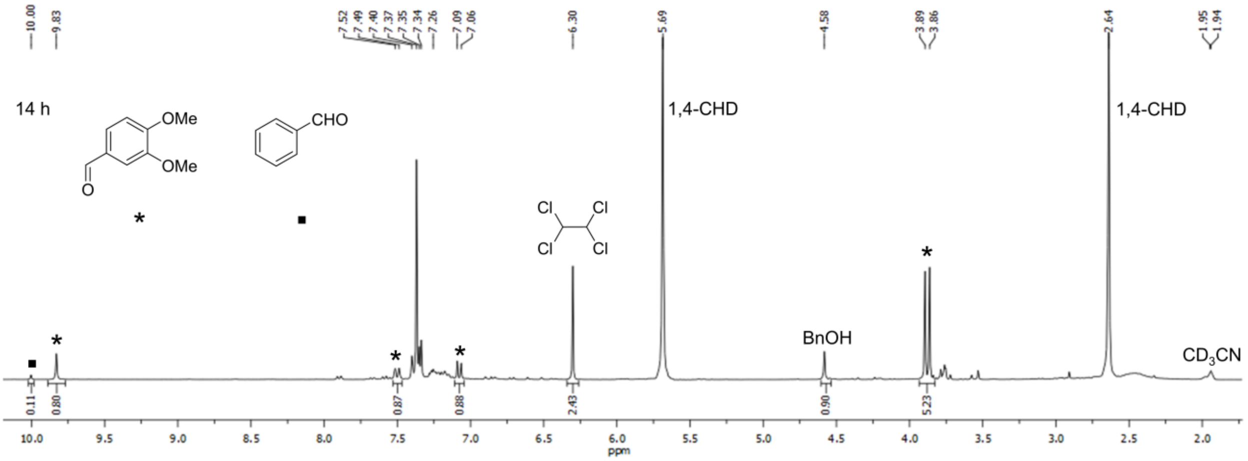
14 h
OMe
OMe
O
CHO
1,4-CHD
1,4-CHD
Cl
Cl
Cl
Cl
BnOH
CD3CN
ppm

^{1}H NMR spectrum of the isolated 3,4-dimethoxybenzaldehyde.

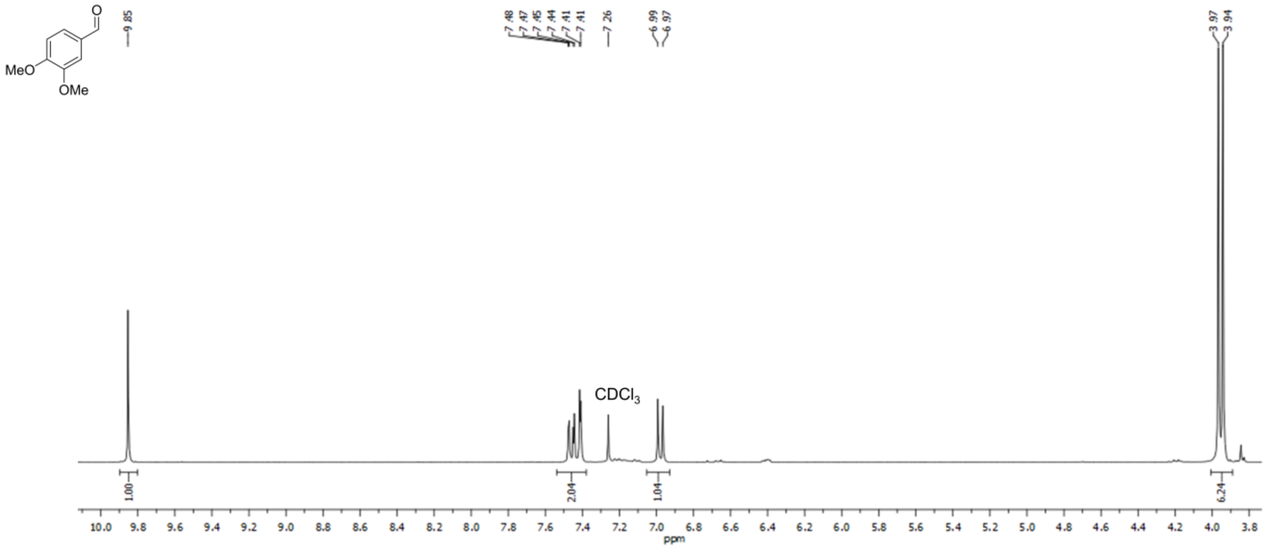

^{1}H NMR

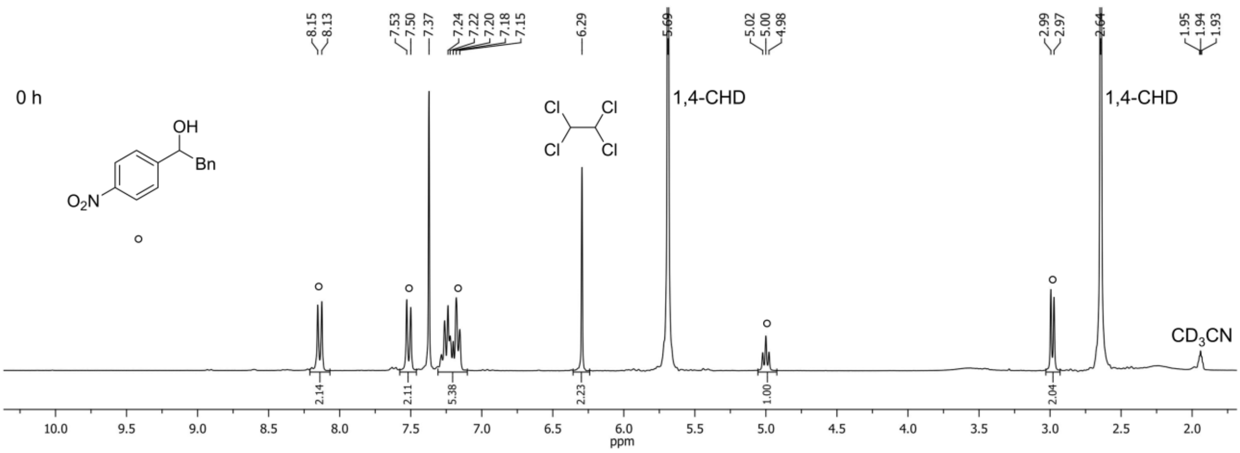
0 h
OH
Bn
O2N
Cl
Cl
Cl
Cl
1,4-CHD
1,4-CHD
CD3CN
ppm

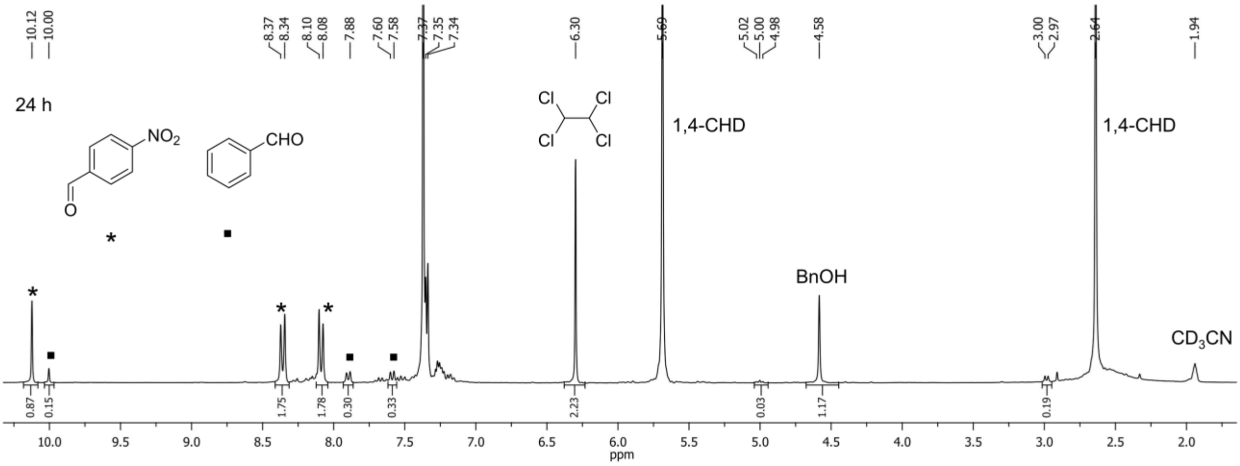
24 h
NO2
O
CHO
Cl
Cl
Cl
Cl
1,4-CHD
1,4-CHD
BnOH
CD3CN
ppm

^{1}H NMR

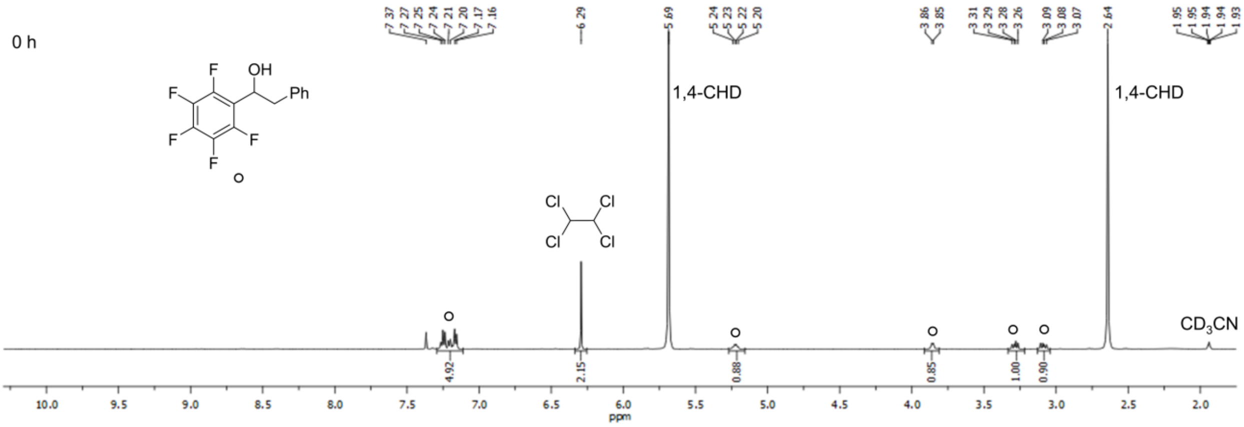
0 h
F
OH
Ph
F
F
F
F
1,4-CHD
1,4-CHD
Cl
Cl
Cl
Cl
CD3CN
4.92
2.15
0.88
0.85
1.00
0.90
ppm

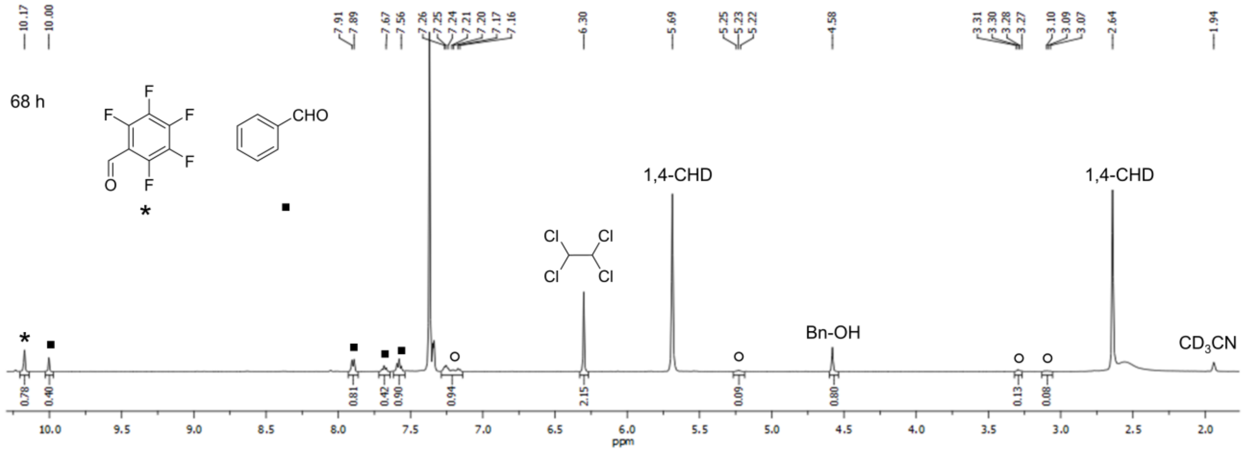
68 h
F
F
F
F
O
F
CHO
*
1,4-CHD
1,4-CHD
Cl
Cl
Cl
Cl
Bn-OH
CD3CN
0.78
0.40
0.81
0.42
0.90
0.94
2.15
0.09
0.80
0.13
0.08
ppm

^{1}H NMR spectrum of the isolated 2,3,4,5,6-pentafluorobenzaldehyde.

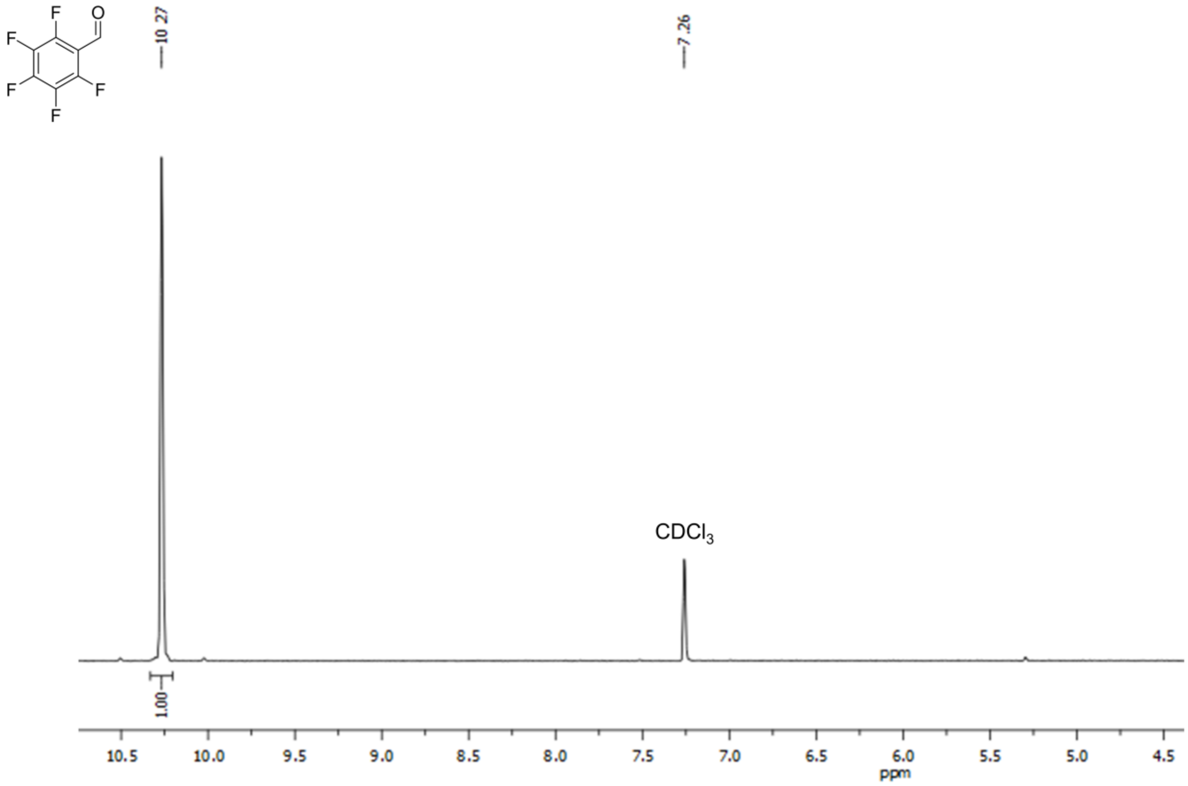

^{1}H NMR

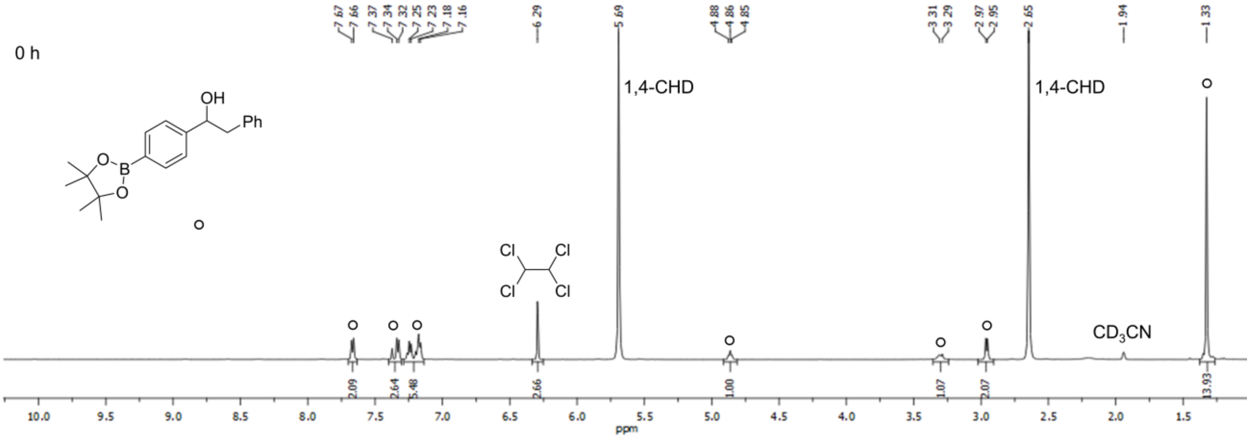
0 h
OH
Ph
O
B
O
1,4-CHD
1,4-CHD
Cl
Cl
Cl
Cl
CD3CN
ppm

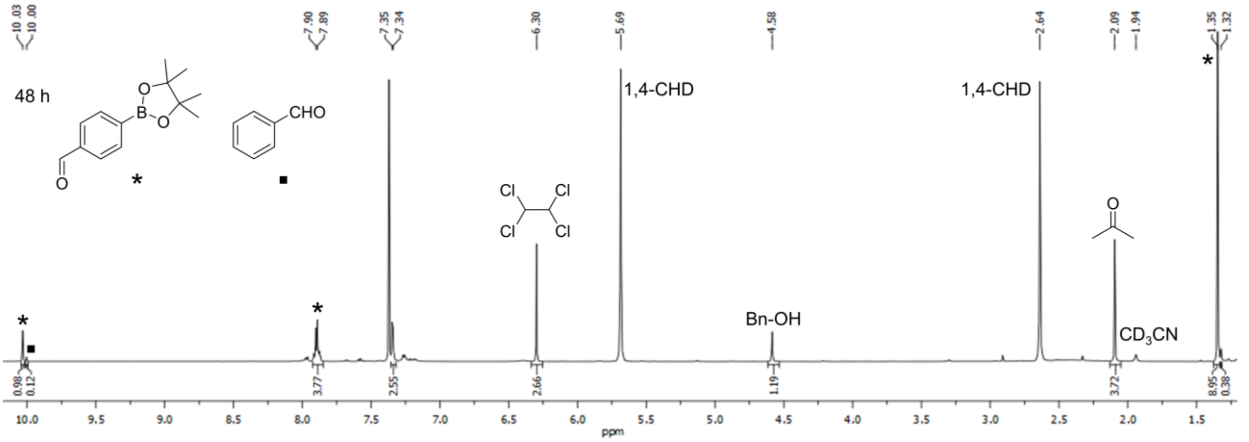
48 h
O
B
O
O
CHO
1,4-CHD
1,4-CHD
Cl
Cl
Cl
Cl
Bn-OH
O
CD3CN
ppm

^{1}H NMR

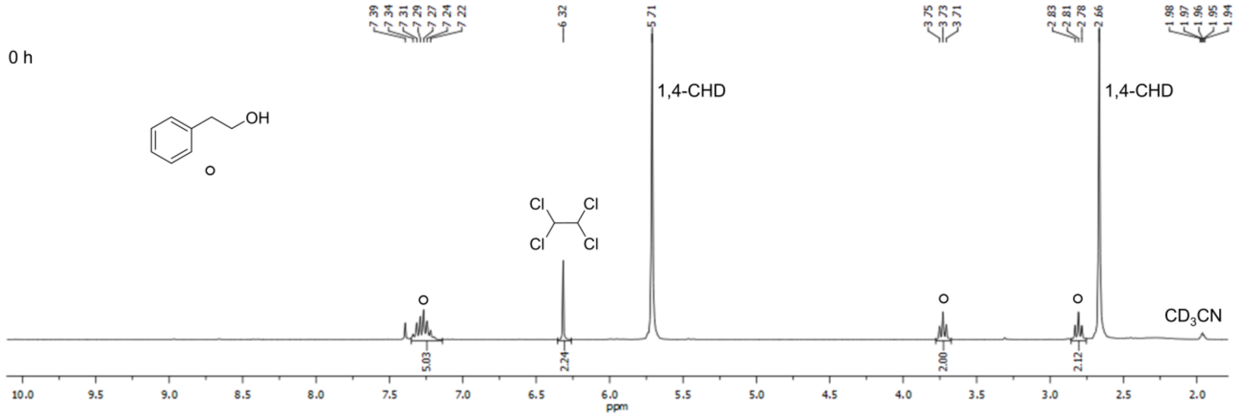
0 h
OH
1,4-CHD
1,4-CHD
Cl Cl Cl Cl
CD3CN
ppm

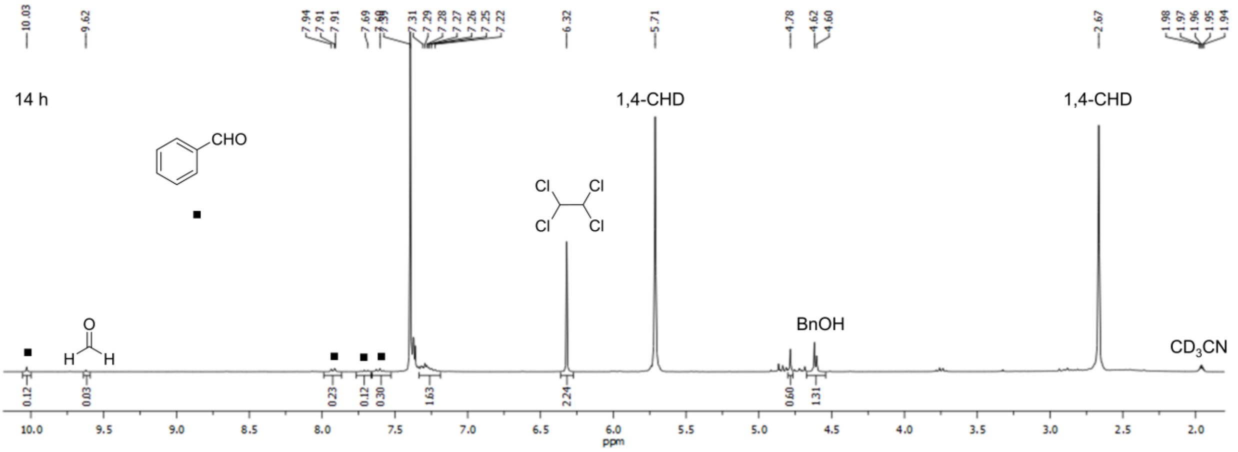
14 h
CHO
1,4-CHD
1,4-CHD
Cl Cl Cl Cl
BnOH
O
H H
CD3CN
ppm

^{1}H NMR

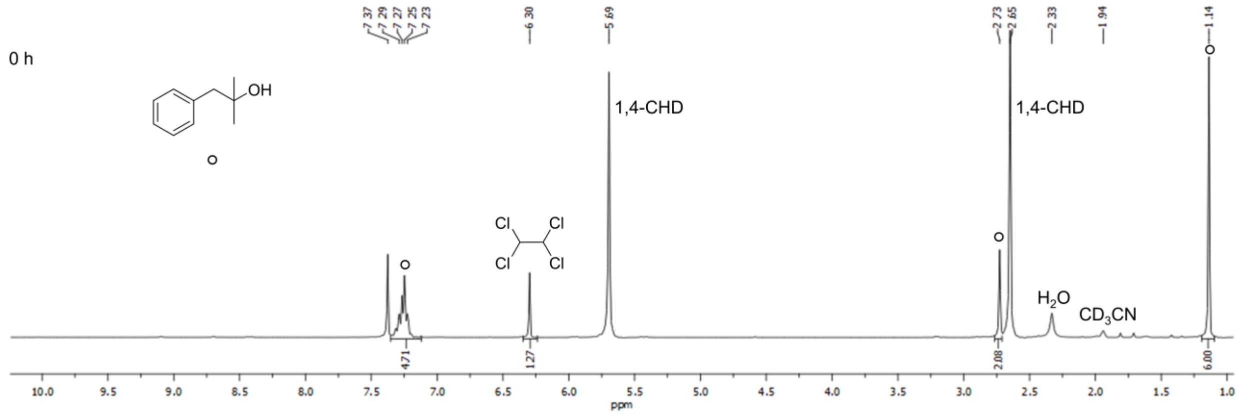
0 h
OH
1,4-CHD
1,4-CHD
Cl
Cl
Cl
Cl
H_2O
CD_3CN
ppm

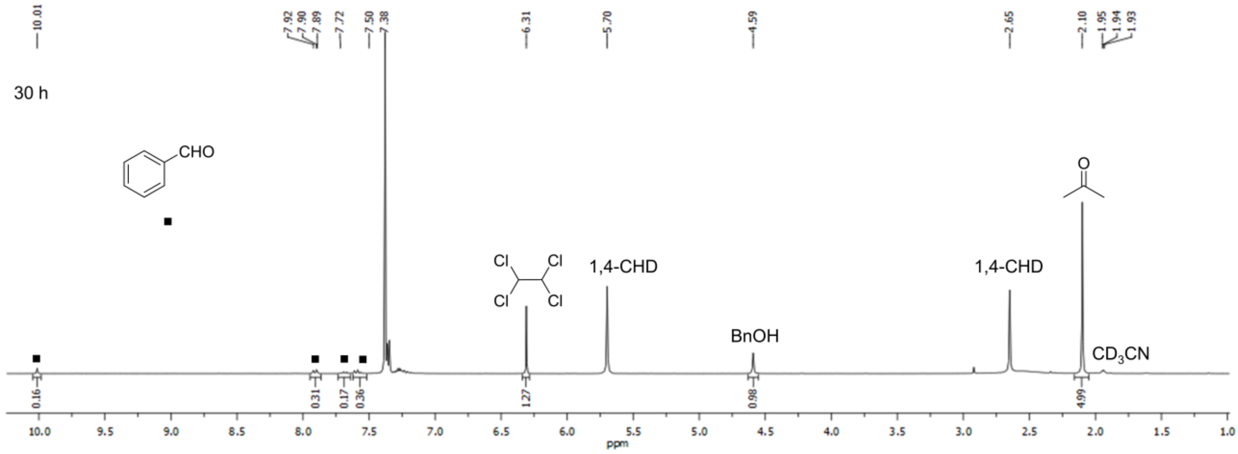
30 h
CHO
Cl
Cl
Cl
Cl
1,4-CHD
1,4-CHD
BnOH
O
CD_3CN
ppm

^{1}H NMR

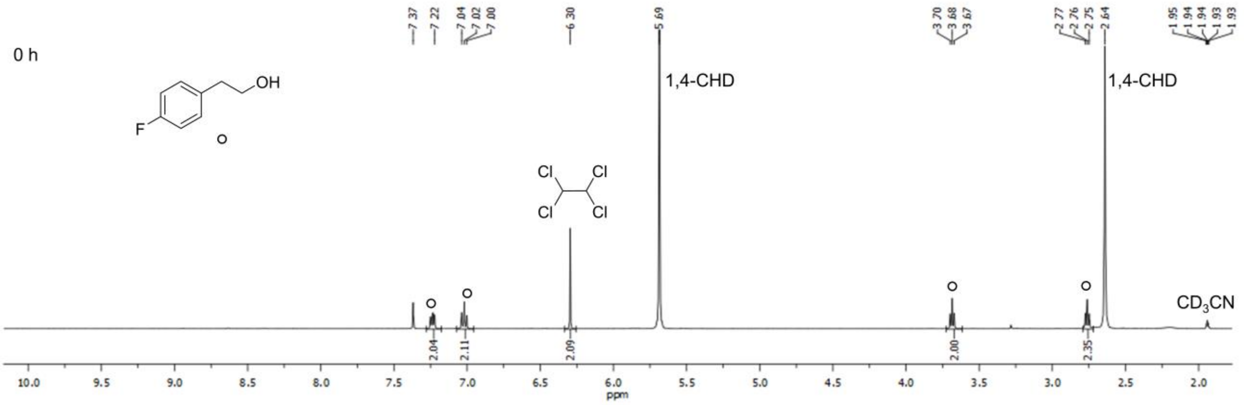
0 h
OH
F
1,4-CHD
1,4-CHD
Cl
Cl
Cl
Cl
CD3CN
ppm

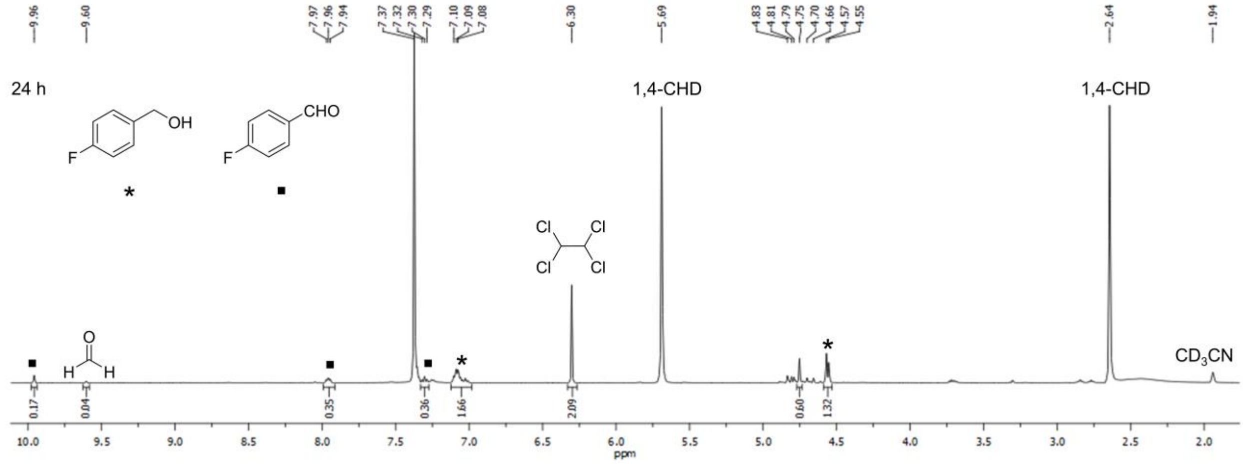
24 h
OH
F
CHO
F
1,4-CHD
1,4-CHD
Cl
Cl
Cl
Cl
O
H
H
CD3CN
ppm

^{1}H NMR

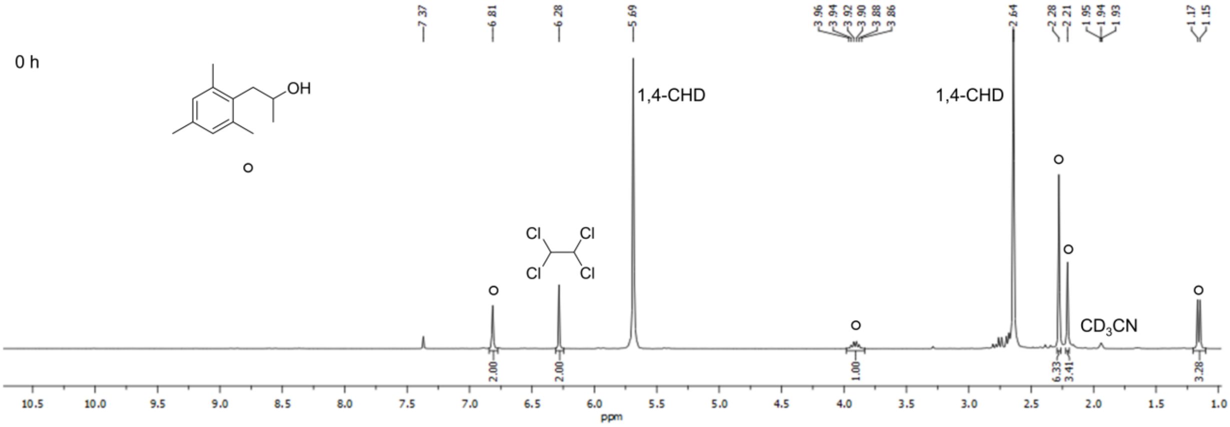
0 h
OH
1,4-CHD
1,4-CHD
Cl
Cl
Cl
Cl
CD3CN
ppm

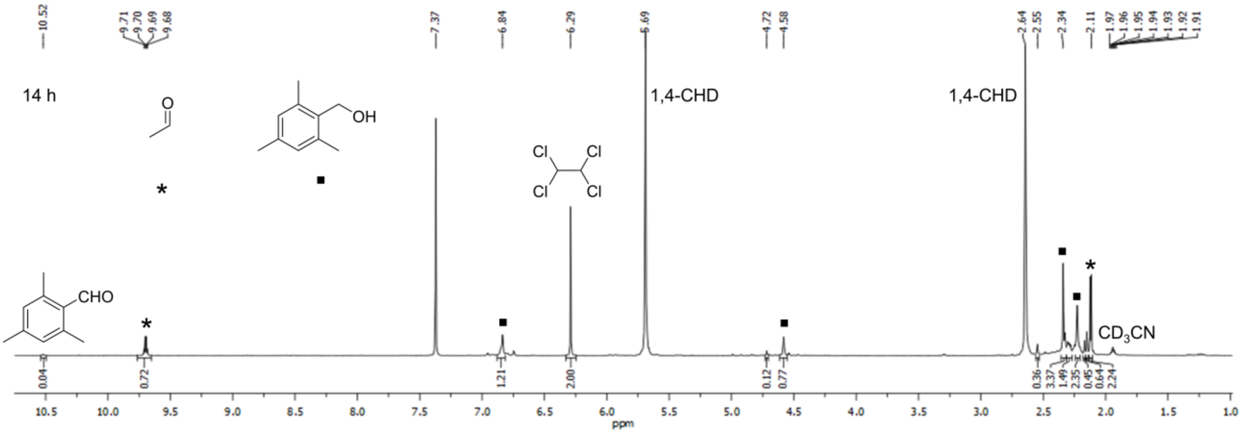
14 h
O
OH
CHO
1,4-CHD
1,4-CHD
Cl
Cl
Cl
Cl
CD3CN
ppm

^{1}H NMR

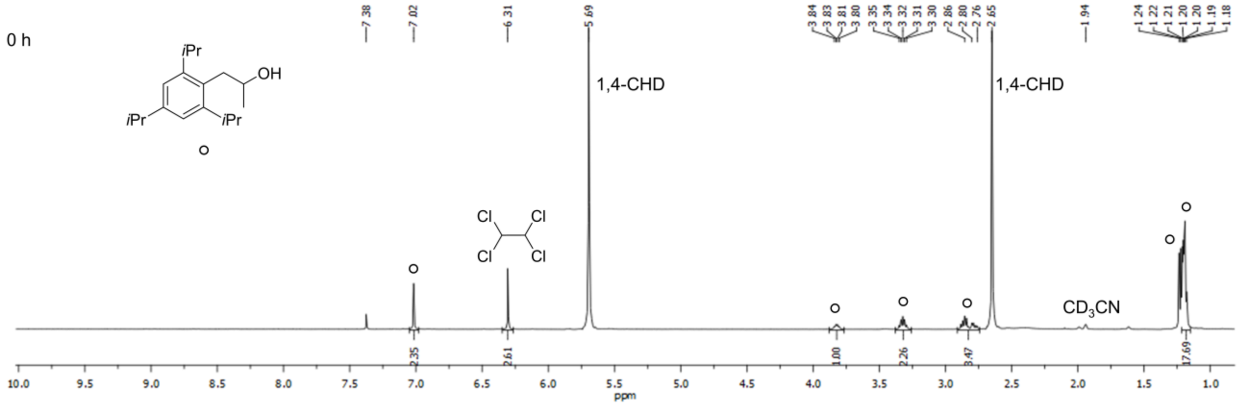
0 h
iPr
OH
iPr
iPr
1,4-CHD
1,4-CHD
Cl
Cl
Cl
Cl
CD3CN
ppm

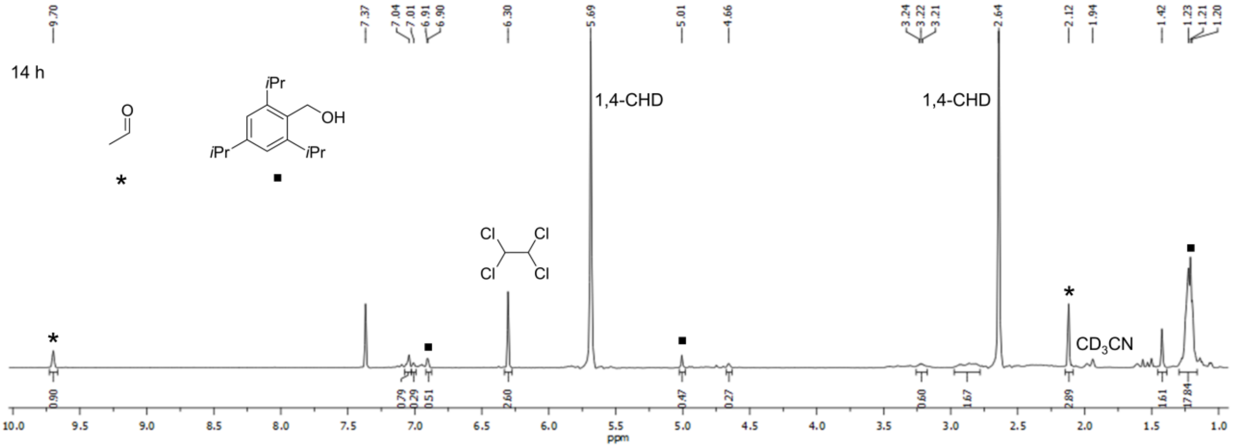
14 h
O
iPr
OH
iPr
iPr
1,4-CHD
1,4-CHD
Cl
Cl
Cl
Cl
CD3CN
ppm

^{1}H NMR

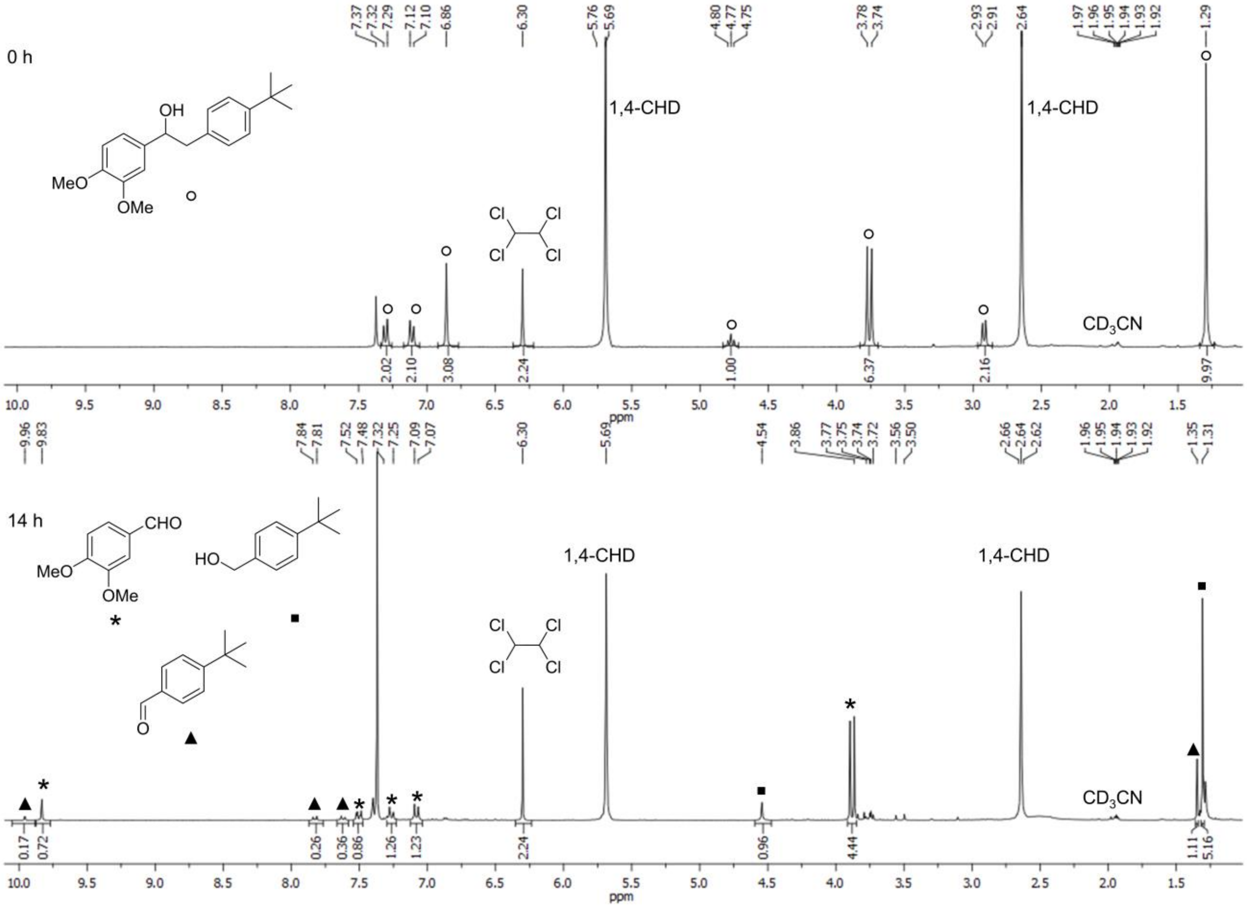

^{1}H NMR spectrum of the isolated 4-(*tert*-butyl)benzaldehyde.

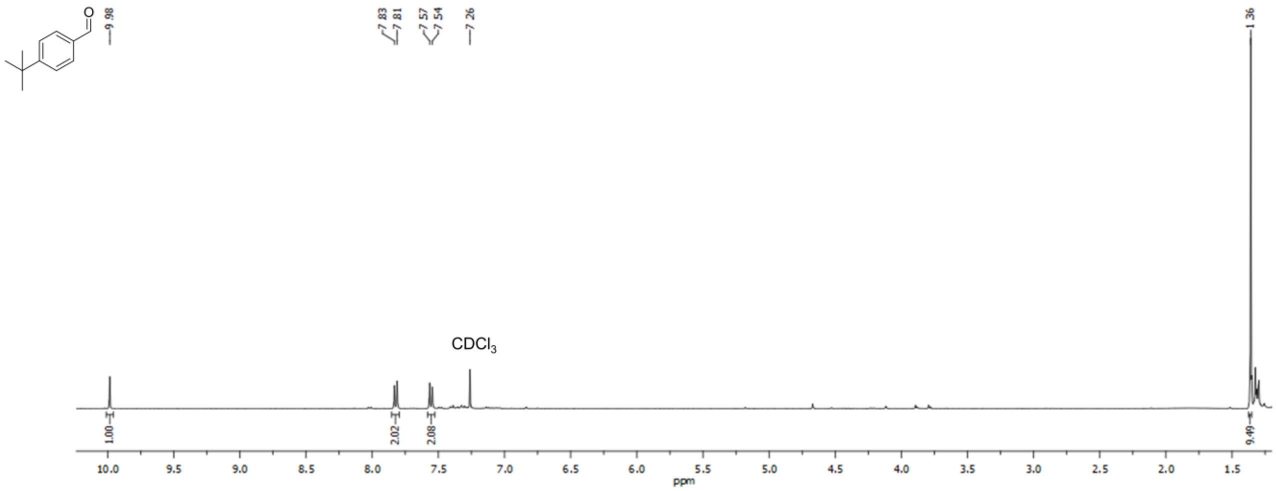

^{1}H NMR spectrum of the isolated (4-(*tert*-butyl)phenyl)methanol.

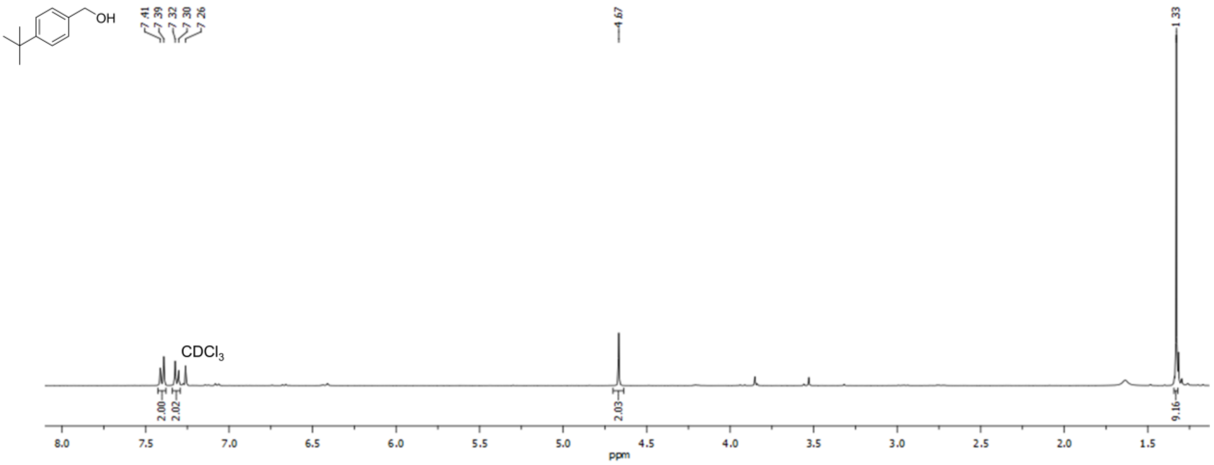

^{1}H NMR spectra before and after irradiation of **23**. This reaction was performed on a 0.020 mmol scale and with a pure O_2 balloon to minimize side-reactions.

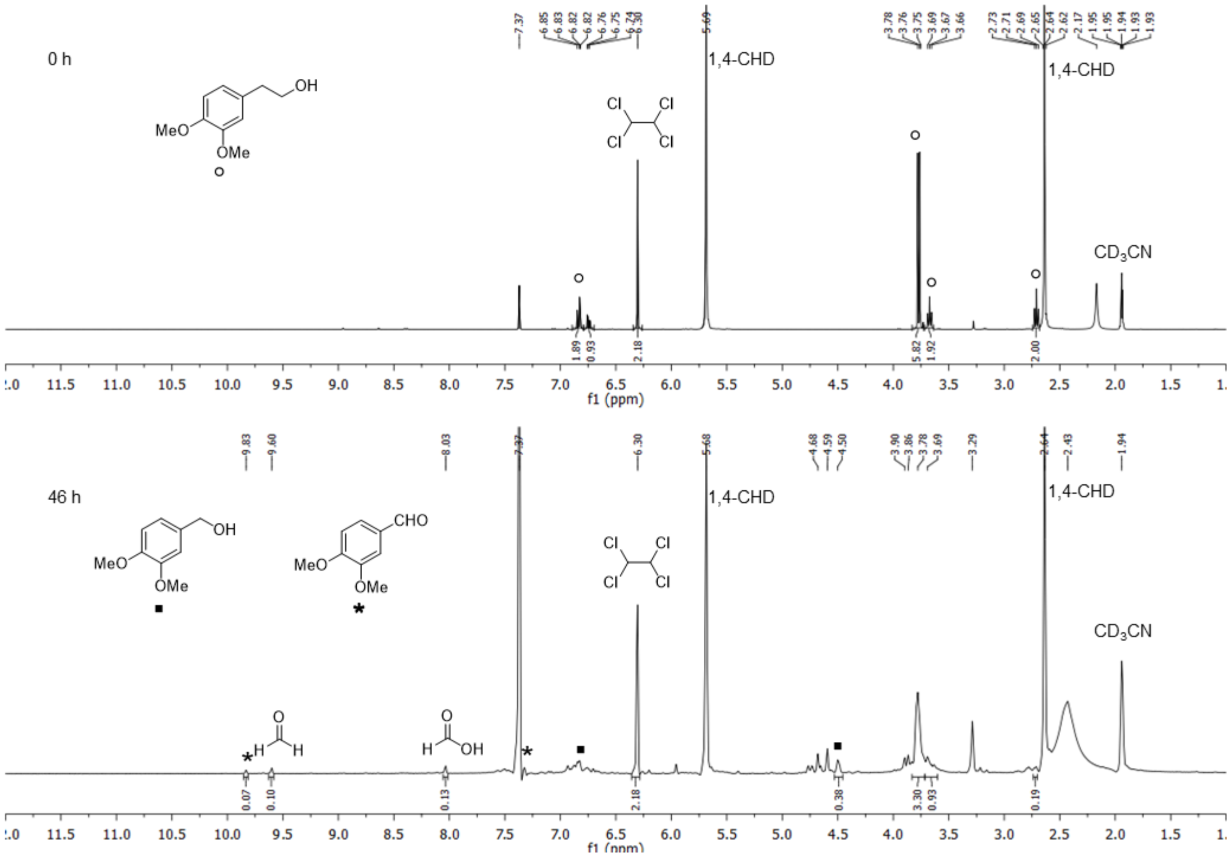

^{1}H NMR

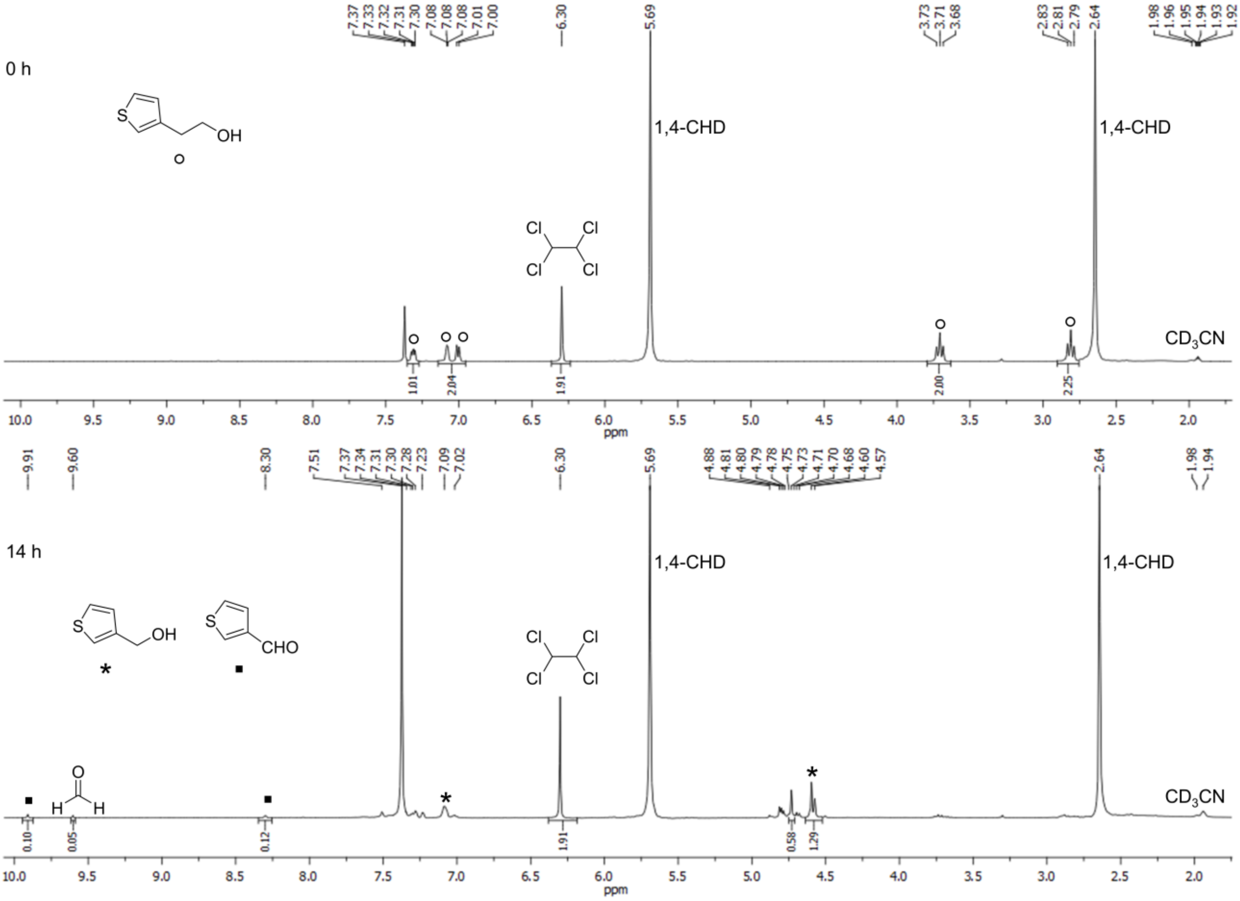

^{1}H NMR

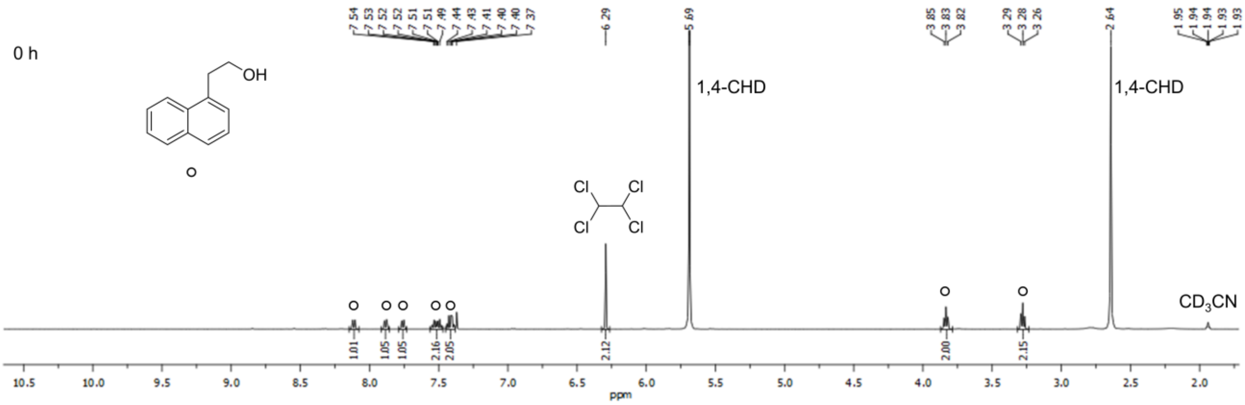
0 h
OH
1,4-CHD
1,4-CHD
Cl
Cl
Cl
Cl
CD3CN
ppm

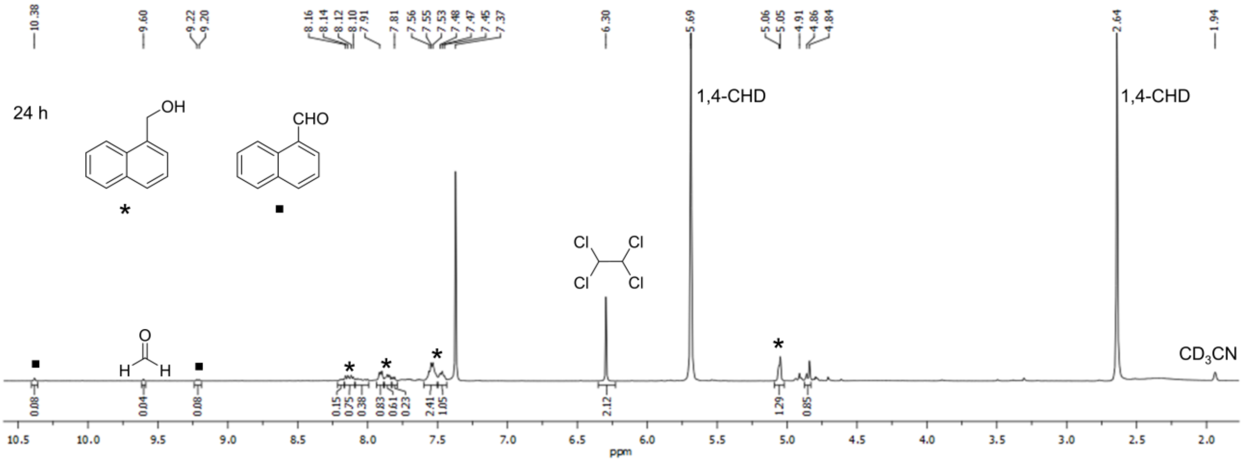
24 h
OH
CHO
1,4-CHD
1,4-CHD
Cl
Cl
Cl
Cl
O
H
H
CD3CN
ppm

^{1}H NMR

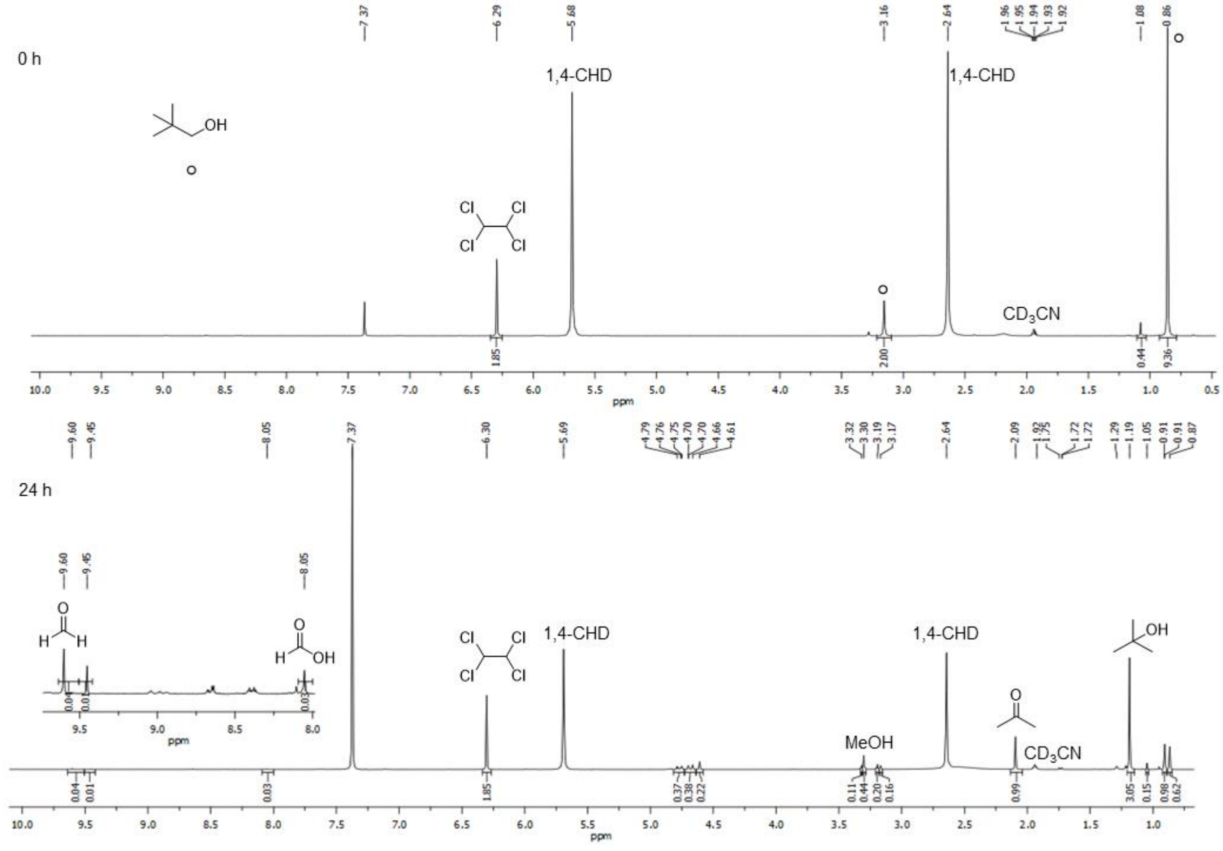

^{1}H NMR

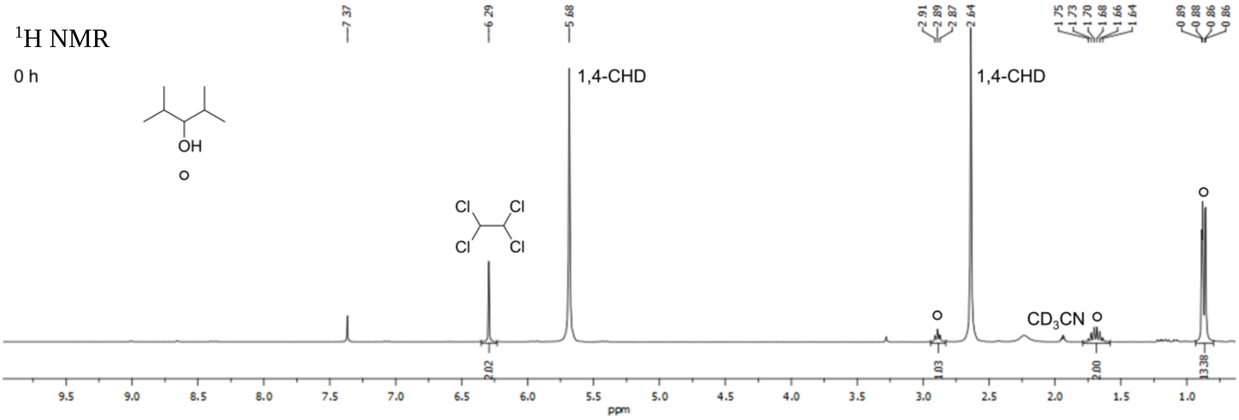
0 h
OH
Cl Cl Cl Cl
1,4-CHD
1,4-CHD
CD3CN
7.37
6.29
5.68
2.91 2.89 2.87 2.64
1.75 1.73 1.70 1.68 1.66 1.64
0.89 0.88 0.86 0.86
2.02
1.03
2.00
13.38
ppm

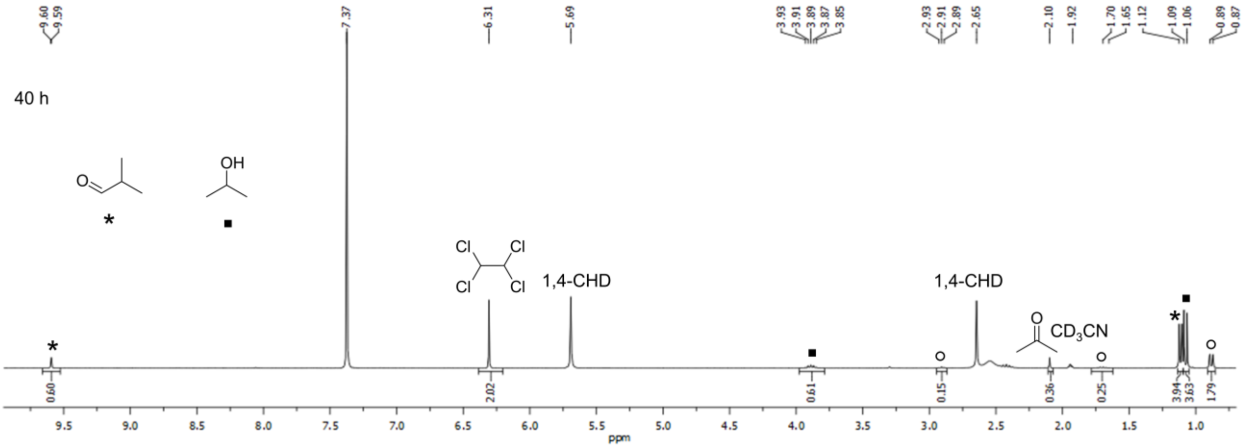
40 h
O
OH
*
Cl Cl Cl Cl
1,4-CHD
1,4-CHD
CD3CN
9.60 9.59
7.37
6.31
5.69
3.93 3.91 3.89 3.87 3.85
2.93 2.91 2.89 2.65
2.10
1.92
1.70 1.65 1.12 1.09 1.06
0.89 0.87
0.60
2.02
0.61
0.15
0.36
0.25
3.94 3.63
1.79
ppm

^{1}H NMR

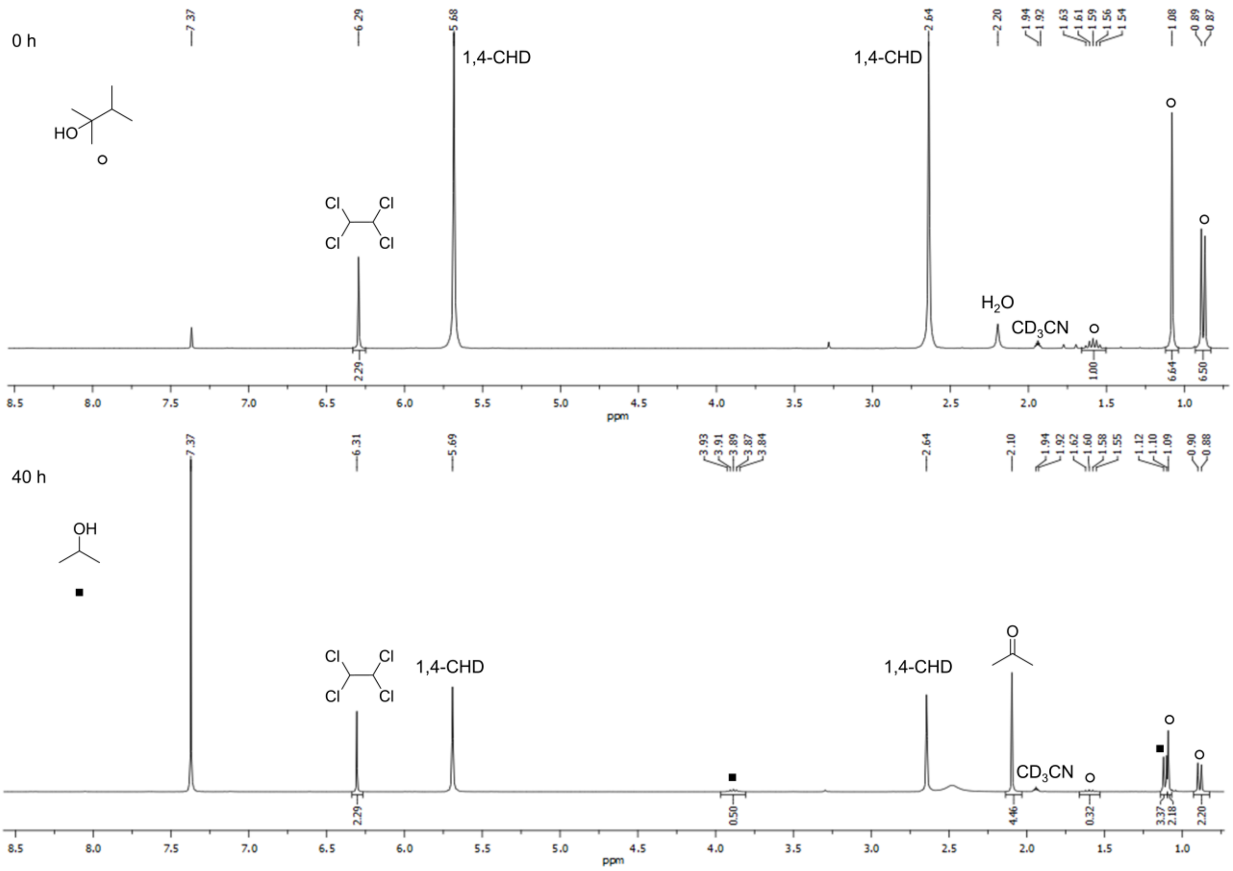
0 h
1,4-CHD
1,4-CHD
HO
Cl
Cl
Cl
Cl
H2O
CD3CN
2.29
1.00
6.64
6.50
ppm
40 h
OH
Cl
Cl
Cl
Cl
1,4-CHD
1,4-CHD
O
CD3CN
2.29
0.50
4.46
0.32
3.37
2.18
2.20
ppm

^{1}H NMR

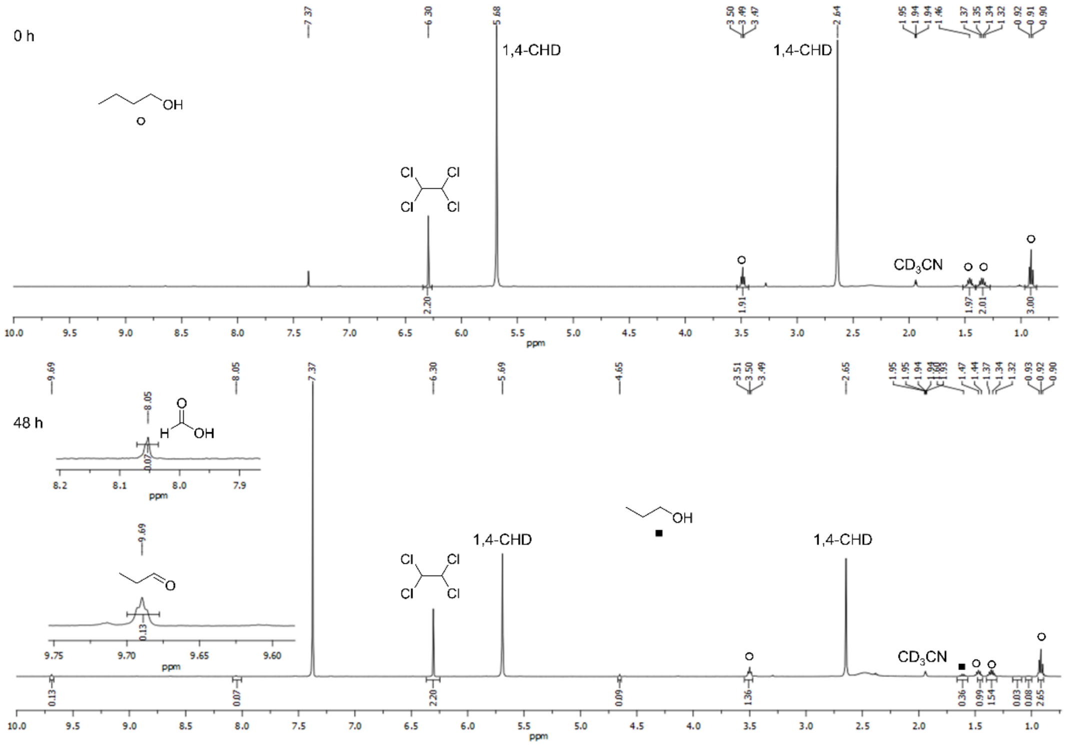

^{1}H NMR

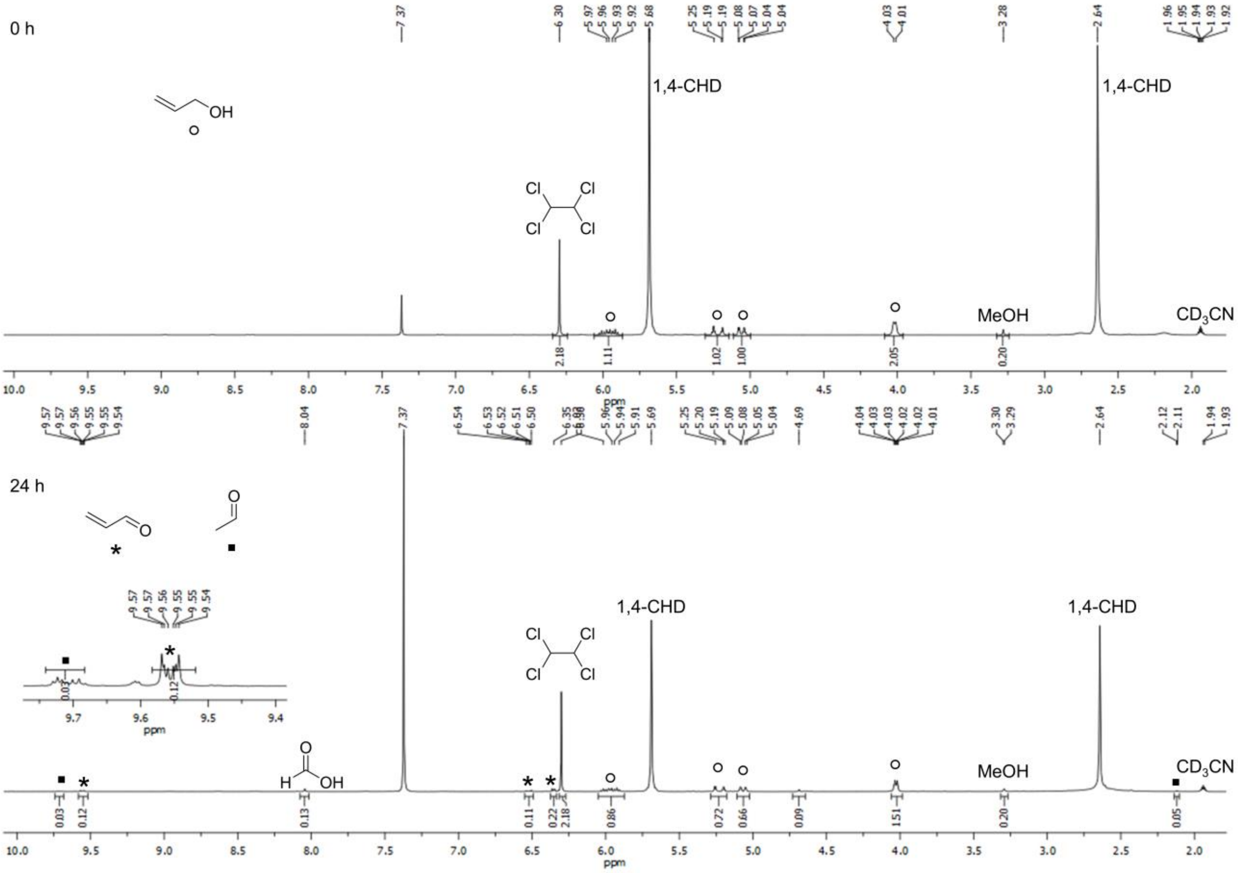
0 h
1,4-CHD
1,4-CHD
OH
MeOH
CD3CN
24 h
1,4-CHD
1,4-CHD
H OH
MeOH
CD3CN
ppm
ppm

^{1}H NMR

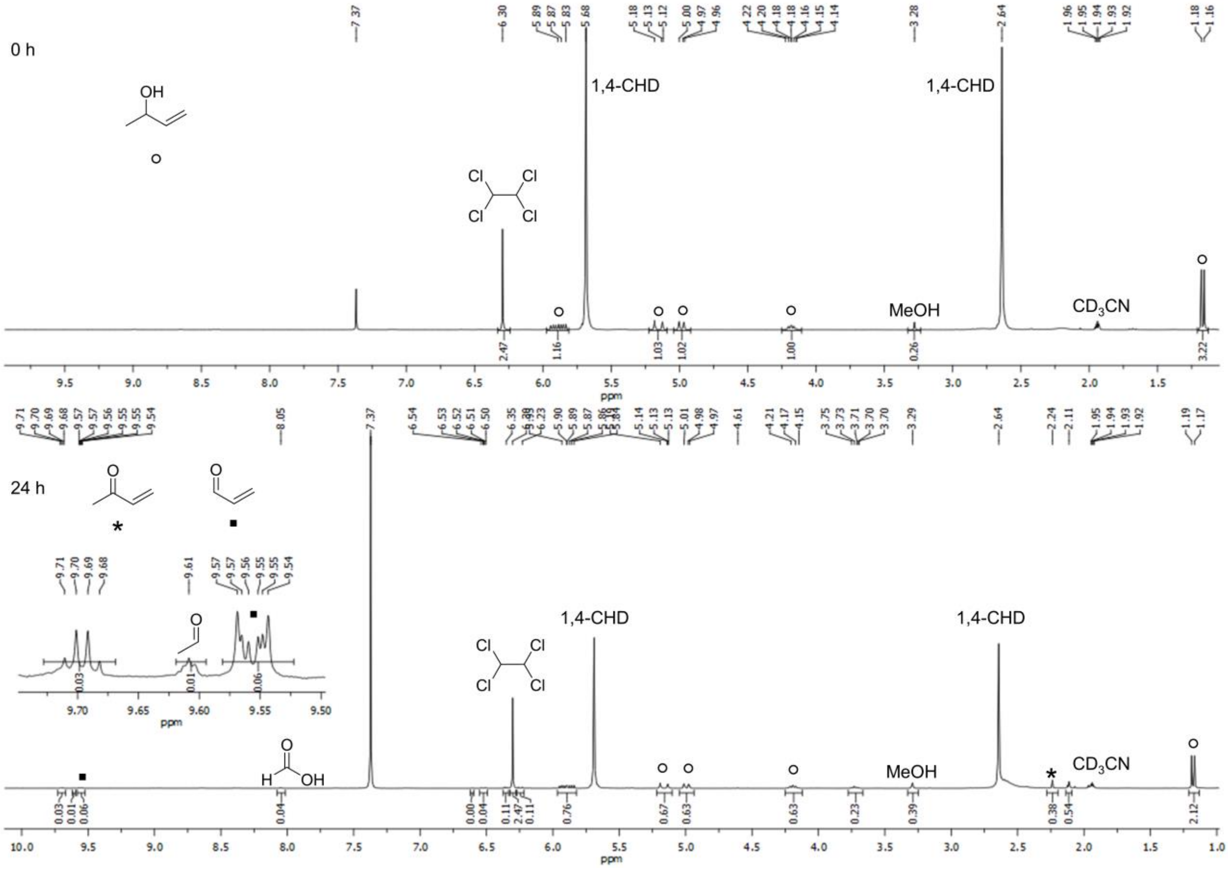

^{1}H NMR

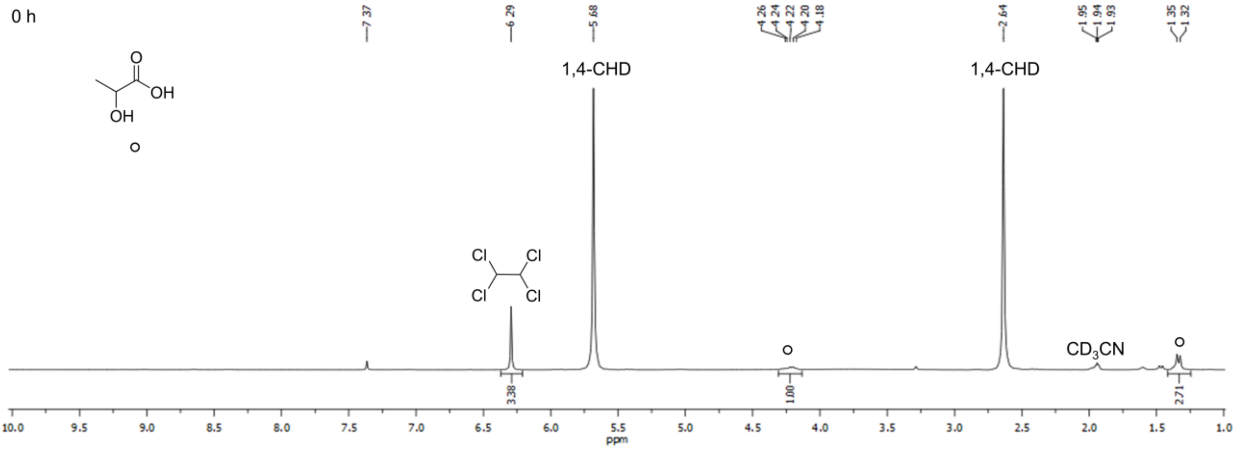
0 h
7.37
6.29
5.68
4.26 4.24 4.22 4.20 4.18
2.64
1.95 1.94 1.93
1.35 1.32
1,4-CHD
1,4-CHD
OH
OH
O
Cl Cl
Cl Cl
CD3CN
3.38
1.00
2.71
10.0 9.5 9.0 8.5 8.0 7.5 7.0 6.5 6.0 5.5 5.0 4.5 4.0 3.5 3.0 2.5 2.0 1.5 1.0
ppm

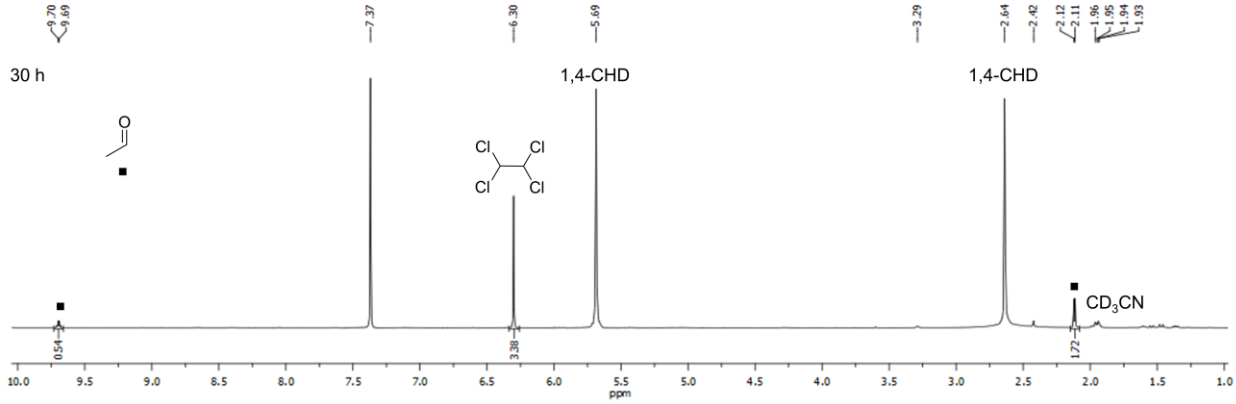
9.70 9.69
7.37
6.30
5.69
3.29
2.64
2.42
2.12 2.11
1.96 1.95 1.94 1.93
30 h
1,4-CHD
1,4-CHD
O
Cl Cl
Cl Cl
CD3CN
0.54
3.38
1.72
10.0 9.5 9.0 8.5 8.0 7.5 7.0 6.5 6.0 5.5 5.0 4.5 4.0 3.5 3.0 2.5 2.0 1.5 1.0
ppm

^{1}H NMR spectra of reaction mixture of **34** with 2 eq. of MeOH with respect to the monomer unit.

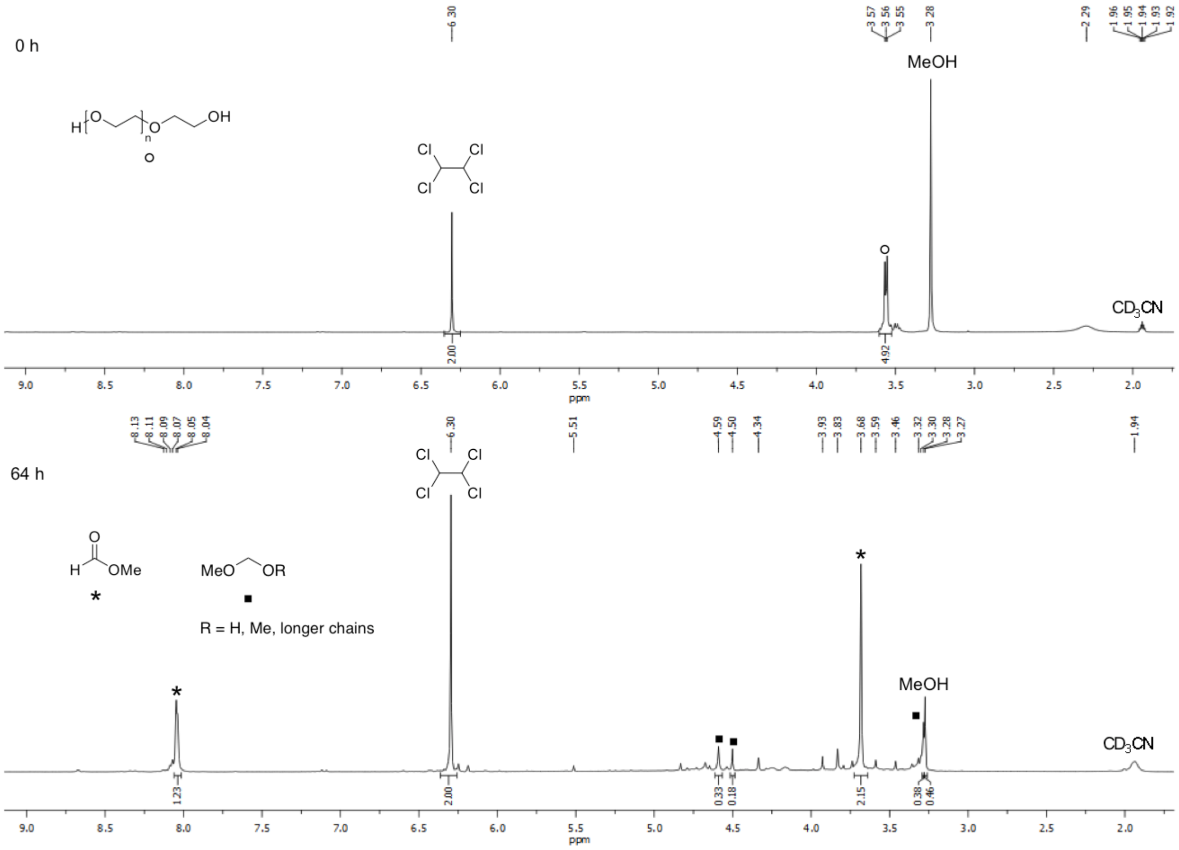

^{1}H NMR after vacuum transfer of volatiles from the reaction mixture of **34** with 2 eq. of MeOH with respect to the monomer unit.

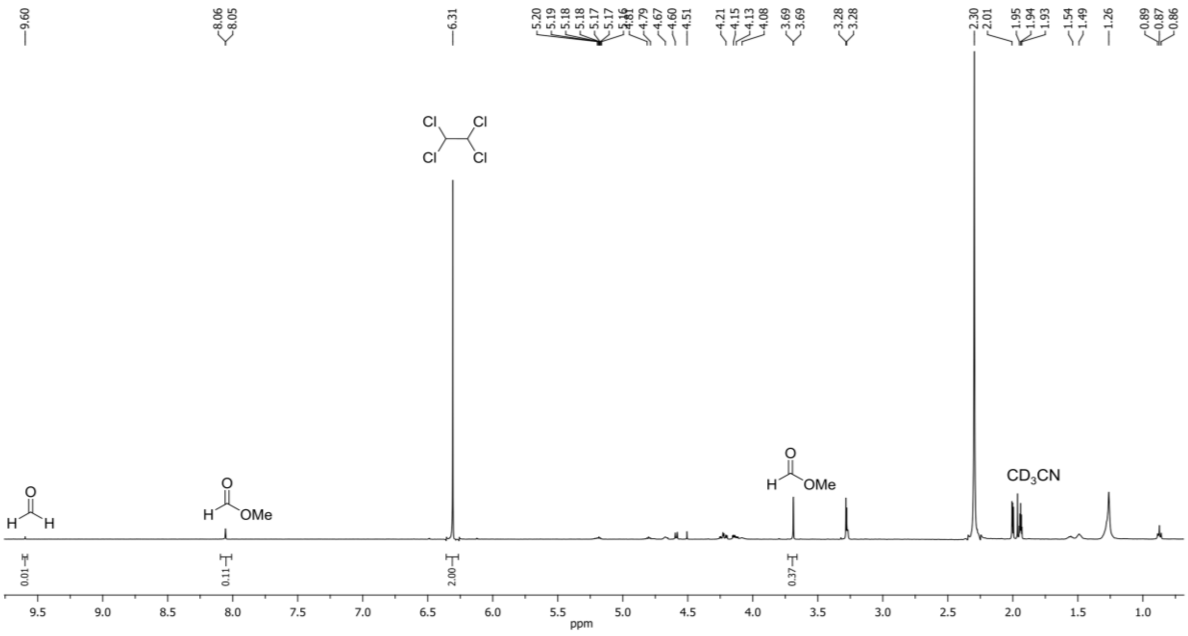

^{1}H NMR spectra of reaction mixture of **34** with 2 eq. of CD_3OD with respect to the monomer unit.

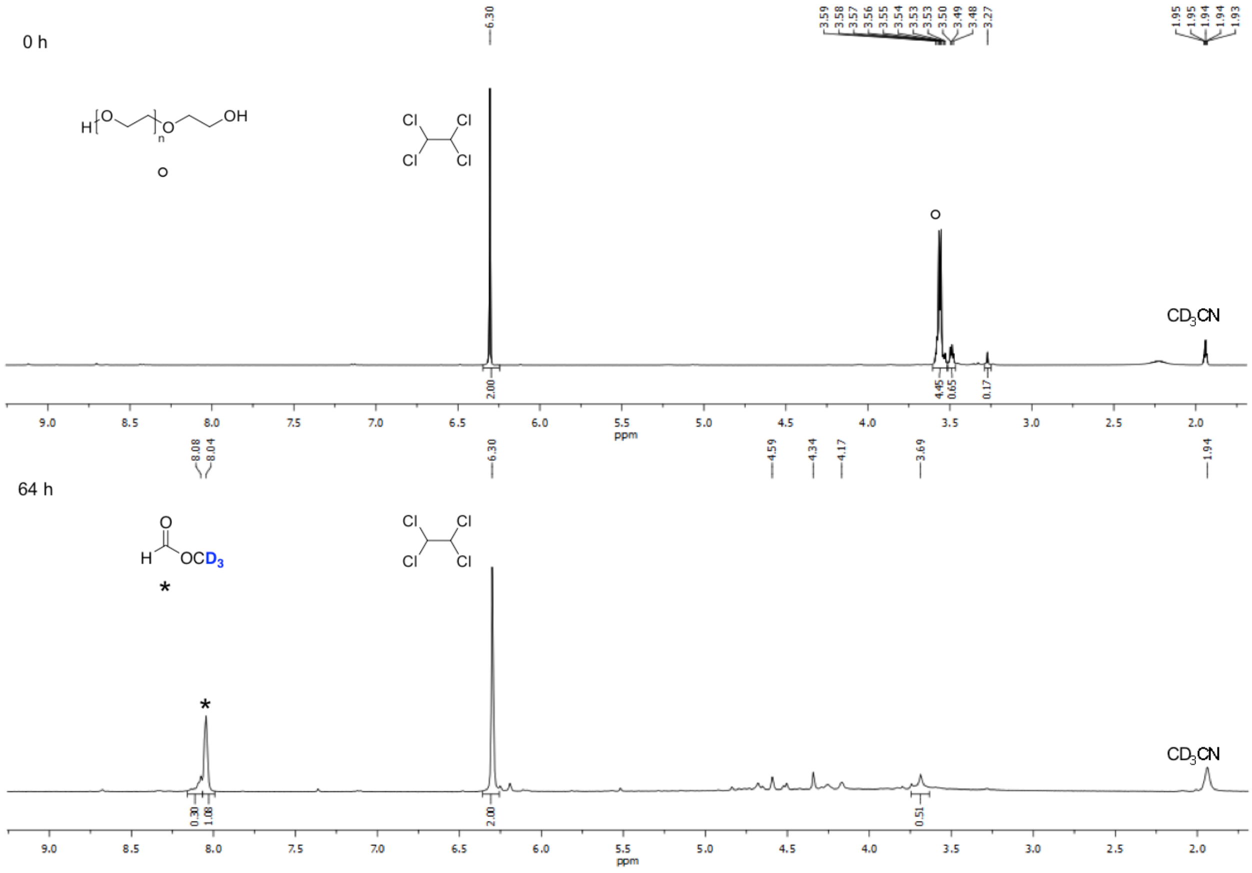

^{1}H NMR spectra of reaction mixture of **35** (without MeOH)

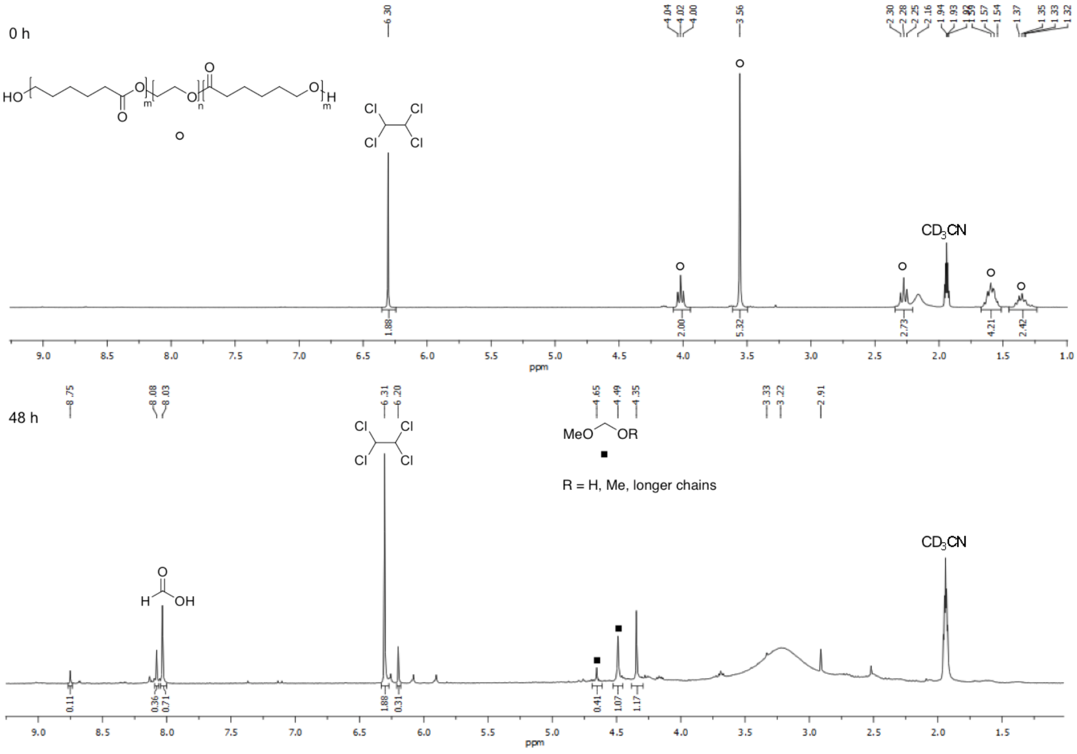

^{1}H NMR spectrum after vacuum transfer of volatiles from the crude reaction mixture of **35** with 2 eq. of added MeOH with respect to the monomer unit.

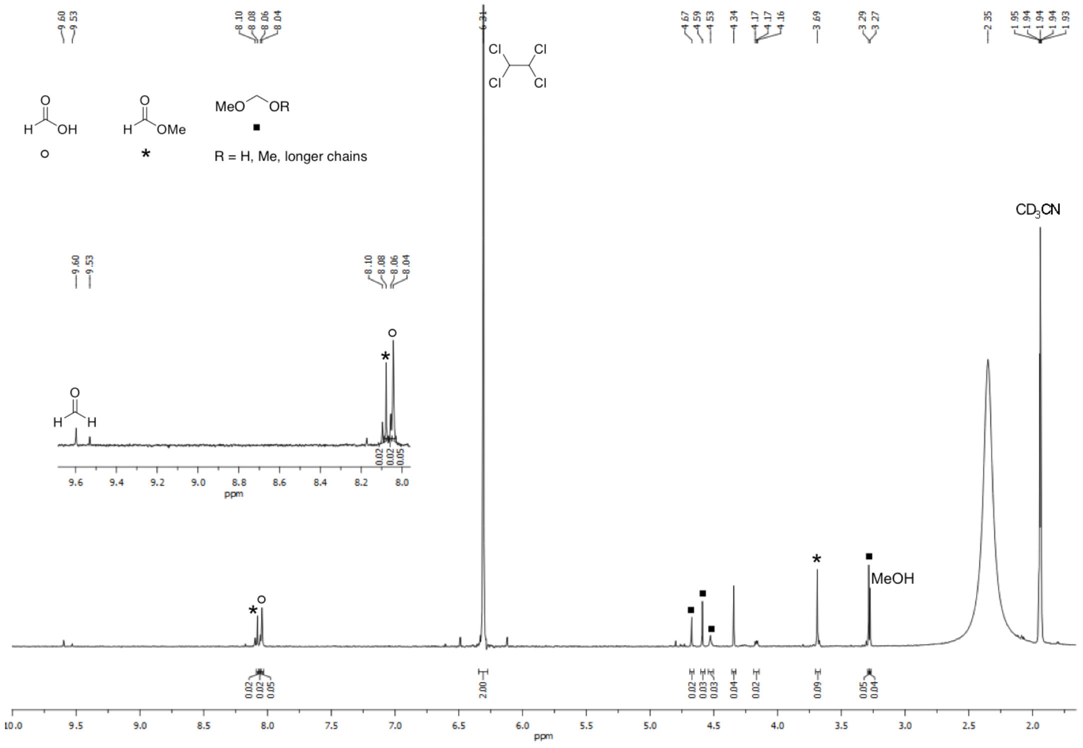

^{1}H NMR

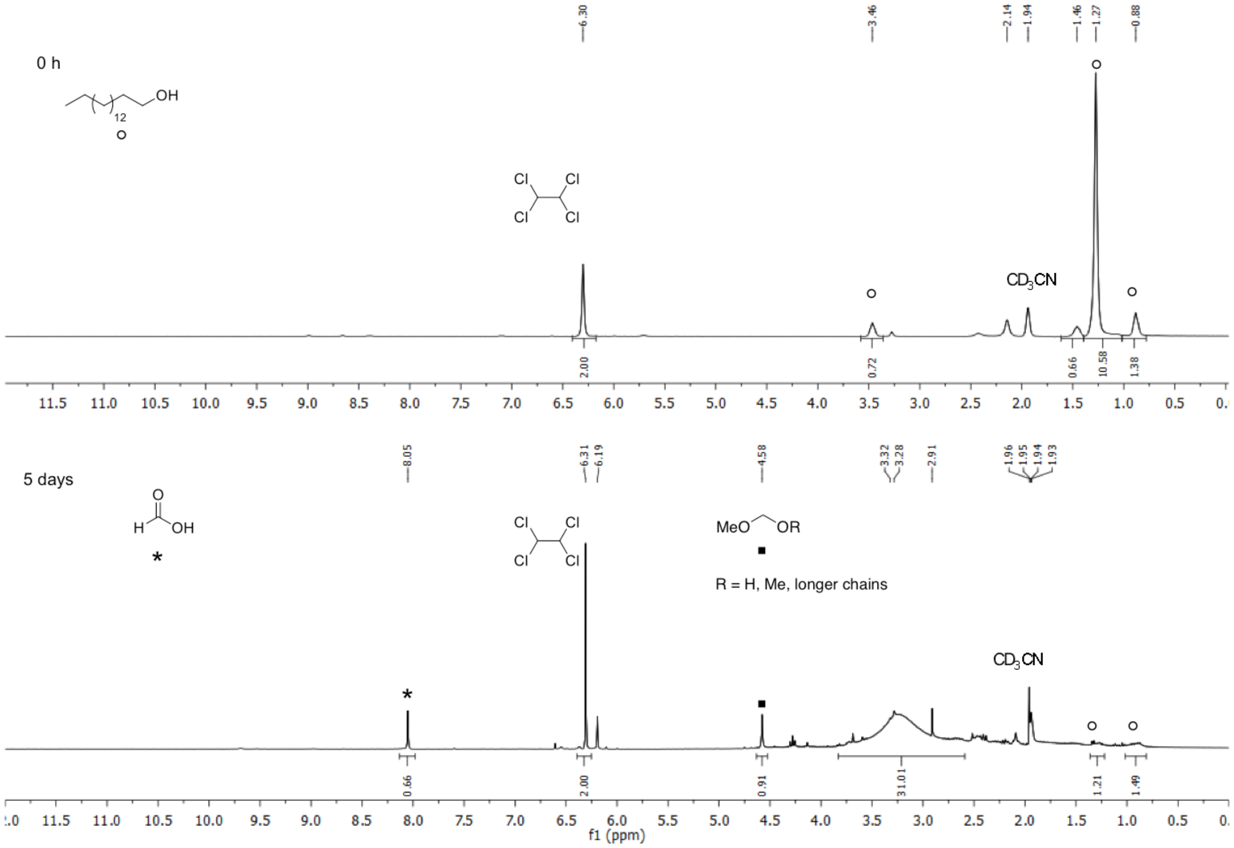
0 h
OH
12
Cl
Cl
Cl
Cl
CD3CN
6.30
3.46
2.14
1.94
1.46
1.27
0.88
2.00
0.72
0.66
10.58
1.38
5 days
H
O
OH
*
MeO
OR
R = H, Me, longer chains
CD3CN
8.05
6.31
6.19
4.58
3.32
3.28
2.91
1.96
1.95
1.94
1.93
0.66
2.00
0.91
31.01
1.21
1.49
f1 (ppm)

^1H NMR

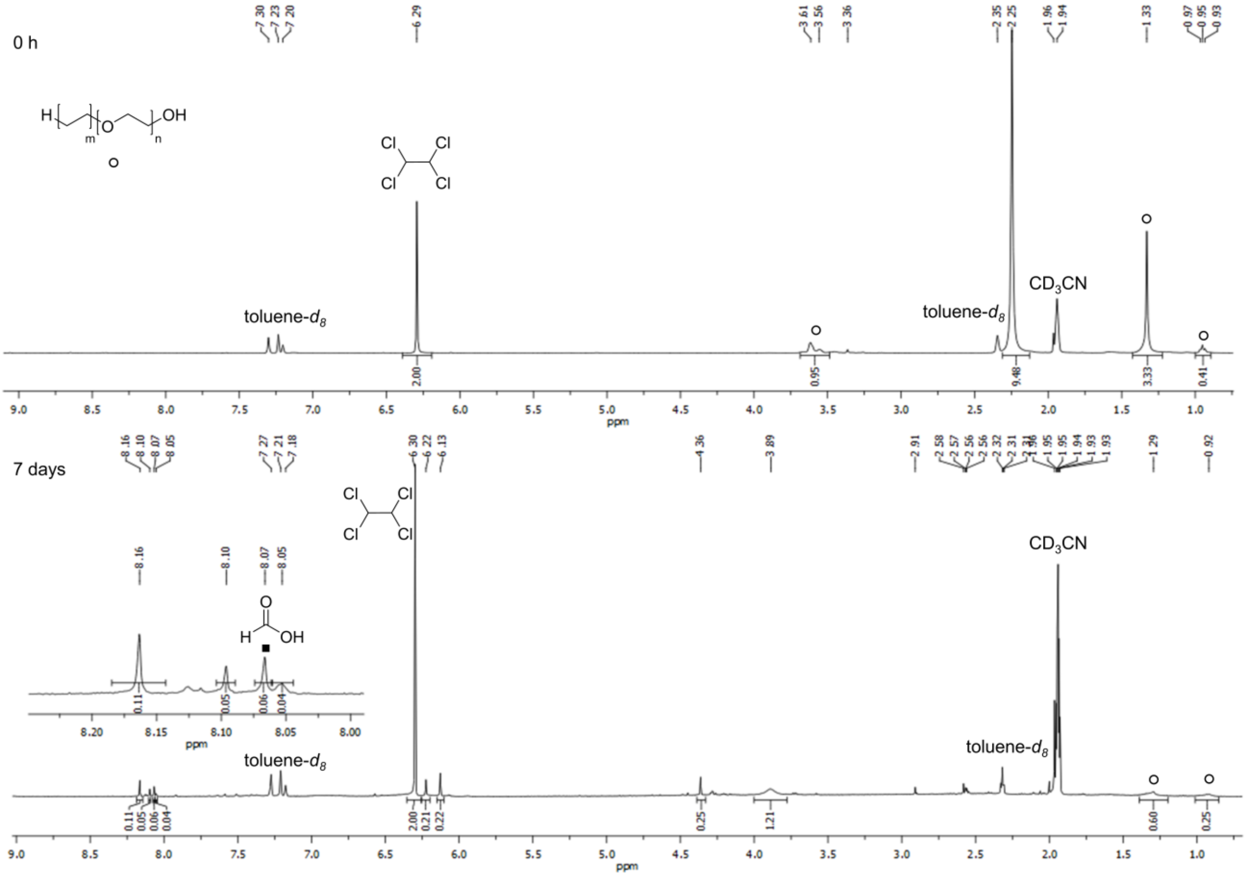
0 h
H O OH
m n
Cl Cl
Cl Cl
toluene-d8
toluene-d8
CD3CN
ppm
7 days
Cl Cl
Cl Cl
O
H OH
ppm
toluene-d8
toluene-d8
CD3CN
ppm

^{1}H NMR

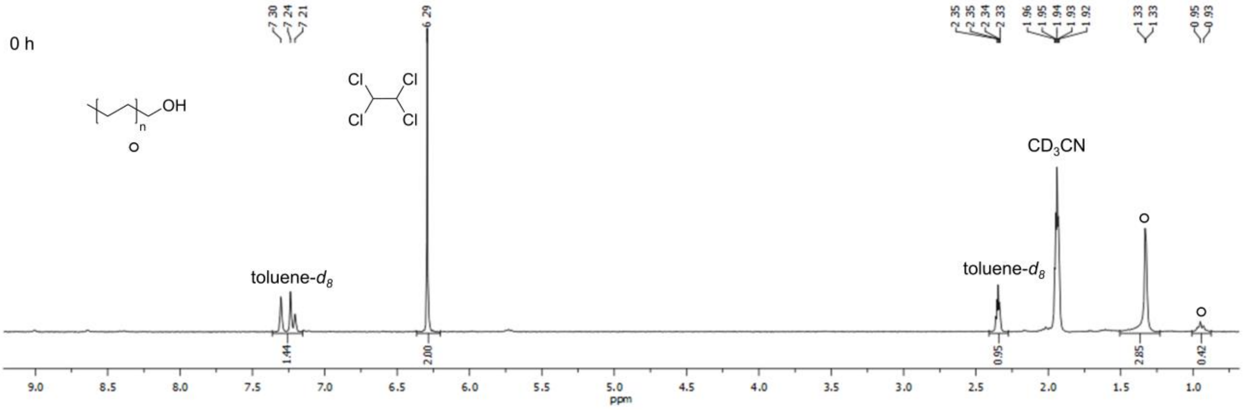
0 h
OH
n
Cl
Cl
Cl
Cl
CD$_3$CN
toluene-d_8
toluene-d_8
1.44
2.00
0.95
2.85
0.42
ppm

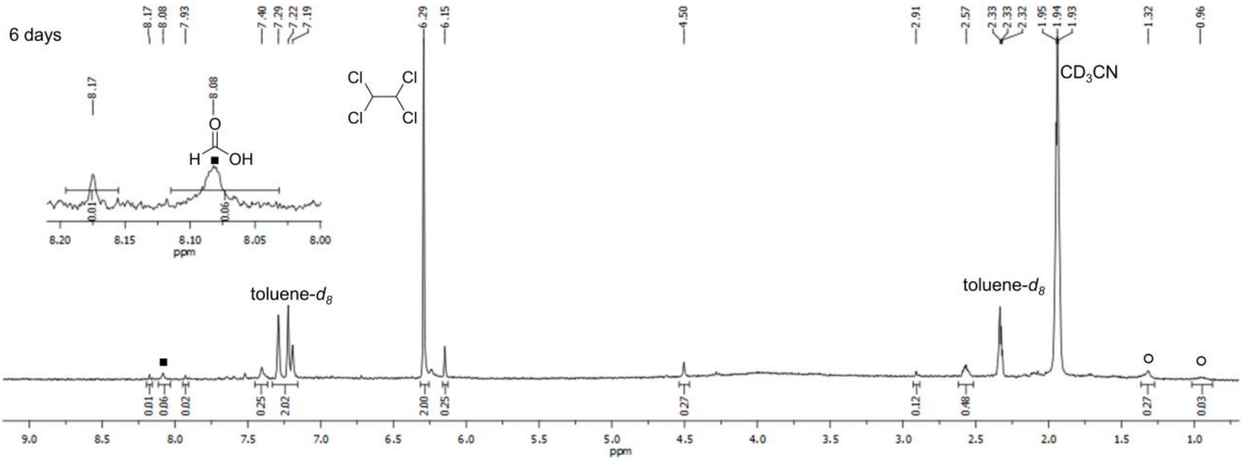
6 days
O
H
OH
Cl
Cl
Cl
Cl
CD$_3$CN
toluene-d_8
toluene-d_8
ppm
